# Longitudinal human transcriptomic and spatial gene profiling at the incisional edge during long surgical procedures

**DOI:** 10.1038/s42003-025-09366-0

**Published:** 2025-12-22

**Authors:** Matthew R. Sapio, Evelyn Li, Anthony F. Domenichiello, Taichi Goto, Dragan Maric, Allison P. Manalo, Tracy S. Williams, Saber Tadros, Jonathan M. Hernandez, David S. Schrump, Jeremy L. Davis, Andrew M. Blakely, Michael J. Iadarola, Andrew J. Mannes

**Affiliations:** 1 Department of Perioperative Medicine, National Institutes of Health, Clinical Center, Bethesda, MD USA; 2 Lipid Peroxidation Unit, Laboratory of Clinical Investigation, National Institute on Aging, National Institutes of Health, Baltimore, MD USA; 3 Office of Pain Policy and Planning, National Institute of Neurological Disorders and Stroke, National Institutes of Health, Bethesda, MD USA; 4 Symptoms Biology Unit, National Institute of Nursing Research, National Institutes of Health, Bethesda, MD USA; 5 Flow and Imaging Cytometry Core Facility, National Institute of Neurological Disorders and Stroke, National Institutes of Health, Bethesda, MD USA; 6 Department of Pathology, Microbiology and Immunology, University of Nebraska Medical Center, Omaha, NE USA; 7 Surgical Oncology Program, Center for Cancer Research, National Cancer Institute, Bethesda, MD USA; 8 Thoracic Surgery Branch, Center for Cancer Research, National Cancer Institute, Bethesda, MD USA

**Keywords:** Experimental models of disease, Transcriptomics, Pain

## Abstract

Post-surgical pain remains a widespread problem reducing quality of life. The present study investigates the initial molecular changes underlying nociceptive sensitization through longitudinal, temporal sampling at the surgical wound edge. Using RNA-Seq and multiplex fluorescence in situ hybridization, we examined the most significant genes induced by tissue injury including those coding for the secreted factors interleukin 6, oncostatin M and leukemia inhibitory factor, and localized these induction events to several cutaneous structures including the epidermis, vascular endothelia, hair follicles, and sweat glands. Our data also demonstrate the receptors for these key secreted factors are expressed by dorsal root ganglion neurons, indicating long-range signaling from damaged skin to spinal cord, thereby leading to pain. This study provides a novel understanding of tissue structures and the molecular interactome activated following tissue injury by elucidating the inflammatory and tissue repair transcriptional milieu induced by surgery in human skin excision biopsies.

## Abstract (web summary)

Transcriptomic profiling of human skin serially sampled from wound margin during long surgeries reveals tissue damage and inflammation-related gene signatures relevant to human post-operative wound recovery and postoperative pain.

## Introduction

In the past two decades, acute pain in the first week after surgery has been increasingly managed using strong opioids and has been associated with opioid misuse and opioid use disorder^1,2^. It has been suggested that opioid prescribing after surgery is related to opioid use disorder, with an increased number of prescription refills increasing the likelihood of opioid use disorder^2^. Simultaneously, pain after surgery is a major concern, and can become chronic in ~10% of cases^1^, leading to the development of chronic post-surgical pain that interferes with quality of life. Ultimately, two major goals have arisen to address these concerns. One goal is to achieve adequate post-surgical pain control without reliance on opioids, and a second, related goal is to understand the etiology and evolution of post-surgical pain to design strategies to prevent it. It is clear that several elements induced by tissue damage during surgery contribute strongly to the development of pain, presumably via the release of inflammatory mediators associated with tissue damage, which stimulate and sensitize the nociceptive apparatus^1,3^. Sensitized nociceptors may manifest as exhibiting several criteria, including spontaneous activity, decrease in response threshold, enhanced responses to suprathreshold stimuli, and expansion of receptive field sizes, all of which contribute to pain after surgery^3,4^. Presumably, this comes about in part because the nociceptive afferent ending is bathed in a complex mixture of damage-associated molecular patterns (DAMPs), e.g., signals coming from damaged tissue, including inflammatory mediators^5,6^, and direct sensing of molecules released from damaged cells, such as ATP^7^. The present study seeks to understand the full complement of secreted factors released acutely from surgical wound tissue, to understand how these factors signal to nociceptive afferents.

Previously, we published longitudinal studies in two rat models examining early signaling in response to incision and inflammation^5,8^. The incision model, which is a common rodent model of surgical incisional pain, involves incision of the glabrous skin of the paw, leading to nociceptive sensitization and hyperalgesia^9^, but has limited application to human wounds, as there are marked differences between wound healing strategies and skin characteristics between rodents and humans. This underscores the importance of extending these results to human surgical tissue samples to ensure human relevance. The present study examines thoracic and/or abdominal surgical procedures in order to capture the acute responses to tissue injury in participants undergoing long surgeries (≥4 h). Samples were taken at 0 h (initial incision), 1 h, 2 h, 4 h, 6 h, and at the closure of the surgical wound if the surgery lasted ≥8 h. Each sample was analyzed using (a) histology, (b) multiplex fluorescent labeling, and (c) RNA-Seq to understand the full signaling cascades during this early post-injury epoch. In addition, we characterized patient-reported outcomes of pain before surgery and postoperatively at 1 and 2 days using the Brief Pain Inventory and McGill pain scales to understand the pain state of each patient. The present study seeks to obtain a longitudinal transcriptional landscape of surgically incised skin to comprehensively understand how DAMPs initiate pain and inflammation, activate wound healing processes, and understand which of these signals could be capable of signaling to DRG neurons. This early step of signal transduction is the “beginning” of the pain pathway and is critical for understanding the nociceptive process occurring at peripheral afferent nerve endings.

## Results

### Patient-reported outcomes (PROs) of pain

**Fig. 1:**
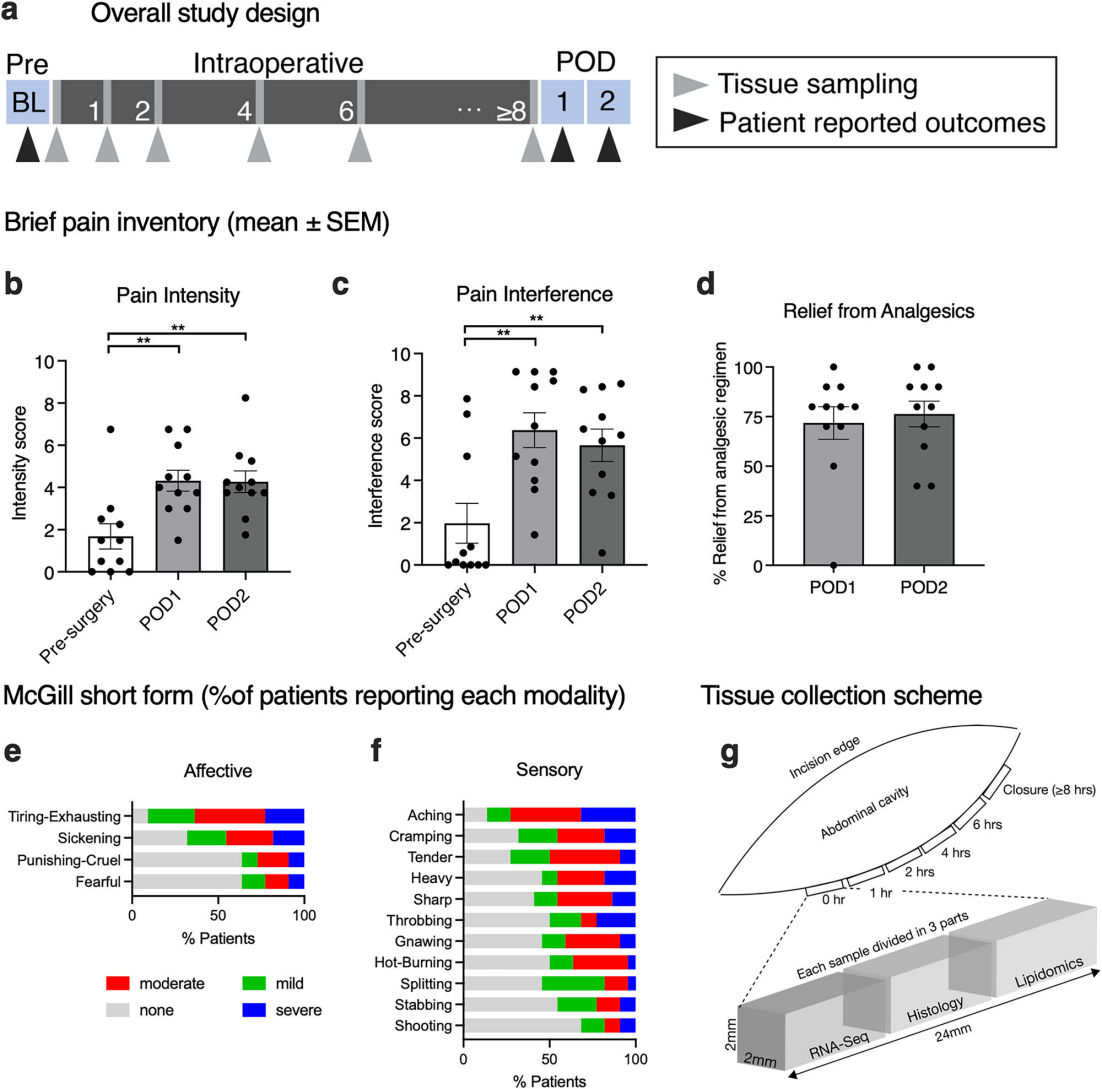
Study overview and clinical pain phenotyping for subjects in the tissue collection protocol. **a** An overall summary of the schedule of activities shows that each patient was evaluated for patient-reported outcomes of pain pre- and post-surgery at postoperative (POD) days 1 and 2. **b** The Brief Pain Inventory scale was administered to assess pain intensity and **c** interference (all error bars show mean ± SEM). These data indicate that participants generally entered the study without, or with mild pain, and significantly increased by POD1 and POD2, where they experienced moderate to severe pain in the postoperative period. **d** Participants also reported significant relief from analgesics given as part of standard of care (Friedman test, followed by Dunn’s multiple comparison testing, Prism 10; *, *p* < 0.05; **, *p* < 0.01). **e** Postoperative pain scores were also collected using the McGill pain scale, which asks about specific pain descriptors and is aimed at assessing pain modalities. Affective and sensory pain descriptors in the postoperative period indicated that tiring-exhausting and sickening pain were the most represented affective components, consistent with visceral pain after abdominal surgery. **f** Among terms indicating sensory modalities, patients reported descriptors such as aching, cramping, gnawing, tender, and heavy and sharp, consistent with pain from this type of tissue injury. **g** A diagram of intraoperative tissue collection shows how samples were collected and processed, with 24 mm long biopsies removed with a surgical scalpel at the specified time points and processed for molecular analyses using RNA-Seq, histology, and lipidomics (not included in this report).

PROs of pain were collected before surgery and at postoperative days (POD) 1 and 2 (Fig. 1a) using The Brief Pain Inventory^10^ (BPI) and McGill Pain Questionnaire (MPQ) to assess the amount of pain intensity and pain interference experienced after surgery. Participants (see demographics in Table 1) entered the study with ratings of either mild or no pain as indicated by the low values at the preoperative time point (Fig. 1b). After surgery, participants reported mild to moderate pain with pain intensity rating (mean ± SEM) rising from an average of 1.7 ± 0.6 at baseline to an average of 4.3 ± 0.5 at POD1 (*p* = 0.004, Z = 3.1) and 4.3 ± 0.5 at POD2 (*p* = 0.0019, Z = 3.3), consistent with a moderately painful procedure (Friedman test followed by Dunn’s multiple comparison testing for repeated measures, Prism 10; *, *p* < 0.05; **, *p* < 0.01; Fig. 1b). Pain interference (mean ± SEM) similarly increased from 2.0 ± 0.9 at baseline to 6.4 ± 0.8 at POD1 (*p* = 0.0013, Z = 3.4) and 5.7 ± 0.8 at POD2 (*p* = 0.0057, *Z* = 3.0); Friedman test followed by Dunn’s multiple comparison testing for repeated measures, Prism 10; *, *p* < 0.05; **, *p* < 0.01; Fig. 1c. Patients reported 72% relief from painkillers at POD1 and 76% relief at POD2 (Fig. 1d) indicating standard of care pain management was successful. Pain modalities and specific pain descriptors for post-surgical pain were also evaluated using the McGill pain scale^11^. The most common affective components that were reported by patients included tiring-exhausting (91%) and sickening (68%) pain (Fig. 1e), while sensory modality descriptors that were highly represented were aching (86.36%), cramping (68.18%), tender (72.73%), heavy (54.55%) and sharp (59.09%; Fig. 1f). Individual components of the composite scores are reported in Supplementary Figs. 1 and 2.

**Table 1:** Demographics

Gender—No. (%)	
Female	7 (58.3%)
Male	5 (41.7%)
Age median[range]	64 [25–76]
Race—No. (%)	
White	10 (83.3%)
Black	1 (8.3%)
Other/missing	1 (8.3%)
Ethnicity—No. (%)	
Hispanic	1 (8.3%)
Non-Hispanic	11 (91.7%)
Surgery location—No. (%)	
Abdominal	11 (91.7%)
Thoracic	1 (8.3%)
Surgical duration—No. (%)	
≤4 h	1 (8.3%)
4–6 h	3 (24.9%)
6–8 h	3 (24.9%)
≥8 h	5 (41.5%)
ASA class^a^—No. (%)	
III	11 (91.7%)
IV	1 (8.3%)
Intraoperative chemotherapy—No. (%)	4 (33.3%)

^a^American Society of Anesthesiologists (ASA) class III is defined as a patient with severe systemic disease. ASA class IV is defined as a patient with severe systemic disease that is a constant threat to life.

### Overview of tissue collection scheme

A diagram of intraoperative tissue collection procedures shows how samples were collected and processed. At each of the specified time points, a single 24 mm long biopsy was removed with a surgical scalpel and processed by dividing into three sections: one for molecular analyses using RNA-Seq, a second for histology (Fig. 1g), and a third for lipidomics, which is not included in this report. Exact times of tissue collection are described in Supplementary Fig. 3.

**Fig. 2:**
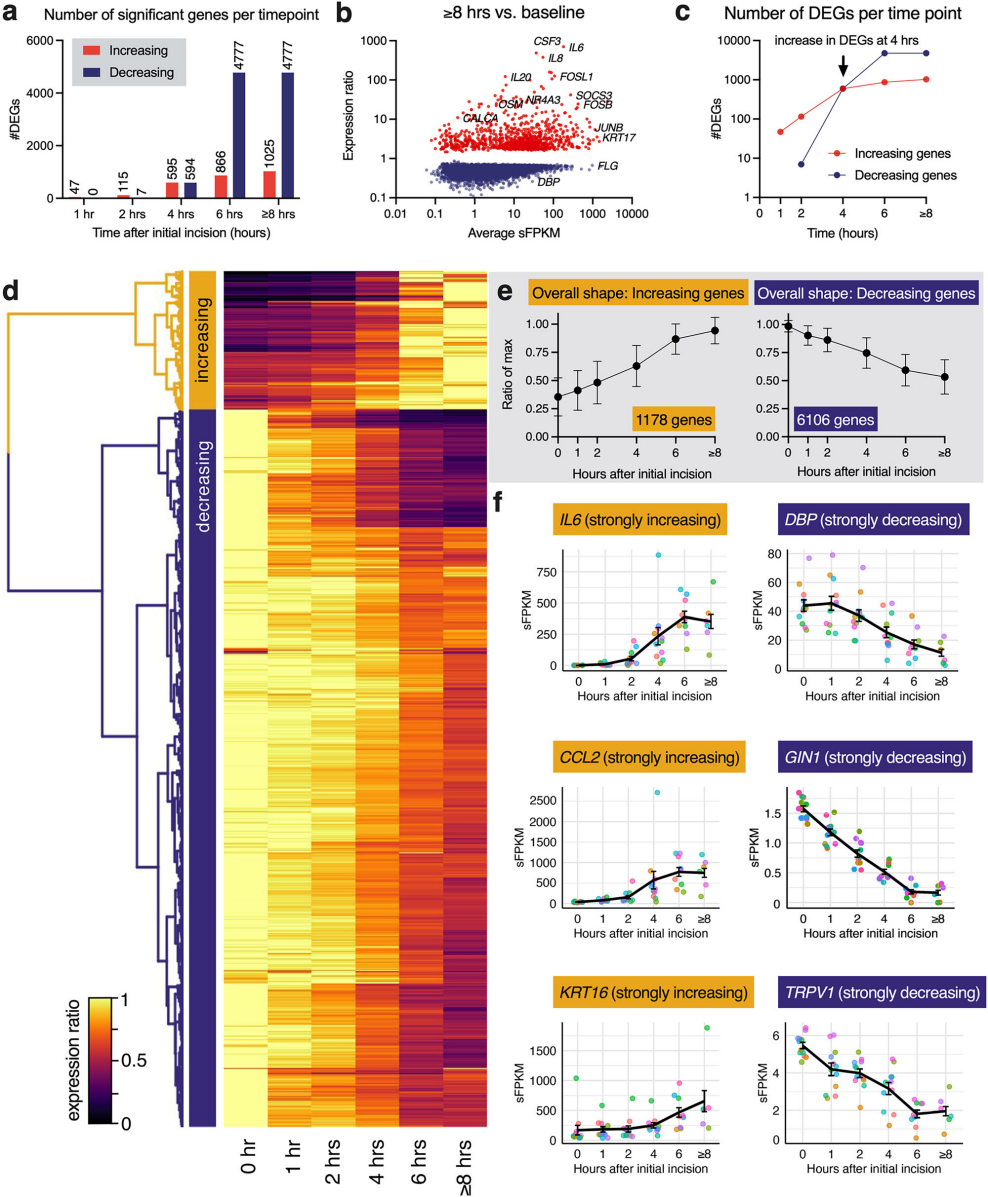
Overall patterns of skin gene expression during long-duration surgical procedures. **a** Total number of significantly differentially expressed genes (DEGs) between time zero and each time point were determined in MAGIC and continually increased over the course of the experiment. Note the higher number of significantly decreasing genes (4777 DEGs) at 6 h and closure. **b** A scatter plot was made to examine the expression level and fold change for the genes at the ≥8 h (closure) time point. **c** The overall pattern of DEGs increases markedly at 4 h for both increasing and decreasing DEGs. **d** Significantly differential genes were normalized to values between 0 and 1 relative to the maximum value at any time point (see flame scale), and a heatmap was constructed examining all DEGs across time points, and the clustered using k-means clustering. The two major clusters corresponded to increasing and decreasing genes, implying that there are no fine-grained patterns, such as genes that change direction during the time course, and the general pattern of expression shows a continual increasing magnitude over the time points examined. **e** These two patterns are summarized by averaging the normalized shape of each curve and plotting for an aggregate of all DEGs in each pattern, resulting in 1173 increasing DEGs and 6106 decreasing DEGs (mean ± SD). **f** For increasing DEGs, we selected two of the highest magnitude increasing DEGs: Interleukin 6 (*IL6*) and monocyte chemoattractant protein-1 (*MCP-1*, also known as *CCL2*) to plot individually. Additionally, to give an example of a moderately increasing gene, we plotted the longitudinal data for Keratin 16 (*KRT16*). Among the decreasing genes, we selected D-box binding PAR bZIP transcription factor (*DBP*) and Gypsy Retrotransposon Integrase 1 (*GIN1)*, which decrease strongly in the dataset. Additionally, we include transient receptor potential, vanilloid 1 (*TRPV1*) as an example of a moderately decreasing gene. These examples are meant to show the representative examples of the overall pattern among these significant genes in both directions. Error bars show SEM.

RNA-Seq was performed on a total of 63 samples at a read depth of 52.9–94.8 million reads/sample (average of 71.1 million reads/sample). RNA-Seq raw counts and significant fragments per kilobase of transcript per million mapped reads (sFPKM) quantification data are provided in Supplementary Tables 1 and 2. After determination of the total number of differentially expressed genes (DEGs) using MAGIC^12,13^, significantly increasing and decreasing genes were plotted per time point relative to the time zero control (Fig. 2a). The total number of unique genes significantly increasing at any time point was 1163. Note that the number of significantly decreasing DEGs at the 6 h and closure time point (4777 DEGs) is much larger than the number of increasing DEGs. Scatter plot diagrams were created to visualize the most strongly differential genes at each time point by plotting adjusted expression ratio (*y*-axis) vs. expression in significant fragments per kilobase of transcript per million mapped reads (sFPKM; *x*-axis; Supplementary Fig. 4). This analysis emphasizes strongly detected genes with the largest expression changes, as described previously^12,14^. At the closure time point (≥8 h), 1025 genes were increased, and 4777 genes were decreased (Fig. 2b). The number of DEGs at each time point shows a notable inflection point at 4 h, as the number of DEGs detected at 4 h sharply increased (Fig. 2c). Significantly differential genes were clustered using a heatmap to identify temporal patterns, as described previously (Fig. 2d)^5,8^. The clustering analysis determined that most genes continuously increase or decrease throughout the time course sampled and generally do not show distinct temporal patterns. The average trajectory of increasing and decreasing genes was created by plotting the normalized shape across all increasing or decreasing DEGs, and both show a linear shape (Fig. 2e). The full list of DEGs for every time point is reported in Supplementary Table 3.

In total, 1178 increasing DEGs were identified, as well as 6106 decreasing DEGs. Note a small difference in the number of increasing DEGs between the clustering method and simply taking the unique sum of all genes found to be significant at any time point. This is due to a small number of genes that do not fit the general pattern, including *FOS*, which shows high and variable induction at several time points (highest at 1 h time point, increasing from 224.85 sFPKM at 0 h to 1671.30 sFPKM at 1 h, a 643.30% increase) but generally does not have a large impact on downstream analyses, which use the list of 1163 increasing DEGs. Much of the gradation across the heatmap represents magnitude, with the top of the heatmap (in which a dark purple/black color is observed in either the time zero or the last time point) being the highest magnitude changes. Three examples of increasing and decreasing genes are plotted (Fig. 2f). For increasing DEGs, we selected two of the highest magnitude increasing DEGs: interleukin 6 (*IL6*) and monocyte chemoattractant protein-1 (*MCP-1*, also known as *CCL2*). Additionally, to give an example of a moderately increasing gene, we plotted the longitudinal data for keratin 16 (*KRT16*), which was selected due to its relatively high basal expression and previous literature connecting it to wounding^15^. Among the decreasing genes, we selected D-box binding PAR bZIP transcription factor (*DBP*) and gypsy retrotransposon integrase 1 (*GIN1)*, two DEGs that have not previously been associated with wounding, but which decrease strongly in the dataset. Additionally, we include transient receptor potential vanilloid 1 (*TRPV1*) as an example of a moderately decreasing gene. This gene was selected to highlight an emerging role for this known DRG thermosensory channel in skin functions.

### Identification and pathway analysis for highly significant secreted factors induced by incision

Prior to analysis, the potential impact of sex differences on gene expression was examined and showed no significant difference between male (*N* = 5) and female (*N* = 7) incisional responses (Supplementary Figs. 5, 6 and Supplementary Note C1). Subsequently, analyses were performed on pooled male and female participants without consideration of sex. The 1163 significantly increasing genes from Fig. 2a were extracted and sorted according to maximum expression ratio relative to control, which generally occurred at 6 h or closure, and examined the broad categories of gene induction events induced by injury (Supplementary Fig. 5a). Chief genes that were strongly induced following incision were genes in the IL6 cytokine family, including *IL6* (775.1), *CSF3* (772.6), and *OSM* (30.8) and CXC chemokines, including *IL8* (377.9), *CXCL2* (174.0), and *CXCL1* (49.4), with maximum fold changes compared to the 0 hr time point indicated in parentheses (Supplementary Fig. 7 and Supplementary Note C2). Another small category of genes involving the metallothioneins (MTs) family showed high levels of expression. Metallothionein 2A (*MT2A*) was the highest expressed increasing DEG in the dataset, reaching a maximum expression level of 6611.5 sFPKM from 231.32 sFPKM at baseline, 2758.16% increase. Due to the profound induction of *MT2A*, and its capacity for binding zinc, an important metal ion for wound healing^16^, we further characterized the expression of this transcript, and its encoded protein in Supplementary Fig. 8.

**Fig. 3:**
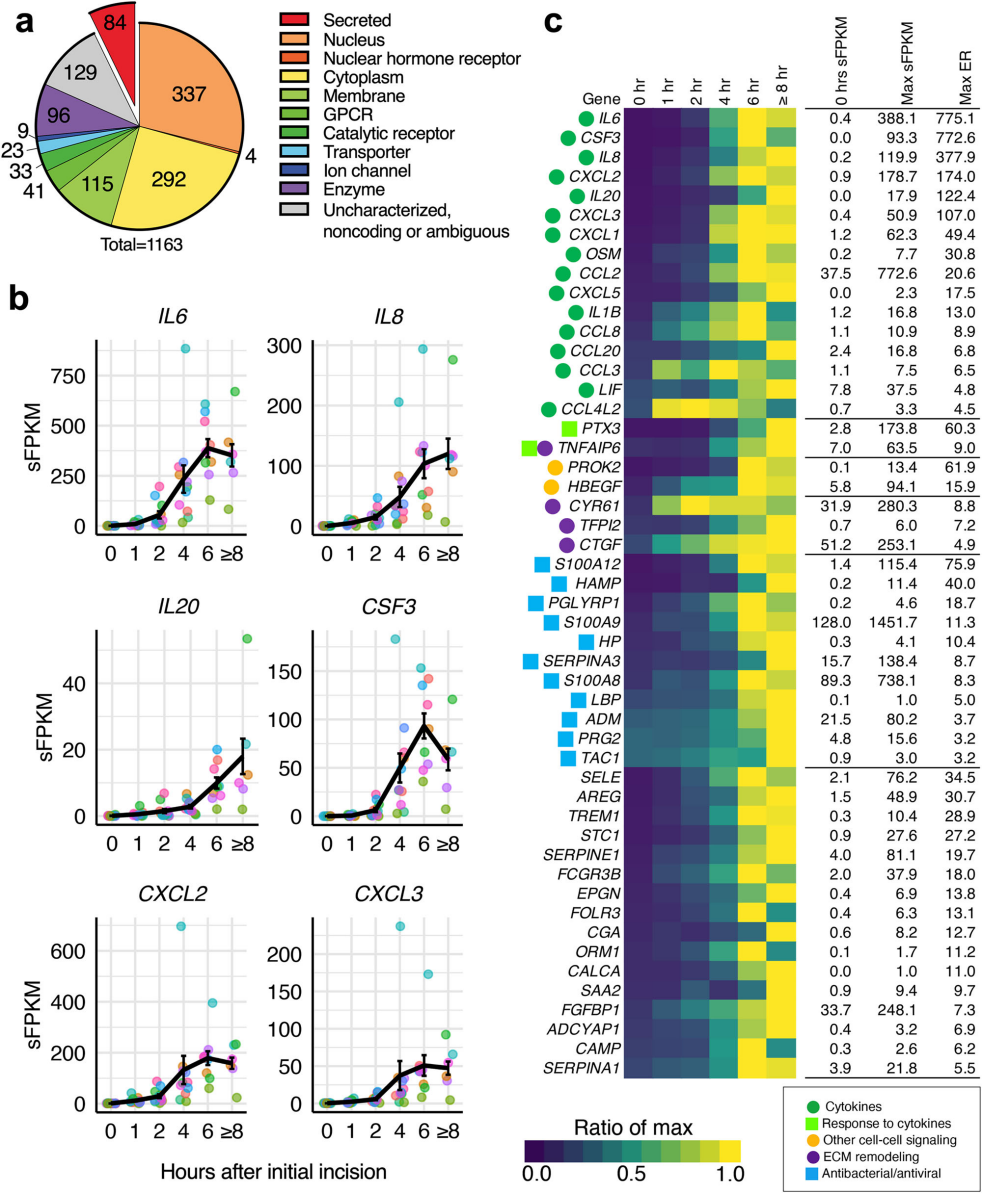
Extraction of the surgical incision-associated “secretome” from the most highly induced genes. In order to determine the secreted proteins that are most strongly induced by incisional injury, genes were categorized using queries to existing databases to classify them either into pharmacological categories (types of receptors, ion channels transporters commonly targeted in pharmacological experiments) or cellular compartments. **a** Of these categorized genes, genes encoding secreted proteins (red) were selected for further analysis (84 induced genes). **b** Examples of the most strongly induced secreted genes are plotted (mean ± SEM). **c** Genes over an expression threshold (lowest expression was *LBP*, 1.0 sFPKM) were sorted by expression ratio, and the top 50 genes were used to construct a heatmap where each row (gene) is normalized on a 0–1 scale (see flame scale). Error bars show SEM.

Given that the induction of secreted factors was highly prominent from an agnostic analysis, we further specifically assessed transcripts encoding secreted proteins. This was accomplished by subsetting the total significant gene list by categorizing all significantly increasing genes into functional and/or pharmacologically targetable categories, including secreted proteins, cellular components (cytoplasm, nucleus, and membrane), and receptors (Fig. 3a). Additional more granular categories of G-protein coupled receptors, and catalytic receptors are reported in Supplementary Fig. 9. Of the genes that coded for secreted proteins (84 genes), the top 50 genes were plotted in a heatmap by expression ratio of induction fold (Fig. 3c). Individual sFPKM plots of significantly induced genes, including *IL6*, *IL8*, *OSM*, and *OSMR* are shown in Fig. 3b. We further performed a bioinformatic clustering analysis using a skin dataset to assess what cell populations our top secreted genes belonged to (Supplementary Fig. 10). Expression of *IL6* was associated with vasculature including pericytes and endothelial cells, whereas *IL8* was associated with macrophage populations. Several genes, including chemokine ligands 1–3, were expressed mainly in fibroblasts. The categorization of all 1163 increasing genes is detailed in Supplementary Table 4.

### Use of quantitative pathway analysis tools to understand gene expression changes

Enrichr was used to identify broadly significant pathways among the induced genes (Supplementary Fig. 7b). The number one pathway identified (BioPlanet 2019 database) was “Interleukin-1 regulation of extracellular matrix,” which was highly significant at every time point examined (Supplementary Fig. 7b). Several other interleukin pathways were identified suggesting that interleukin signaling is a prominent feature of the cellular responses to incision. Oncostatin M signaling was also identified as highly significant at every time point examined, indicating an increase in Oncostatin M signaling. Note that among increasing gene pathways, most pathways are significant at every time point (non-significant genes shown with a white background, cutoff for significance, *p* = 0.01). We also note that while no sex differences were significant, *OSM* induction (alongside other genes such as *IL8*) was non-significantly stronger in females, and this could be further investigated in future studies (Supplementary Fig. 5).

By contrast, decreasing genes showed very little, if any, coherent pathway regulation using this method, with the major significant pathway being “generic transcription”, consistent with the idea that transcription is reduced over time, potentially by trauma (Supplementary Fig. 11). Because of this general lack of pattern among significantly decreasing genes, we focused mainly on increasing genes. Importantly, pathway analysis is imperfect in that often gene pathways can be related to each other, and not fully captured in these analyses. To address this, we performed STRING analysis on the top 100 significantly increasing genes, which indicated that the top 100 genes are highly interconnected in terms of function (Blue interconnected nodes, Supplementary Fig. 7c). This is consistent with the similarity of some of the most significant gene pathways and indicates that for the most part, a major gene regulatory pattern predominates post-surgically and that individual pathways are likely to be interrelated. Additional bioinformatic analyses of pathways per time point were performed in Supplementary Figs. 12, 13 and Supplementary Note C3.

### Neutrophil infiltration is a major component of the early immune response to injury

**Fig. 4:**
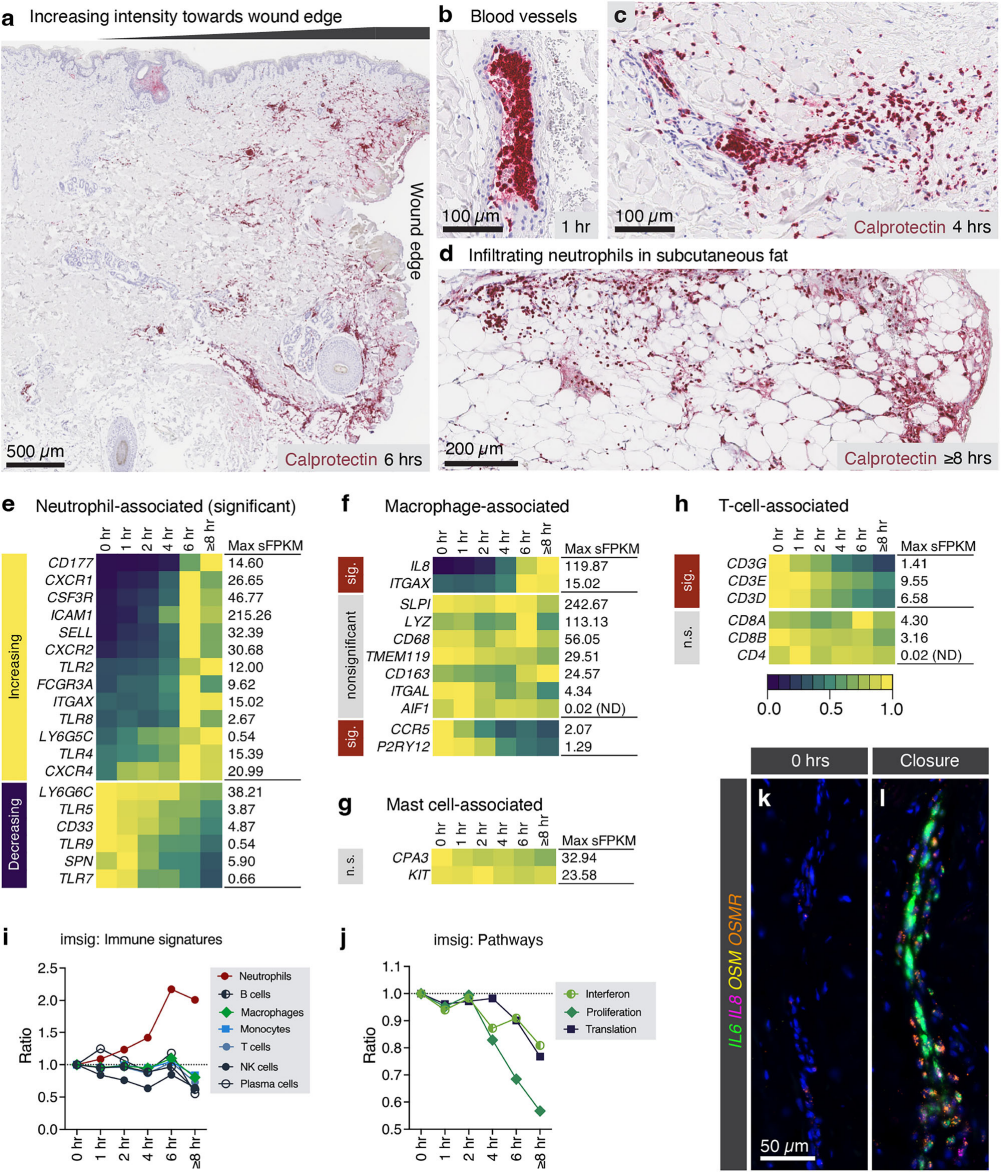
Neutrophil infiltration is a major feature of the early immune response to surgical tissue damage. **a** Based on existing literature and the observation that *S100A8* and *S100A9* are highly significant in the dataset and are very strongly expressed on neutrophils, we investigated the impact of infiltrating cells across the longitudinal time points of the study. Histologically, it was apparent that neutrophils were present sporadically in the tissue. Using a calprotectin antibody, we visualized neutrophils, which often appeared proximal to the wound edge. **b** Neutrophils also appeared inside the lumen of blood vessels, **c** and sometimes perivascular neutrophils were observed in proximity to a blood vessel. **d** In this example, neutrophils infiltrated the fat, representing their tendency to occupy certain areas of the tissue. **e** A broad set of marker genes was interrogated in the dataset. Note that most marker genes are not perfect for neutrophils and can be shared by other cell types. Nonetheless, more than half of these genes were significantly increased, including *CD177*, which is probably one of the most specific neutrophil markers. **f** By contrast, while *IL8* and *ITGAX* are associated with macrophages, the more specific marker gene panel generally showed no change. This was also true of **g** mast cell markers and **h** T-cell associated transcripts. **i** Using imsig analysis, these results were also confirmed, suggesting that neutrophils are the only increasing immune population, with other immune populations static or decreasing. **j** This analysis also suggested that proliferation and translation generally decreased in the dataset, perhaps explaining the lack of specificity of the large number of significantly decreasing genes. **k** In basal state, many of these genes are not readily detectable in anatomical analyses, but **l** are strongly induced around vascular endothelial cells, which was often more evident than presence in leukocytes themselves. This points to complex patterns of induction in both leukocytes and vasculature.

In the hind paw incision model in rodents, the first immune recruitment event occurred at 4–6 h after incision, when neutrophils were recruited to the wound site^5,8^. Previous literature, gross histology, and several of the significant genes, such as *CD177*, *S100A8*, and *S100A9*, were suggestive of early neutrophil infiltration, consistent with the first phase of immune response to injury. Using a calprotectin antibody (which labels the dimerized gene products of *S100A8* and *S100A9*), we observed neutrophil enrichment nearer to the wound edge in most samples (Fig. 4a) and increasing steadily over the course of the procedure. Note that in human skin, using in situ hybridization, *S100A8* and *S100A9* also labeled epidermal cells, particularly near hair follicles, in contrast to the protein staining which is relatively specific for neutrophils (Supplementary Fig. 14). Neutrophils appeared inside the lumen of blood vessels (Fig. 4b), and often also perivascular interstitial neutrophils were observed near blood vessels (Fig. 4c), suggestive of transmural migration. Neutrophils often infiltrated structures, such as hair follicles or large regions of fat. An example of neutrophils infiltrating a large mass of subcutaneous fat is shown in Fig. 4d. To address the presence of various immune cells infiltrating, we examined marker genes based on existing literature. For neutrophils, some of the most specific marker genes, such as *CD177*, were significantly increased (0.29 sFPKM to 14.60 sFPKM, 4934.48% increase), following the same pattern as *S100A8*, for example (Fig. 4e). In general, more than half of the neutrophil panel genes were increased. By contrast, while *IL8* and *ITGAX* are associated with macrophages, the more specific marker gene panel generally showed no change (Fig. 4f). Indeed, panels designed to examine macrophages (Fig. 4f), mast cells (Fig. 4g), or T-cells (Fig. 4h) did not reveal obvious increases, although there are relatively few specific mast cell genes, and bulk tissue transcriptomics has limitations for examining cell type specific changes. Another approach to measure this was to use the computational package imsig to quantify immune cell signatures over time. This approach uses validated marker panels to estimate the abundance of cell types in a tissue sample. The imsig analysis confirmed that neutrophils, but not other major immune cell types, were increased after surgery (Fig. 4i). Additionally, imsig suggested that broad pathways of proliferation and translation were decreased (Fig. 4j), which could explain the large number of relatively non-specific decreasing DEGs. Notably, the highly induced gene *IL6*, which participates in immune cell migration, was most strongly induced in endothelial cells, underscoring that in some cases, immune signaling involves diverse cell types (Fig. 4k, l).

### Assessment of oncostatin M and oncostatin M receptor gene expression in skin and DRG neuronal populations

**Fig. 5:**
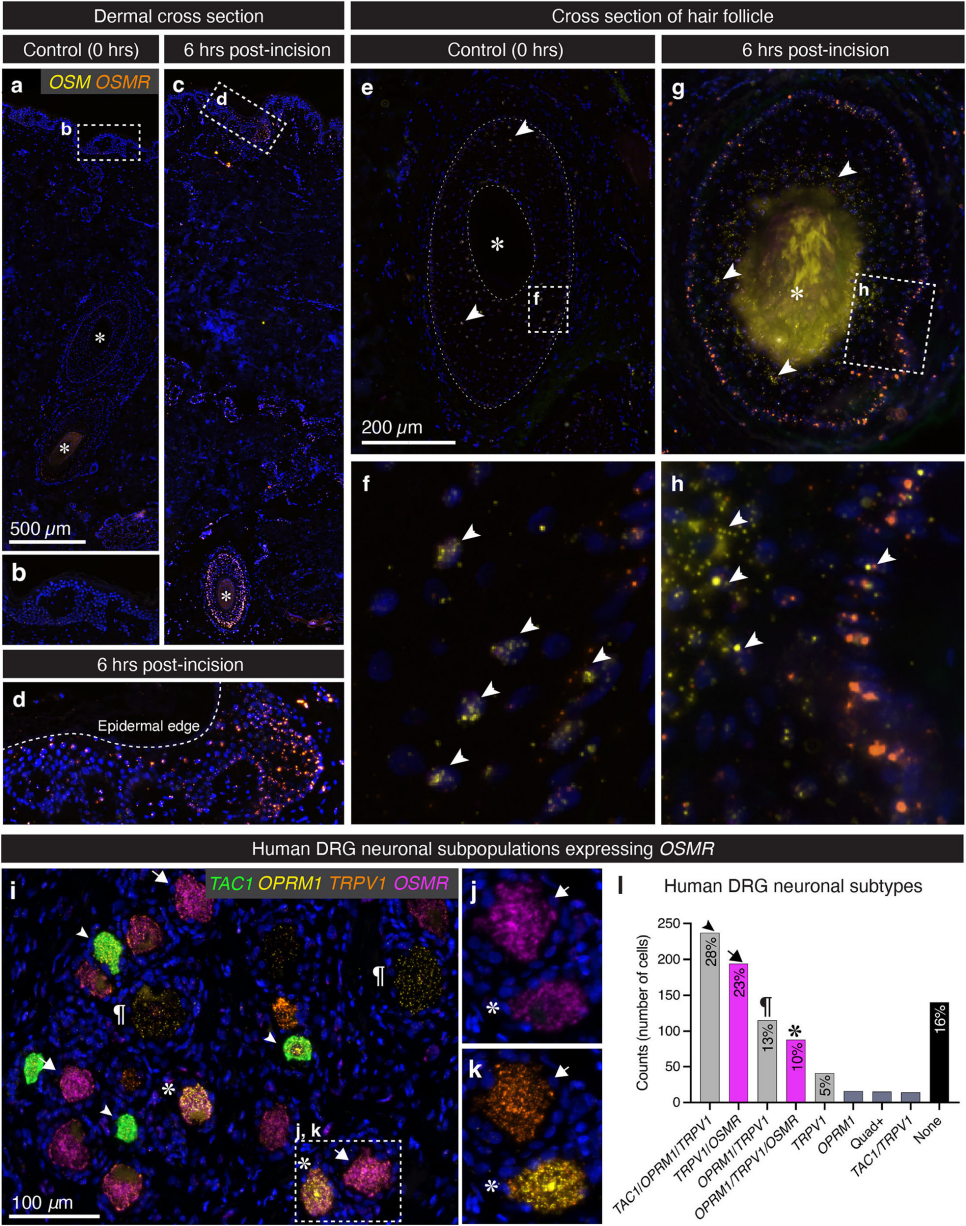
Anatomical correlates of pleiotropy in OSM-OSMR pathway signaling after surgical incision. In order to address the anatomical localization of induced genes, *OSM* and *OSMR* were stained via in situ hybridization. **a** A cross-section spanning epidermis to deep dermal tissue is shown for the 0 h time point (enlargement of epidermis shown in (**b**)) collected at surgical onset. Expression of *OSM* and *OSMR* is detectable, but low. Asterisks mark hair follicles, which if a hair is present, display bright autofluorescent signal **c** A cross section from the same patient at 6 h demonstrates *OSMR* induction in various structures. **d** In an enlargement, a localized area of epidermis shows a bright *OSMR* signal. **e** A cross-section of a hair follicle at baseline showed very little signal (outlined for visibility). **f** An enlarged view of the hair shows *OSM* (arrowheads) and *OSMR* expression at baseline in the cells around the hair shaft. **g** Bright induction of *OSMR* and *OSM* is apparent in the cells around the hair shaft after 6 h. **h** In an enlargement, the staining pattern is apparent with *OSMR* at the edge of the hair structure, and *OSM* more proximal to the hair. **i** A representative field is shown of human DRG tissue stained for *TAC1*, *OPRM1*, *TRPV1*, and *OSMR* transcripts (merged image). The two major populations expressing *OSMR* are labeled with asterisks and arrows. **j** A view of the magenta (700 nm) channel alone shows intense *OSMR* staining in these two cell types. **k** However, they are differentiated by the presence or absence of *OPRM1*. **l** Quantification of the major cell types revealed by this staining combination revealed that *OSMR* is present in *TRPV1* + /*TAC1*− neuronal subpopulations, with (23%, arrows) or without *OPRM1* (6-pointed asterisk). A total of *n* = 861 cells in *N* = 4 human individuals were counted for this analysis.

Localization of *OSM* and *OSMR* transcripts in skin was addressed using multiplex fluorescent in situ hybridization. Previous studies suggest pleiotropic functions of OSM-OSMR signaling, including pain, itch, wound healing, and modulation of inflammatory pathways^17–19^. Therefore, we investigated which cell types within the complex tissue were involved in these processes. Induction of both *OSM* and *OSMR* was evident throughout skin tissue (see low-powered fields of skin tissue in Fig. 5a, b). Enrichment was notable within epidermal tissue (enlarged in Fig. 5d) as well as cells within hair follicles (Fig. 5c, asterisk). Hair follicles were seen throughout the skin (noted with asterisks in Fig. 5a, c, e, g), and were densely stained for *OSM* and weakly stained for *OSMR* in baseline (Fig. 5e, f, arrowheads). The hair follicle at the 0 h time point is outlined (dashed line) for visibility. However, after 6 h, induction of *OSM* and *OSMR* was prominent (Fig. 5g, enlarged in Fig. 5h), with *OSM* proximal to the hair and *OSMR* prominent around the structure. Note that this area is innervated by Calcitonin gene-related peptide precursor (*Calca*)+ circumferential endings responsible for sensing noxious hair pull (mechanonociception)^20–22^. This indicates that hair follicle structures are a major nexus of *OSM* induction and response. This structure is notable in that it contains a large number of stem cells, which may be responsible for some of this induction (Supplementary Fig. 15).

The oncostatin M pathway is prominently represented in human skin, as demonstrated by our anatomical evidence of expression in several skin structures (Fig. 5a–h). However, oncostatin M is also thought to signal to DRG sensory neurons, presumably by stimulating local long-range axons expressing *OSMR*^18,19^. In order to formally address the connection between *OSM* synthesis and release from damaged skin, and the stimulation of DRG sensory neurons, we performed in situ hybridization to determine if *OSM* could signal directly to nociceptive neurons via *OSMR* and establish which neuronal subpopulations are sensitive to this signal. Using 4-plex combinatorial labeling for the oncostatin M receptor (*OSMR*) alongside transient receptor potential vanilloid 1 (*TRPV1*), tachykinin 1 (*TAC1*), and the mu-opioid receptor (*OPRM1*), we identified several neuronal subpopulations containing *OSMR*. *TRPV1* is an ion channel that is sensitized by noxious heat stimuli and contributes to pain sensation, and can be used in combination with other markers to identify nociceptive primary afferents^23–26^. Because *TRPV1* is widely expressed across various classes of nociceptive neurons, we also included *TAC1*, which encodes for substance P, a key neuropeptide involved in pain signaling when transmitted from peripheral nociceptors to the dorsal horn of the spinal cord^25,27^. We also stained for *OPRM1*, which encodes the receptor responsible for mu-opioid analgesic agonizts such as morphine, and which is an important functional marker in human DRG^28^. This combination of probes strongly labels most DRG neurons (Fig. 5i–l, ~84% labeled), as described previously^25,28^. Note that *OPRM1* was not detectable in human skin in our study. Our staining revealed that *OSMR* was expressed in nociceptive neurons responsive to opioid analgesia (*OPRM1*+/*TRPV1*+/*OSMR*+; 10% of neurons, 6-pointed asterisks, Fig. 5i–l), as well as a second population that does not express the µ-opioid receptor (*TRPV1*+/*OSMR*+; 23%, arrows). Another feature of this staining combination was that *OSMR* was almost never co-expressed with *TAC1* (~2% of neurons). A small number of cells (*n* = 4) contained extremely high levels of *TRPV1*, consistent with previous observations. These rare neurons are small-diameter cells that may be dedicated thermosensory neurons and were *OSMR*-/*OPRM1*+, consistent with previous descriptions of this rare population^24,25^.

### Localization of chemokine induction to sweat glands following tissue injury

**Fig. 6:**
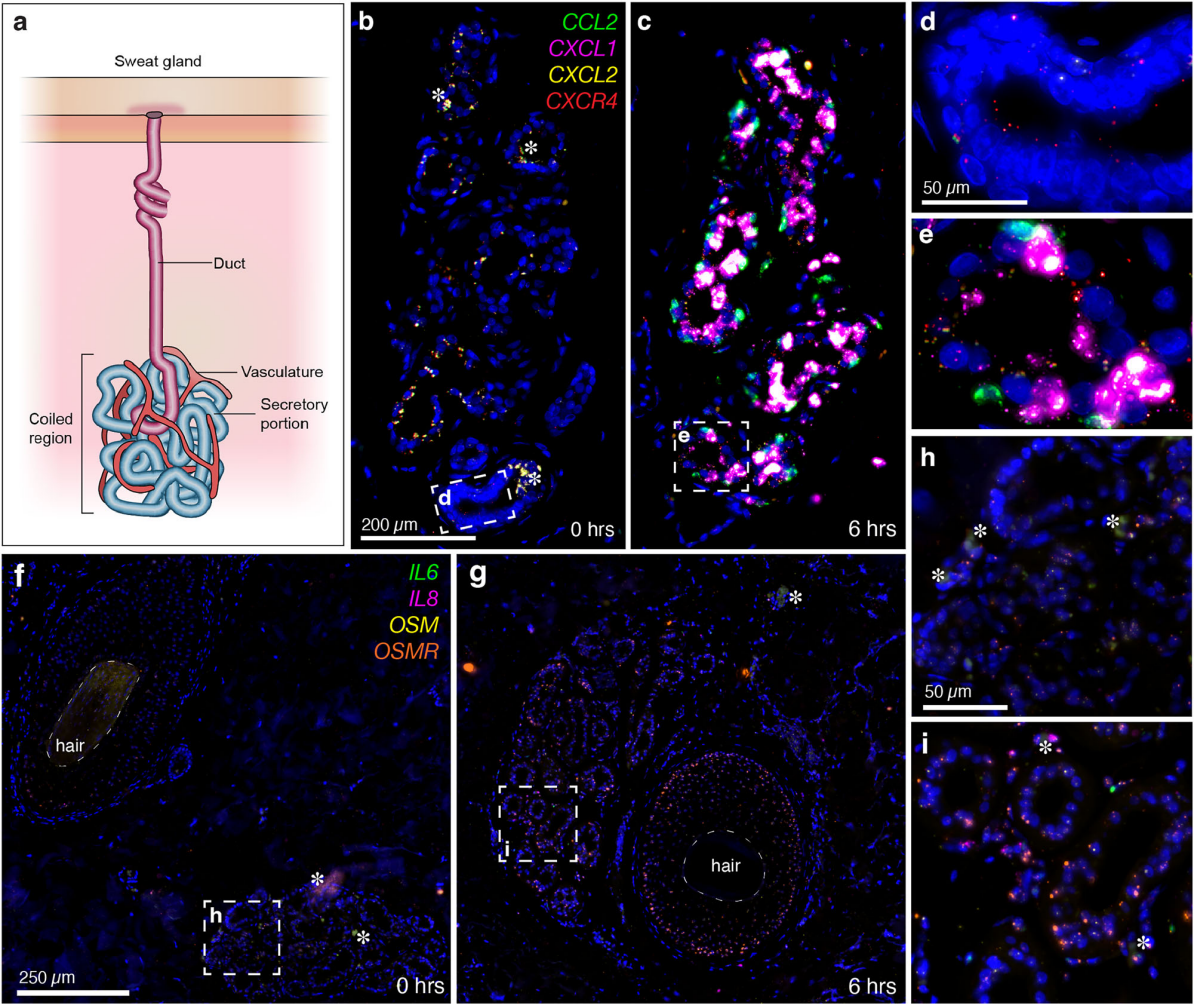
Chemokine induction localized to the sweat gland-vasculature interface after surgical incision. Several of the most highly significant cytokine signaling pathway genes were induced in and/or near sweat glands. **a** An illustration shows that a sweat gland consists of a coiled region and a secretory portion, with a duct opening to the surface of the skin for sweat release. Blood vessels also wrap around sweat glands to form a gland-vasculature interface. **b** A cross-section showing a sweat gland at 0 h in tissue stained for *CCL2*, *CXCL1*, *CXCL2*, and *CXCR4*. *CXCR4* is lowly expressed at baseline, while the other markers are not detected (enlargement in (**d**)). **c** A cross-section of a sweat gland at 6 h post-incision shows strong induction of *CCL2* and *CXCL1* in the gland structure (enlargement in (**e**)). **f** A cross-section of a tissue at 0 h showing a hair follicle (left) and a sweat gland (right) staining for *IL6*, *IL8*, *OSM*, and *OSMR*. In time zero samples, *OSMR* is the only marker that is expressed at low levels in the hair follicle and in the sweat gland (enlarged in (**h**)). Asterisks mark autofluorescence from red blood cells. Also note that real signal for *OSM* is present in **f** within the highly autofluorescent hair shaft. **g** A cross-section of a sweat gland (left) and hair follicle (right) in tissue collected 6 h after incision. *OSMR* expression is observed encircling the hair follicle and also throughout the gland. *IL6* and *IL8* are expressed to a lesser degree in the sweat gland (enlarged view in (**i**)).

In situ hybridization staining was performed to understand the anatomical localization of gene induction for a subset of the most strongly induced chemokine signaling genes, including *CXCL1*, *CXCL2*, *CCL2*, *IL6*, *IL8*, *OSM*, and *OSMR*, in two 4-plex investigations. Induction of these markers was observed in interconnected glandular and vascular structures that formed a sweat gland-blood vessel interface (Fig. 6a). Our staining revealed that *CXCR4* is expressed at low levels in the basal state, while chemokines *CCL2* and *CXCL1* are strongly induced in the sweat gland (Fig. 6b, c) after incision. In particular, the molecular activation of these chemokines occurs in the secretory coils of the sweat gland (Fig. 6d, e). Similarly, other chemokine markers like *OSMR* are induced in the secretory portions of the sweat gland. To a lesser extent, *IL6* and *IL8* are also induced in this structure (Fig. 6h, i). Interestingly, sweat glands have been suggested to contribute to wound healing and give rise to epidermal outgrowths during reepithelialization^29^. While these findings are largely unexplored, several studies support the role of these structures in damage responses (Supplementary Note D1).

### Delineation of local skin-skin signaling pathways and long-range interactions between injured skin and sensory neurons: the injury interactome

Upon tissue damage, DAMPs are activated to respond to injury, initiate inflammatory processes, and commence wound healing and skin regeneration. Molecules secreted by injured tissue participate in both local signaling to the skin, immune system, and long-range signaling by sensory neurons. This signaling milieu can be thought of as an “interactome” with secreted factors and receptors interacting across cell types as part of the complex response to tissue injury. Generally, we subdivided the interactome into the following subcomponents: (1) skin-skin local interactions, and (2) skin-to-nerve long-range interactions. It is also notable that these interactions can be further subcategorized by particular cell types, including immune cells, neurons, and keratinocytes. However, many pathways appeared to involve combinatorial participation of several distinct cell types.

Having established that surgical incision prompts the induction and secretion of various inflammatory and immune factors, we further evaluated the potential of these gene products to signal to DRG sensory neurons to affect downstream nociceptive signaling. We selected the top secreted transcripts by fold-change and plotted the expression levels of these genes and their receptors in the skin and DRG bulk transcriptomic data at 0 h, 6 h, and ≥8 h time points (Table 2). We were particularly interested in molecules that were both highly induced in damaged tissue and had receptors that were strongly expressed in the DRG. Genes coding for cytokines and signaling molecules including oncostatin M (*OSM*), leukemia inhibitory factor (*LIF*), and prostaglandin-endoperoxide synthase 2 (*PTGS2*, also known as cyclooxygenase 2) were notably induced by incision and had receptors that were prominently expressed in DRG neurons (Table 2). Upon surgical incision, *OSM* and *OSMR* were induced roughly thirty-fold (0.2 sFPKM to 6.3 sFPKM) and four-fold (15.3 sFPKM to 57.2 sFPKM) in the skin when comparing 0 h to closure of the wound, respectively. In the DRG, *OSMR* is strongly expressed at 74.3 sFPKM, indicating that a complement of receptors is potentially available for tissue-to-nerve signaling. *LIF* was another cytokine that was highly induced by surgical incision and expressed at 7.8 sFPKM at 0 h and 37.5 sFPKM at the time of closure (380.77% increase). While its receptor *LIFR* was not induced in the skin, it was well expressed in DRG neurons at 152.1 sFPKM. The lipid biosynthetic enzyme, COX-2 (the inducible form of cyclooxygenase, encoded by *PTGS2*) was induced almost 20-fold from baseline, consistent with literature on secretion of prostaglandins at sites of tissue damage and their roles in mediating inflammation and immune cell recruitment^30,31^. Of the different receptors that products of COX-2 can bind to, the EP3 receptor (encoded by *PTGER3*) was prominently expressed in the DRG at 28.5 sFPKM. Note that COX-2 is responsible for the formation of diverse end products, although we focused on prostaglandin E2, which is one major product of the encoded enzyme with a strong salience to pain and/or inflammation. Top genes in our dataset, such as *CSF3* and *IL6* and genes encoding their respective receptors, *CSF3R* and *IL6R*, were highly expressed in the DRG transcript population, with *IL6R* being extensively described in DRG neurons in animal models of pain and nerve injury^32^. The quantification of these receptors in DRG neurons emphasizes the ability of tissue sequencing to identify potential molecular targets involved in pain signaling pathways.

**Table 2:** Expression values for significant secreted factors in skin, and their receptors in skin and DRG

Skin sFPKMs for secreted factors and receptors								
Gene	0 h	6 h	≥8 h	Receptor	0 h	6 h	≥8 h	DRG^a^
*CSF3*	0.0	93.3	58.7	*CSF3R*	2.9	46.8	35.8	4.8
*IL6*	0.4	388.1	352.0	*IL6R*	12.4	34.4	38.4	5.3
*--*	--	--	--	*IL6ST*	67.4	80.3	92.8	205.7
*IL8*	0.2	103.5	119.9	*CXCR1*	0.7	26.6	17.8	ND
*--*	--	--	--	*CXCR2*	3.8	30.7	20.8	0.1
*IL20*	0.0	9.9	17.9	*IL20RA*	21.4	16.8	13.8	0.3
*--*	--	--	--	*IL20RB*	115.6	54.4	41.4	0.8
*CXCL2*	0.9	178.7	158.4	*CXCR2*	3.8	30.7	20.8	0.1
*CXCL3*	0.4	50.9	47.4	*CXCR2*	3.8	30.7	20.8	0.1
*CXCL1*	1.2	62.3	62.0	*CXCR2*	3.8	30.7	20.8	0.1
*OSM*	0.2	7.7	6.3	*OSMR*	15.3	39.2	57.2	74.3
*HAMP*	0.2	5.5	11.4	*SLC40A1*	28.2	11.8	9.4	27.6
*PGLYRP1*	0.2	4.6	3.5	*TREM1*	0.3	10.4	10.0	0.1
*IL1B*	1.2	16.8	7.7	*IL1R1*	40.5	69.6	95.3	8.2
*PTGS2*	0.9	22.3	23.2	*PTGER1*	0.2	0.3	0.2	0.1
*--*	--	--	--	*PTGER2*	3.6	4.3	4.2	3.2
*--*	--	--	--	*PTGER3*	16.3	12.1	12.5	28.5
*--*	--	--	--	*PTGER4*	7.6	15.5	14.7	7.6
*CCL2*	37.5	772.6	749.1	*CCR2*	2.4	0.8	0.6	1.0
*--*	--	--		*CCR4*	1.4	1.3	1.4	ND
*AREG*	1.5	42.7	48.9	*EGFR*	13.1	16.3	16.4	8.3
*HBEGF*	5.8	94.1	87.7	*EGFR*	13.1	16.3	16.4	8.3
*--*	--	--	--	*ERBB4*	0.8	0.9	0.8	1.3
*EPGN*	0.4	4.6	6.9	*EGFR*	13.1	16.3	16.4	8.3
*ADCYAP1*	0.4	2.6	3.2	*ADCYAP1R1*	2.7	2.2	1.9	1.1
*CCN2*	51.2	246.4	253.1	*LDLR*	14.8	129.6	147.5	21.6
*--*	--	--	--	*NTRK1*	0.1	0.1	0.1	49.2
*--*	--	--	--	*ITGAV*	43.8	38.3	46.0	102.5
*--*	--	--	--	*ITGB3*	3.3	4.5	5.8	2.6
*--*	--	--	--	*ITGB5*	97.6	81.4	74.8	8.7
*--*	--	--	--	*FGFR2*	42.3	27.7	22.0	0.8
*CYR61*	31.9	256.0	209.7	*ITGAV*	43.8	38.3	46.0	102.5
*--*	--	--	--	*ITGB3*	3.3	4.5	5.8	2.6
*--*	--	--	--	*ITGB5*	97.6	81.4	74.8	8.7
*CXCL5*	0.0	1.6	2.3	*CXCR2*	3.8	30.7	20.8	0.1
*IL1B*	1.2	16.8	7.7	*IL1R1*	40.5	69.6	95.3	8.2
*--*	--	--	--	*IL-1R2*	25.0	48.5	59.5	0.9
*CCL8*	1.1	10.9	6.9	*CCR1*	2.8	7.4	5.9	5.8
*--*	--	--	--	*CCR2*	2.4	0.8	0.6	1.0
*--*	--	--	--	*CCR3*	0.2	0.3	0.2	ND
*--*	--	--	--	*CCR5*	2.1	0.8	0.6	1.8
*CCL20*	2.4	7.3	16.8	*CCR6*	1.9	1.5	1.7	0.6
*CCL3*	1.1	6.2	4.7	*CCR1*	2.8	7.4	5.9	5.8
*--*	--	--	--	*CCR4*	1.4	1.3	1.4	0.1
*--*	--	--	--	*CCR5*	2.1	0.8	0.6	1.8
*--*	--	--	--	*CCR9*	0.0	0.0	0.0	0.1
*LIF*	7.8	29.9	37.5	*LIFR*	25.3	22.0	28.7	152.1
*CCL4L2*	0.7	2.4	1.2	*CCR1*	2.8	7.4	5.9	5.8
*--*	--	--	--	*CCR5*	2.1	0.8	0.6	1.8
*TNFAIP6*	7.0	50.8	63.5	*CCR5*	2.1	0.8	0.6	1.8
*--*	--	--	--	*CCR7*	1.6	2.7	2.4	0.1
*--*	--	--	--	*CXCR4*	9.3	21.0	18.9	12.4
*PROK2*	0.1	13.4	11.8	*PROKR2*	0.0	0.0	0.0	0.6
*TFPI2*	0.7	4.3	6.0	*ITGAV*	43.8	38.3	46.0	102.5
*HP*	0.3	3.7	4.1	*CD163*	18.4	22.3	24.6	104.5
*LBP*	0.1	0.9	1.0	*CD14*	51.0	76.3	64.7	42.0
*ADM*	21.5	62.7	80.2	*CALCRL*	6.6	6.4	8.4	11.4
*--*	--	--	--	*RAMP2*	66.1	53.0	44.4	3.9
*--*	--	--	--	*RAMP3*	29.9	21.9	23.7	17.5
*TAC1*	0.9	1.5	3.0	*TACR1*	6.6	4.3	4.1	1.2

^a^DRG sFPKMs from human DRGs for secretome receptors.

**Fig. 7:**
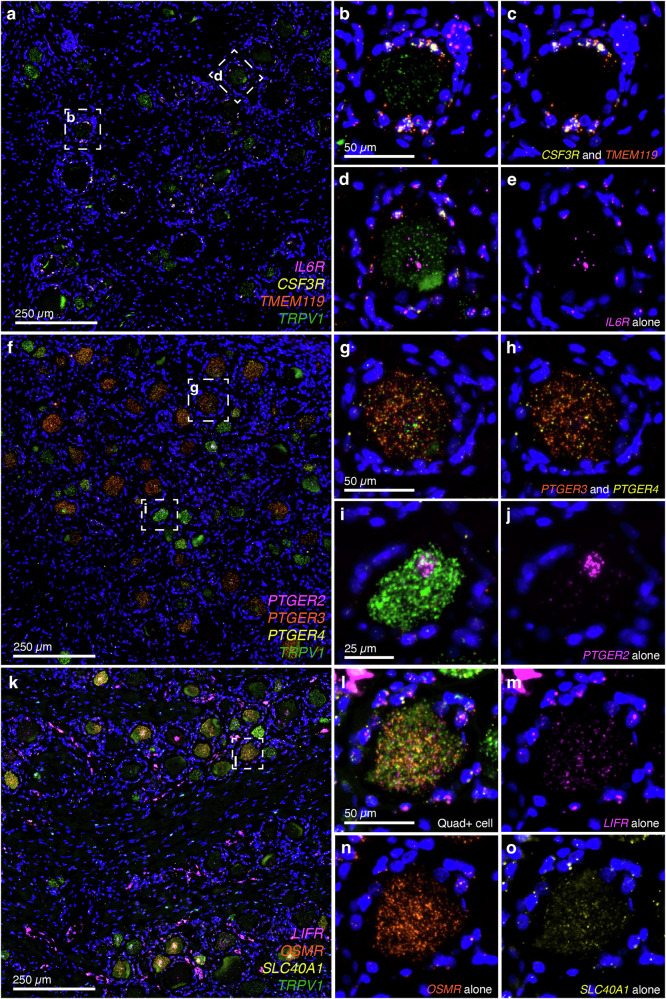
Validation of neuronal expression of receptor genes for incision-induced signaling molecules. Long-range signaling of secreted molecules from injured skin to dorsal root ganglion sensory neurons requires either long-range signaling of secretory factors via blood or signaling through neuronal axons in the DRG. To investigate whether neuronal axon-mediated interactions are possible for these induced signaling factors, we interrogated neuronal expression of these receptor genes. In **a**–**e**, we examined *IL6R*, *CSF3R*, *TMEM119*, and *TRPV1*. **a** A representative field is shown for the 4-plex investigation. **b** *TRPV1* is a neuronal marker gene for nociceptive neurons and was mainly found inside neuronal perikarya. A representative image of a dorsal root ganglion neuron that is positive for *TRPV1*. The gene encoding the colony-stimulating factor 3 receptor, *CSF3R*, is not expressed neuronally, but rather in macrophages, alongside *TMEM119*, which is also found in DRG macrophages^26^. **c** This is more apparent in the 2-channel image showing *CSF3R* and *TMEM119* alone. **d** *IL6R* was expressed in neuronal perikarya to a lesser degree but was highly coexpressed with *TRPV1*. **e** A single-channel image of *IL6R* alone shows that it is rarely coexpressed with macrophages but is expressed neuronally. In **f**–**j**, we examined *PTGER2, PTGER3, PTGER4*, and *TRPV1*. **f** A representative field of the 4-plex investigation is shown. **g** *PTGER3* and *PTGER4* are highly expressed in low-intensity *TRPV1*+ neurons. **h** A 2-channel image of *PTGER3* and *PTGER4*. **i** *PTGER2* is lowly expressed in neuronal perikarya expressing high *TRPV1*. **j** A single-channel image for *PTGER2* expression. In **k**–**o**, we examined *LIFR, OSMR, SLC40A1*, and *TRPV1*. **k** A representative field is shown for the 4-plex investigation. All markers of interest are found in neuronal perikarya, with *LIFR* additionally expressed in fibroblasts and endothelial cells (consistent with a detailed report using single-cell transcriptomics in human skin^34^). **l** A representative image of a dorsal root ganglion neuron that is positive for all four markers. **m**–**o** Single image channels for *LIFR*, *OSMR*, and *SLC40A1* are shown, respectively.

One major limitation of this approach is the assumption of neuronal expression from whole tissue RNA-Seq of human DRGs, which does not distinguish neuronal subtypes and does not discriminate between expression in neuronal and non-neuronal support cells in the DRG. To resolve this issue, we performed highly sensitive in situ hybridization for the genes of interest based on the significance of the induced ligand after surgery (in injured skin tissue) and the expression of the receptor in DRG tissue. We investigated three members of the IL6 cytokine family, *IL6*, *LIF*, and *CSF3*, and their major cognate receptors in human DRG tissue (Fig. 7a–e, k–o). Of these, *IL6R* was sparsely detected in DRG neurons, with broad expression throughout the tissue. The low level and sparseness of expression were striking, given the strong evidence from animal models of IL6 involvement in DRG signaling^32^, although IL6 may be additionally induced after insults such as nerve injury^33^. The macrophage receptor *CSF3R* was mainly located in DRG macrophages, as indicated by co-staining with *TMEM119* and the presence in small sporadically identified cells consistent with a macrophage staining pattern^26^. By contrast, *LIFR* was strongly expressed in a subset of neurons, indicating that *LIF* production locally by incision of the skin can directly stimulate neurons. Notably, *LIFR* was also broadly expressed in many other cell types, consistent with previous investigations^34^. Among prostaglandin receptors, we prescreened by baseline expression and determined that *PTGER2*, *PTGER3*, and *PTGER4* are expressed in human DRG^12,14,27^. Each of these receptors was identified in subpopulations of neurons, indicating that these are also capable of mediating long-range signaling (Fig. 7f-j). Finally, we investigated the expression of ferroportin (*SLC40A1*), which is responsible for transporting iron and interacts with the secreted factor hepcidin, which is associated with damaged blood cells (Supplementary Note D2). Ferroportin was identified on DRG neurons, indicating that neuronal sensory tissue damage may potentially, in part, be modulated through the hepcidin-ferroportin signaling pathway. In summary, these interactive pathways between damaged tissue and local DRG neuronal nerve terminals present a mechanism for mediating direct signaling from tissue damage to sensory afferents.

## Discussion

The present study identifies the major molecular transduction events in human skin after acute tissue injury from surgical interventions, expanding previous work in animal models and human tissue investigations^5,35–37^. Critically, we establish a simple, minimally invasive skin collection procedure with direct relevance to human health and disease. Additionally, our study points to a new appreciation for structures such as the hair follicle and sweat gland in response to incision, which showed evidence of robust activation by wounding in human incision. The identification of these structures in humans builds on animal work where they are less prominent or absent, and further builds on existing human work using proteomics to understand responses to incision in healthy volunteers^37^. We leveraged the anatomic information from large tissue slabs collected from the wound edge to expand on previous work using small circular biopsies, which would not fully capture large anatomical structures such as hair follicles (Supplementary Fig. 20 and Supplementary Note D4). The clinical relevance of this work is underscored by the substantial pain experienced by patients following surgery, with pain scores frequently falling within the moderate to severe range despite the administration of opioids and other pain medications (Fig. 1). This finding is particularly concerning given that opioids, while effective, carry a significant risk of long-term dependency. Approximately 6% of opioid-naive patients undergoing surgery develop long-term opioid use^38^, a phenomenon that appears to be largely independent of the severity of their postsurgical pain^39^. Therefore, this work connects back to a pressing long-term goal of developing opioid alternatives by revealing and prioritizing receptor–ligand pairs targetable for future analgesic strategies.

Responses to wounding can be divided into several major categories, with the most prominent being acute inflammatory response, host defense and pathogen recognition (or “defensive priming”), and early responses to initiate tissue remodeling and initiate repair^5,8,40^. Proinflammatory cytokines such as IL6, IL8, IL1B, OSM, LIF, and TNFAIP6 are rapidly induced, driving the recruitment and activation of immune cells, particularly neutrophils. The predominant immune cell appearing in our study at the acute time points was neutrophils, which is consistent with previous studies positing the importance of these cells in early responses to injury^5,8^. Accordingly, neutrophils are known to rapidly infiltrate wound sites and release a range of pro-inflammatory mediators, including cytokines and reactive oxygen species that can sensitize peripheral nociceptors and potentially contribute to acute pain hypersensitivity. However, the role of neutrophils in pain and wound resolution is increasingly recognized as complex and context-dependent^41,42^. Additionally, recent work has shown that neutrophil activation is associated with the resolution of inflammation and acute pain, and suppression of this response can lead to prolonged pain^41^. While we observe robust early neutrophil recruitment in skin tissue following surgical injury and clear transcriptional evidence of inflammatory mediator release, it is difficult to draw definitive conclusions about the contribution of these cells to acute postsurgical pain. Other chemokines such as CCL2, CXCL1 and CXCL2 also amplify these processes, participating in immune recruitment, which is known to occur at later time points not captured by the present study^5,8^. Genes involved in recognition of bacterial and viral proteins, such as LBP and APOBEC3A, as well as hepcidin, which sequesters iron, limiting availability to pathogens, are canonical hallmarks of these processes.

The most significant, strongly induced set of genes in the present study was the IL6 family of cytokines, particularly interleukin 6 itself (*IL6*). Additional members in the family that were strongly induced by surgery were oncostatin M (*OSM*) and leukemia inhibitory factor (*LIF*). An initial goal of the study was to identify specific targetable pathways relevant to either wound healing or pain mechanisms in order to be able to manipulate specific molecular pathways. However, at least among the IL6 family, the relationship between pain and wound healing is pleiotropic, regulating many cellular functions at once. Of these, *IL6* is one of the most studied and most significant molecules induced by wounding and has been extensively described as contributing to pain, immune cell recruitment, and wound healing^5,8,32^ (Supplementary Note D5). *IL6* has been extensively linked to pain in past studies (Supplementary Note D5), including functional studies in human DRG cultures^43^, suggesting this molecule is a major regulator of hyperalgesia. Serum IL6 levels increase in surgical patients and correlate with surgical stress^44^, where they are most likely caused by trauma at the wound edge, consistent with our finding that *IL6* expression continually increased throughout the surgical case with additional trauma. Other molecules such as *MT2A*^45^ and *OSM*^46^ have been proposed as modulators of wound healing, but similarly to IL6, OSM is also being investigated for its effects on pain and hyperalgesia^47^ with additional studies underway to investigate these effects in humans (Supplementary Notes D5 and D6).

Another major focus of the study was to identify secreted factors capable of long-range signaling via stimulation of the DRG axons innervating the dermal layers. An established example of long-range communication of this type is that between the lipid mediator prostaglandin E2 (PGE2), which can act on four receptors (encoded by *PTGER1-4*), some of which are expressed by DRG neurons, and cause pain when activated. Our multiplex anatomical investigation confirmed expression of these known nociceptive receptors in the nociceptive neuronal subpopulation. We identified two major populations: *TRPV1*+ nociceptive neurons containing *PTGER3* and *PTGER4* and those containing *TRPV1* and *PTGER2* alone (Supplementary Fig. 16f–j)^14^. After establishing this framing, we investigated the hits from our study for DRG neuronal receptors. In particular, *LIF* was a strong candidate for signaling to nociceptors because of the strong neuronal expression of its major receptor *LIFR*. Indeed, *LIFR* was broadly expressed at high levels by many DRG neurons in humans, including putative nociceptive populations. Conversely, despite its strong relevance to pain, IL6 receptor (*IL6R*) was not robustly detected on DRG neurons. This was surprising and raises questions about which IL6 receptor family member (or combination of receptors) may mediate human hyperalgesic processes thought to be caused by IL6. That is, one complexity in studying the skin-to-neuron signaling for IL6 family members is the crosstalk in their receptor systems, and the shared co-receptor GP130 (encoded by *IL6ST*), which forms the high-affinity receptor complex for several functional receptors, including LIFR^48,49^. In summary, while we have yet to investigate the functional implications of LIF-LIFR signaling after wounding, we establish that LIFR is a highly expressed neuronal receptor (arguably analogous to OSMR) while IL6R is not, which adds a quantitative context for future functional investigations. Additionally, while the finding of hepcidin induction at the wound edge is a canonical process, the finding that putative nociceptive neurons may be capable of surveying hepcidin release, since they express one of its binding partners, points towards a potential novel interaction between damaged skin and primary afferents that should be explored further.

In addition to these long-range signaling pathways between damaged tissue and neurons, many secreted signaling factor-receptor pairs were identified in the interactome that signal locally between skin structures, or within a single structure (Table 2 and Figs. 5, 6). OSM in particular, whose pathway was among the most significantly regulated in our study, was identified in many skin structures, including hair follicles and sweat glands. This indicates involvement of keratinocytes and potentially epidermal stem cells in this major pathway activated after tissue injury. Perhaps the most striking facet is the degree to which certain factors, such as *OSM*, apparently interact with many of these functions at once, underscoring the complexity of this system in a tissue injury context. Parenthetically, *OSM* has also been proposed as a mediator of itch, although these time points are too early to be consistent with itch, and tissue damage induces *OSM* expression much more strongly than pathological itch states (Supplementary Note D5 and Supplementary Fig. 21). Our data supports that OSM acts as a regulator of many of the synchronized functions necessary to respond to tissue injury by signaling to both local skin cells and neurons immediately after the acute events.

The study has several limitations. First among them is that the cohort (*N* = 12) does not allow for in-depth comparisons by age, underlying disease status, or other factors that may be important in the magnitude or identity of genes induced by surgical injury. Additionally, while the study extends our previous findings from the rat, each study is essentially independent due to multiple model and species differences in experimental approach, skin characteristics, healing mechanism and other factors. Nonetheless, we posit that demonstration of the most relevant genes in human incisions, in particular, is the most relevant data for validating mechanisms relevant to human wound responses and healing. Additionally, it will be important in the future to validate functional signaling and physiologic consequences of the skin-to-neuron interactome hypotheses to further understand the importance of these observations. Finally, one of the most critical questions—that of predicting long-term pain and outcomes based on gene expression—remains unaddressed. The genes in the present study form essentially a unified signature that does not vary across patient characteristics such as post-operative pain or sex as measured in this small cohort, but in the future, a skin or serum biomarker of such an outcome may be identified. This is despite reported differences in pain and inflammation according to sex in previous studies^50^. Pain in real surgical patients, such as in this study, is extremely complicated, as pain was managed postoperatively according to the standard of care, and their pain therefore represents a mix of complex factors^51^, including efficacy of the pain management strategy.

Many (if not all) of the molecules identified in the present study regulate wound healing, inflammation, and nociceptive sensitivity concurrently. This pleiotropy is also particularly evident in IL6 family members. Importantly, while the pleiotropy can make it difficult to target specific disease processes, we propose that this may be a mechanism that is particularly relevant in poorly healing wounds. For example, some evidence exists that IL6 family members remain elevated in poorly healing wounds, which likely continually sensitizes the nociceptive afferents despite failing to mobilize the healing response. While further investigations are needed to refine our understanding of the complex signaling pathways involved in the response to tissue injury and their implications for pain and wound healing, our study provides a critical framework for future studies aimed at elucidating these mechanisms in greater detail.

## Methods

### Study approval and inclusion criteria for human subjects

The study was approved by the Institutional Review Board of The National Institutes of Health, Clinical Center (Bethesda, Maryland; 20-CC-0031) and the protocol was registered at ClinicalTrials.gov (A single cohort study collecting interval timed incisional epidermal and dermal tissue samples during surgical procedures to profile temporal response of tissue after noxious stimuli. NCT04224870) on January 13, 2020. Informed consent was obtained from all participants prior to enrollment in the study, including consent to deposition of data into sequencing databases, as per NIH policy. Additionally, informed consent was obtained for coded data to be stored and used for future research, including the sharing of coded data with other researchers. This manuscript follows the guidelines of the Strengthening the Reporting of Observational Studies in Epidemiology (STROBE) statement. The study planned to recruit 12 patients according to a statistical estimate performed during protocol drafting (which was met by study completion). Inclusion criteria were English-speaking adults (age 18 or over). Patients were enrolled in this secondary protocol based on their recruitment to a primary surgical protocol with a scheduled surgical procedure expected to last longer than 4 h. For all participants, cutaneous incisional tissue removal was determined by the surgeon not to compromise patient safety or wound closure. Exclusion criteria included known skin abnormalities at the incisional site, such as uncontrolled dermatologic conditions, burns, infections, hematomas, and contusions. Additionally, patients with pre-existing scar tissue or a history of radiation treatment at the incision were excluded. More broadly, participants with skin conditions that compromise epidermal and dermal samples in the area of incision were excluded. All ethical regulations relevant to human research participants were followed.

In total, 12 patients were enrolled in the study (Table 1), including 5 male and 7 female subjects. These subjects were predominantly White (10/12) with one Black and one other ethnicity. 1 subject was Hispanic. One surgery met the initial inclusion criteria but was completed ahead of schedule (a 2 h surgery with samples collected at 0 h, 1 h, and 2 h). All other surgeries were ≥4 h. The longest surgery was ~13.5 h. 11/12 of the surgical procedures were American Society of Anesthesiologists (ASA) class III (defined as a patient with severe systemic disease), with 1 surgery at ASA class IV (defined as a patient with severe systemic disease that is a constant threat to life). Four surgeries included the infusion of chemotherapeutic agents through hyperthermic intraperitoneal chemotherapy. The type of surgery and underlying medical history of subjects varied, although the region of skin sampled was thoracic or abdominal, and collected as a protocol secondary to a primary disease-modifying intervention.

Dorsal root ganglion tissues were collected from four healthy donors. Tissues were provided by AnaBios Corporation (San Diego, CA) and processed as previously described^25,26,28^. Briefly, these samples were formalin fixed by emersion from flash frozen tissue, and paraffin embedded for sectioning at 6 µm. Detailed donor information is reported in Supplementary Table 6.

### Pain assessments

Pain scores were assessed using the Brief Pain Inventory^10^ and McGill Short form^11^, which were accessed through IMMPACT, and used according to standard procedures, as described previously^52^. Briefly, pain questionnaires were conducted on the day before the surgery at a screening visit, as well as two time points (24 h and 48 h) after surgery. In general, pain was not present before surgery, but subjects were asked to give a description of their pain score for the region where the surgery was planned. One patient was unable to complete questionnaires post-surgery. Statistical testing was performed in Prism 10 (v10.2.3, GraphPad, Boston MA; see results). All hypothesis testing was two-tailed and *p* < 0.05 was defined as significant.

### Study approval for animal procedures

Rat experiments were performed under an approved animal protocol at the National Institutes of Health, Clinical Center (DPM 16-02). Further analyses of stains of large tissue scans were performed based on a previously published investigation of rat hind paw incision^5^. This experiment consisted of *N* = 5–6 male rats per time point, examined at the following time points after hind paw incision to simulate surgical incisional pain and tissue injury: 0 h, 1 h, 6 h, 1 day, 3 days, 6 days, 12 days. We have complied with all relevant ethical regulations for animal use.

### Tissue processing

At selected surgical time points, skin samples were removed by the surgeon at the incisional edge according to the diagram in Fig. 1g. Samples were taken sequentially such that the same area was not sampled more than once, as such a collection would have a variable distance from the initial incisional edge (i.e., incorporating the incision performed at time zero). Additional effort was taken to avoid areas of prolonged retraction or tissue damage, otherwise not typical of the initial incision. Some additional factors, such as electrocautery, were not possible to avoid. Evidence that the samples were collected in a homogeneous fashion comes from the lack of variation between individuals at the same time point that was performed as part of the quality control metrics detailed below (see section on RNA-Seq analyses), as well as visual inspection of formalin fixed sections using H&E staining.

The overall size of each tissue sample was 24 mm in length by 2 mm in width, with sample depth including epidermis and dermis, and stopping before subcutaneous adipose. The first sample collected (time zero) was 26 mm long to provide a 2 mm sample to the pathology department. This tissue sample was immediately processed into three 8 mm long subdivisions. These three pieces were (1) stored in 10% neutral buffered formalin at room temperature, (2) stored in RNA-later at room temperature or (3) on dry ice. In the case of RNA-later stabilized tissues, additional cuts were introduced with a sharp scissor to increase surface area for faster stabilization. For dry ice frozen tissue, samples were further divided in half.

Samples were collected in a longitudinal fashion during the course of the surgery, with the first sample being taken at the time of the initial incision (time zero control). Subsequent samples were taken 1 hr, 2 h, 4 h, and 6 h after this initial tissue sample was taken. If the surgery lasted 8 h or longer, a sample was taken at closure (the longest at 12 h, 44 min after incision). For all time points after initial incision, there was an allowance of ±30 min to account for stages of the surgery that could not be interrupted, although in most cases the sample was taken close to the planned time point.

### Histopathology

Histological sections were fixed by immersion in 10% neutral buffered formalin for 16–24 h starting at the time of collection. Subsequently, fixation was slowed by transfer to a 2.5% formalin solution and storage at 4 °C until embedding. Tissues were generally embedded as soon as possible (within 2–5 days). Preparation of paraffin blocks was performed at Histoserv Inc. (Germantown, MD). Fixed tissues were dehydrated through graded alcohols, cleared with xylene, then infiltrated with paraffin before embedding in paraffin blocks. The resulting blocks were cut on a microtome at 5 µm. Cut sections were stored at −80 °C until use.

For histological stains, slides were deparaffinized in xylene and hydrated in graded alcohols, then water. Staining was performed using Carazzi’s hematoxylin and eosin-phloxine solution. For Masson’s staining, Bouin’s solution was used as a mordant. Subsequently, slides were rinsed well and transferred to Weigert’s hematoxylin, then placed in Biebrich scarlet-acid fuchsin, followed by phosphotungstic phosphomolybdic acid. Finally, slides were stained with aniline blue. For both methods of histological staining, slides were dehydrated by graded alcohols up to xylene, and coverslipping was performed using Permount (Fisher Scientific, Hampton, NH).

### RNA extraction, library preparation, and next-generation sequencing

RNA was extracted using the Invitrogen PureLink™ RNA mini kit (Thermo Fisher Scientific, Waltham, MA) and QIAzol (Qiagen, Venlo, Netherlands) following the manufacturer’s instructions for phenol-chloroform column chromatography. The full protocol is found on protocols.io (10.17504/protocols.io.j8nlk81w1l5r/v1).

Poly-A selected stranded mRNA libraries were constructed from 1 µg total using NEBNext Poly(A) mRNA Magnetic Isolation Module (NEB #E7490) and NEBNext Ultra II Directional RNA Library Prep for Illumina with Sample Purification Beads (NEB #E7765, New England Biolabs, Ipswich, MA). Amplification was performed using 8 cycles to minimize the risk of over-amplification. Unique dual-indexed barcode adapters were applied to each library. Libraries were pooled in an equimolar ratio for sequencing. The pooled libraries were sequenced on an S4 flow cell on a NovaSeq 6000 (Illumina, San Diego, CA) using v1.5 chemistry to achieve a minimum of 60 million 150 base read pairs. The data was processed using RTA version 3.4.4.

### RNA-Seq alignment and quantification

Alignment using a 2019 build of the MAGIC RNA aligner (ftp://ftp.ncbi.nlm.nih.gov/repository/acedb/Software/Magic; magic.2019_04_19, LINUX_4_OPT) and a human genome build with additional genomic targets added for viruses and bacteria as described previously^12^. Genomic target files are available through the NCBI public server. Raw counts and sFPKM values are generated within this pipeline, as well as a differential score, which gives false-discovery rate corrected significance values for each comparison.

### Gene clustering analysis

Gene changes were clustered according to temporal patterns using the heatmap function in R (v4.2.3; Fig. 2a)^5,8^. This separates genes by patterns of expression over time, and has previously been shown to be able to separate gene expression patterns over time. However, in this dataset, the major separation was only “up” vs. “down”, implying a simplified expression pattern. This heatmap was created using k-means clustering (*k* = 4), with the primary difference being two groups. Expression ratio was calculated for the average at each time point (average/max value) to convert to a normalized scale with values between 0 and 1. Genes from each of the four main clusters were plotted to show the overall pattern within each cluster, as well as the number of genes in each pattern. The average pattern (±error) is shown to summarize the progression of gene measurements over the time course analysis (Fig. 2b–e).

### Pathway and protein-protein interaction network

Groups of related genes changing at the same time point can often be functionally related by signaling within the same pathway. To identify pathways associated with significant genes, significant gene lists at each time point were analyzed for enrichment using the Enrichr tool^53^, which is a simple web-based tool that accepts the names of genes and predicts biological function based on gene lists^40^. Specifically, increasing and decreasing genes were imported as separate gene lists to Enrichr, analyzed using the BioPlanet 2019 pathway tool, and significant terms were exported. These data are plotted as a heatmap with each color representing the significance score (−log *p* value) output by this tool, with a score of 2.0 (corresponding to *p* < 0.01) considered as significant. Interactions between significant genes were also tested using the String db tool to examine clusters of interacting proteins encoded by significant genes. String analysis was performed specifically on the top 100 genes in the dataset (Supplementary Fig. 7c). This is also a web-based tool that accepts gene lists as inputs, and default parameters were used, according to the developers’ protocols^40^.

### Leukocyte heatmaps and imsig analysis

These analyses were performed as described previously^5,8^. Selections of immune genes enriched in various immune cell types were plotted as heatmaps according to lists of enriched markers. General lists were mined from Biocompare (see “A guide to neutrophil Markers”, Donne Estipona, 2021, https://www.biocompare.com/Editorial-Articles/577944-A-Guide-to-Neutrophil-Markers/) and then refined by reviewing the cited references and general literature (as described in Supplementary Note C4). Alongside these analyses, imsig (v1.1.3) was used (default settings) according to the provided vignettes in R (v4.2.3). This package estimates immune cell abundance and limited analysis of pathways in bulk RNA-Seq datasets using cell-type-specific and/or enriched markers.

### Multiplex in situ hybridization in human intraoperative skin samples and human DRGs

Multiplex in situ hybridization of human skin samples was initially performed by Advanced Cell Diagnostics (Newark, CA) using the RNAscope® LS Multiplex Reagent Kit (Cat. No. 322800) following standard procedures. The following standard protocols were used: Epitope Retrieval 2 (15 min at 95 °C); Protease III (15 min at 40 °C). Detailed information about the in situ hybridization probe sets is found in Supplementary Table 7. Detection was performed using the following dyes and filter sets: Opal 520 (1:500), FITC filters; Opal 570 (1:1500), SpOrange filters; Opal 620 (1:1500), Cy5.5 filters; Polaris 780 (1:125), Cy7 filters. Image acquisition for fluorescence and brightfield images was performed using a Pannoramic 250 scanner (3DHISTECH, Ramsey, NJ). Subsequent staining procedures and replication experiments were performed at the National Institutes of Health as described below.

Multiplex in situ hybridization of human DRG and skin samples was also performed at the National Institutes of Health as described previously using the RNAScope® Version 2 kit (Advanced Cell Diagnostics) standard procedures except where noted. In these experiments, prior to staining procedures, samples were photobleached using a prototype white LED to remove endogenous autofluorescence^54^. This procedure consisted of exposing formalin-fixed paraffin-embedded sections to bright white LED lights for 3–4 days. Human DRGs were provided by AnaBios Corporation (San Diego, CA) and visualized using Opal™ Reagent Systems (Akoya, Marlborough, MA). Target retrieval was performed for ~20 min at 100 °C. Digestion was performed using the Protease Plus reagent. Image capture of hybridized stained slides was performed using an Axio Imager.Z2 scanning fluorescence microscope (Zeiss, Oberkochen, Germany)^25,26,55,56^. For final visualizations of both methods, single-channel microscopy images captured at each emission wavelength were overlaid to generate multi-colored composites in Adobe Photoshop (San Jose, CA). Representative images are enhanced for visibility. Multiplex in situ hybridization in rat tissue was performed as part of a previous publication^5^, with new visualizations performed on the raw data used to create previously published microscopic images. These images are whole tissue scans and could not be presented in full in the previous publication. Staining and image collection were performed using the same procedures as for human skin and DRG, and conducted using similar equipment in the NINDS Flow Cytometry and Imaging Core Facility.

For quantitative cell counting in human DRG, cells were identified as expressing or not expressing each of the four labels by manual inspection, and were tallied, and visualized following methods similar to previous reports^24,25,56^. Briefly, the signal was localized to neurons based on brightfield and DAPI in multichannel fluorescence imaging. Visualizations of overlap were created using the upSetR package in R, and colored in Adobe Illustrator for clarity (colors matching the fluorophores).

### Selection and visualization of significantly differential secreted genes

In order to highlight the most differential secreted genes, the list of 1163 total significantly increasing DEGs in the dataset was assigned a major cellular compartment by mapping to existing databases of gene function and location, following similar procedures as described previously^5,8,56^. Briefly, the gene list was mapped to the International Union of Basic and Clinical Pharmacology (IUPHAR) database to capture functional categories relevant to pharmacology, including transporters, G-protein-coupled receptors, and ion channels. Genes that were not identified in the IUPHAR database^57^ were queried against additional databases as follows, and assigned more general categories. Additional information about cellular compartments was gathered from the COMPARTMENTS database^58^, GeneCards^59^, and UniProt^60^. In cases where genes were found in multiple subcellular compartments, secreted genes were generally considered non-membrane-bound soluble proteins that are primarily secreted. The top 50 significant genes in the “secretome” of the human skin incision were used to construct a heatmap, with values in each cell representing expression level at that time point divided by maximum expression value, such that the maximum value in each row is 1 (Fig. 3c). IUPHAR categories (with minor manual revisions) were also used to extract g-protein coupled receptors and catalytic receptors in Supplementary Fig. 9.

The secretome described above was subsequently used to generate an “interactome” following the general strategy outlined in a previous publication examining inflammatory signaling to human DRG^61^. A selection of genes in the secretome was assorted with their respective receptors in ligand–receptor signaling pairs and examined for expression differences and basal expression levels in human skin (pre-post incision) and human DRG, respectively. Ligand-receptor pairs were identified manually through a literature review.

### Immunohistochemical staining

Immunohistochemistry for Calprotectin, Metallothionein 2A (MT2A), and SRY-Box Transcription Factor 9 (SOX9) in the DRG and skin was performed in six tissue donors by Histoserv, Inc. Slides were deparaffinized and hydrated through graded alcohols to distilled water, followed by antigen retrieval at 70 °C for 40 min using pH 6 citrate buffer. Sections were then blocked with hydrogen peroxide and a blocking serum (5% BSA) and washed in distilled water. Next, the slides were incubated with Calprotectin (MA5-12213, Thermo Fisher Scientific, 1:1000), MT2A (PA5-102549, Thermo Fisher Scientific, 1:100) or SOX9 (#ab185230, Abcam, Cambridge, England, 1:200). Finally, the slides were developed using Fuchsin Red (Dako #K0625, Agilent, Santa Clara, CA), and counterstained with hematoxylin. All incubations were carried out at room temperature, and Tris-buffered saline with 0.1% Tween-20 was used as a washing buffer. The stain for Calprotectin (dimer of the proteins encoded by *S100A8* and *S100A9*) was also corroborated in a replication of the experiment with an MRP8 (encoded by *S100A8*) antibody (#ab201473, Abcam, 1:1000), providing highly similar results (Supplementary Fig. 21).

### Statistics and reproducibility

DEGs were determined using MAGIC following prespecified criteria in the IRB-approved clinical protocol prior to study start. This method applies a false discovery rate correction to a differential score based on the separation of expression values between groups^12,35^. Confirmatory and exploratory measures, such as microscopy-based validation of anatomical location, are presented as qualitative data. The basic tissue collection protocol was developed to recruit 12 patients based on previous experience with *N* = 6 animal subjects, with a conservative estimate for attrition and the ability to interrogate sex differences, if present. Additionally, the *N* = 12 group size was selected to attempt to capture variability (if present) that exceeded the variability in animal studies. Additionally, the time series measurement ensures each sample has its own time zero control for baseline comparisons.

### Reporting summary

Further information on research design is available in the Nature Portfolio Reporting Summary linked to this article.

## Supplementary information

Supplementary Information

Description of Additional Supplementary Files

Supplementary Data 1

Reporting Summary

## Data Availability

Sequencing data were deposited in public databases (Sequence Read Archive, BioProject# PRJNA1154260; dbGaP: phs003890) alongside sample-level patient-reported outcomes and data dictionaries. Large supplementary data tables (Supplementary Tables 1–5) are also available on figshare (10.6084/m9.figshare.27384174). Source data underlying all graphs in the manuscript can be found in Supplementary Data 1.
