# Supplementary material for: Longitudinal human transcriptomic and spatial gene profiling at the incisional edge during long surgical procedures
Source: Commun Biol. 2025 Dec 22;9:315. doi: 10.1038/s42003-025-09366-0 (PMC12936088; doi:10.1038/s42003-025-09366-0)
Supplement: Supplementary file 1 — Supplementary Information [file 42003_2025_9366_MOESM1_ESM.pdf]

Supplement to: Longitudinal human epidermal and dermal transcriptomic and spatial gene profiling at the incisional edge during long surgical procedures.

Matthew R. Sapio[1], Evelyn Li[1], Anthony F. Domenichiello[2,3], Taichi Goto[4], Dragan Maric[5], Allison P. Manalo[1], Tracy S. Williams[1], Saber Tadros[6], Andrew M. Blakely[7], Jeremy L. Davis[7], Jonathan M. Hernandez[7], David S. Schrump[8], Michael J. Iadarola[1], Andrew J Mannes[1*]

*To whom correspondence should be addressed: Department of Perioperative Medicine, Clinical Center, National Institutes of Health, Building 10, Room 2C744, 10 Center Drive, Bethesda, MD 20892-1510. amannes@cc.nih.gov

1. NIH, Clinical Center, Department of Perioperative Medicine, Bethesda, MD 20892, USA
2. Lipid Peroxidation Unit, Laboratory of Clinical Investigation, National Institute on Aging, National Institutes of Health (NIH), Baltimore, MD, 21224, USA
3. National Institutes of Health, National Institute of Neurological Disorders and Stroke, Office of Pain Policy and Planning, Bethesda, MD, 20892, USA
4. National Institutes of Health, National Institute of Nursing Research, Symptoms Biology Unit, Bethesda, MD, 20892, USA
5. National Institutes of Health, National Institute of Neurological Disorders and Stroke, Flow and Imaging Cytometry Core Facility, Bethesda, MD, 20892, USA
6. University of Nebraska Medical Center, Department of Pathology, Microbiology and Immunology, Omaha, NE, 68198, USA
7. Surgical Oncology Program, Center for Cancer Research, National Cancer Institute, Bethesda, MD, 20892, USA
8. Thoracic Surgery Branch, Center for Cancer Research, National Cancer Institute, Bethesda, MD, 20892, USA

Keywords: surgery, incision, epidermal, dermal, transcriptomics

Table of Contents

A. Supplementary Figures……………………………………………………………2
B. Supplementary Tables…………………………………………………………...32
C. Supplementary Notes…………………………………………………………....36
D. Supplementary Discussion……………………………………………………38
E. Supplementary References………………………………………………….. 43

Brief pain inventory measures

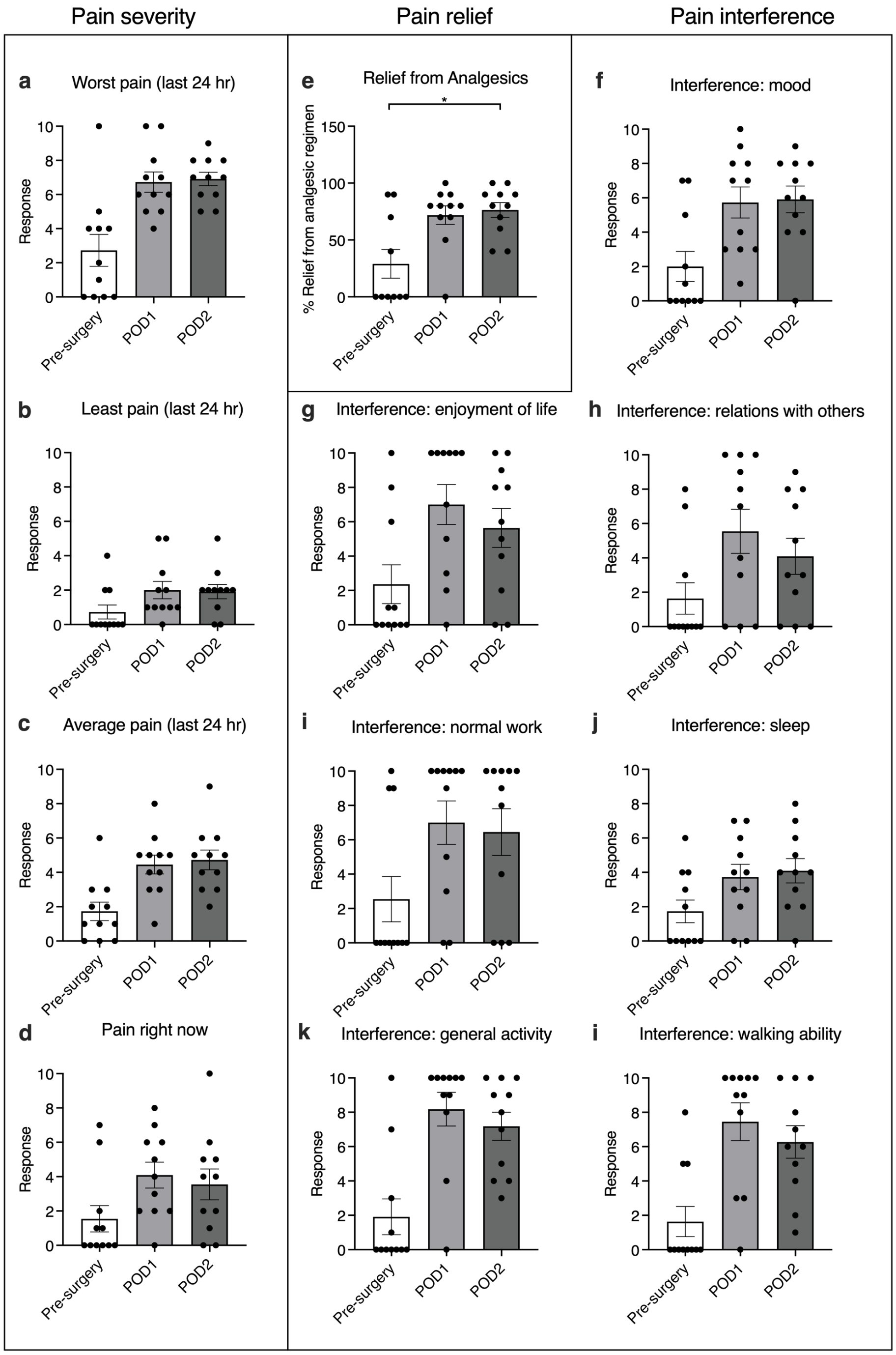

Supplementary Fig. 1. Individual items for Brief Pain Inventory. Pain severity and pain interference pre- and post-surgery were assessed using the Brief Pain Inventory (BPI). Generally, patients reported increased pain severity on postoperative days (POD) 1 and 2 compared to the preoperative period. Postoperative pain management involving analgesics significantly relieved pain severity. Patients also reported notable pain interference with functional outcomes including walking ability, sleep, general activity, and enjoyment of life.

McGill

Sensory questions

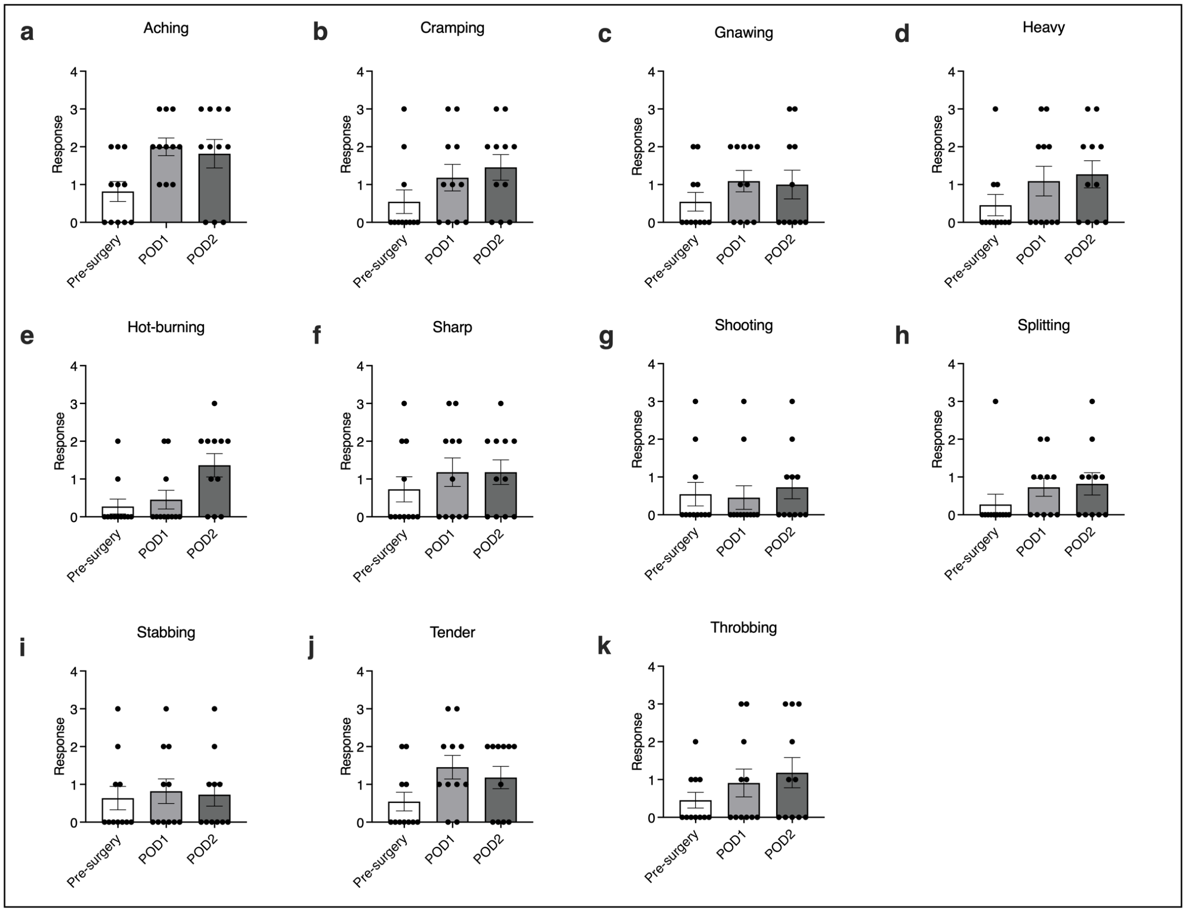

Affective questions

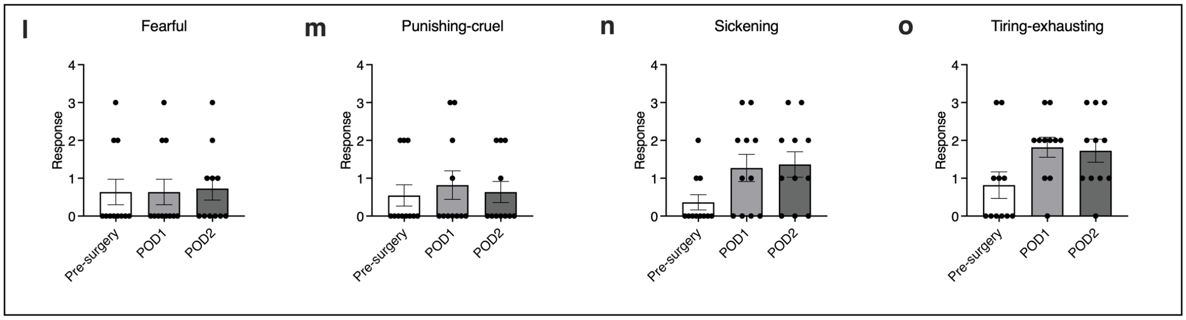

Supplementary Fig. 2. Individual items measured using the McGill Pain Questionnaire. Information on pain descriptors and modalities was collected with the McGill Pain Questionnaire. Aching and cramping pain and pain that was sickening and tiring-exhausting were the most common sensory and affective components, respectively, reported by patients in the postoperative setting. Composite scores of sensory and affective modalities also show a general increase before and after surgery.

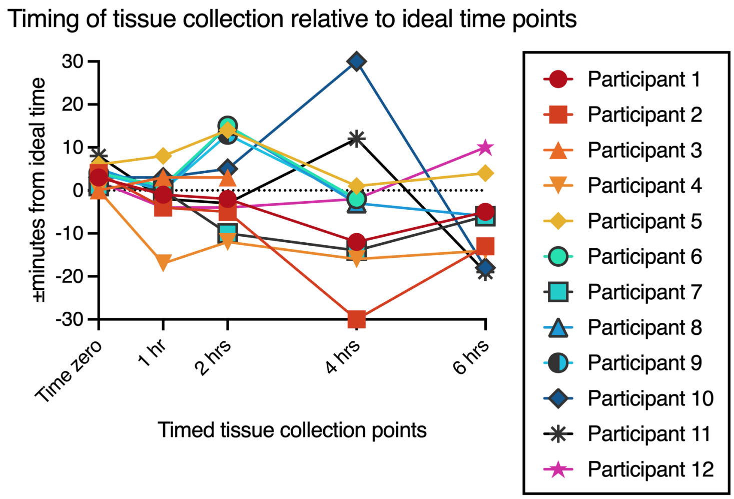

Supplementary Fig. 3. Actual vs. ideal times for tissue collection of each participant at each time point. Tissue collection was precisely timed, with the majority of samples collected within minutes of the ideal time. However, some exceptions were made if the surgical procedures did not allow for tissue collection at that time, or if it posed a safety concern. No sampling time point exceeded 30 minutes from the ideal timing.

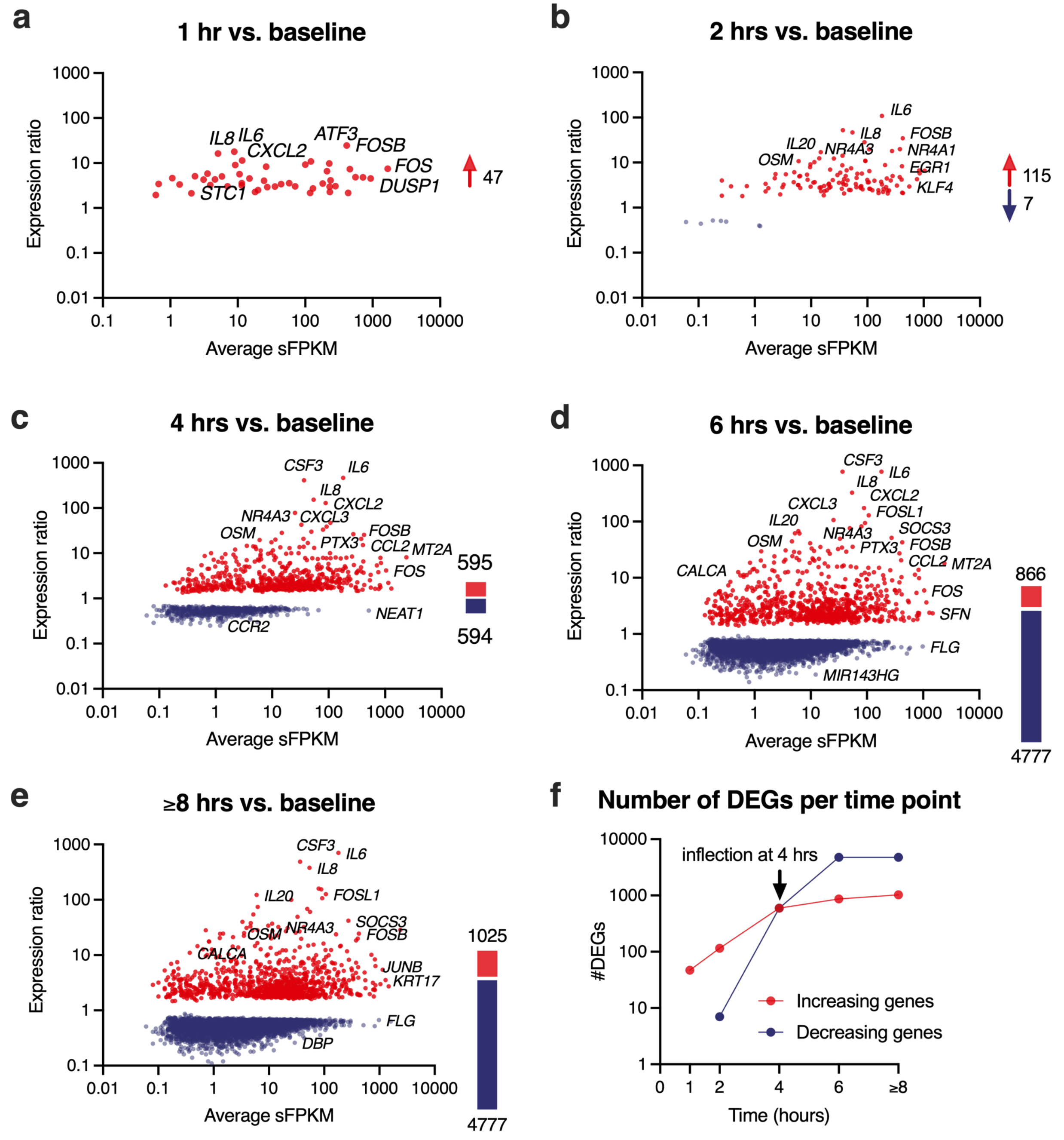

Supplementary Fig. 4. Expression ratio and expression level (in sFPKM) of significant genes from transcriptomics. To represent all of the significant genes and prioritize them for further study, all significant genes were shown for each time point relative to control. In each panel, the total number of increasing (red) and decreasing genes (blue) is represented in a bar plot to the right of the scatter graph. **a** At the 1 hr time point, 47 genes increased, including early induction of cytokines (such as *IL6* and *IL8*, which are among the most strongly induced genes at later time points), and rapid induction of transcription factors such as *FOS*, *FOSB* and *ATF3*. **b** At 2 hours after

incision, the majority of genes increase, and the total number increases to 115. **c** By 4 hours after incision, the balance of increasing and decreasing genes is approximately equal (595 increasing vs. 594 decreasing). **d, e** By the later time points, the number of significant genes is vastly greater, particularly among the decreasing genes (4777 genes at both time points.) **f** In describing the total number of significant genes over time, we observed an inflection point at about 4 hours, where the number of decreasing genes markedly increases, which continues to the end of the time course.

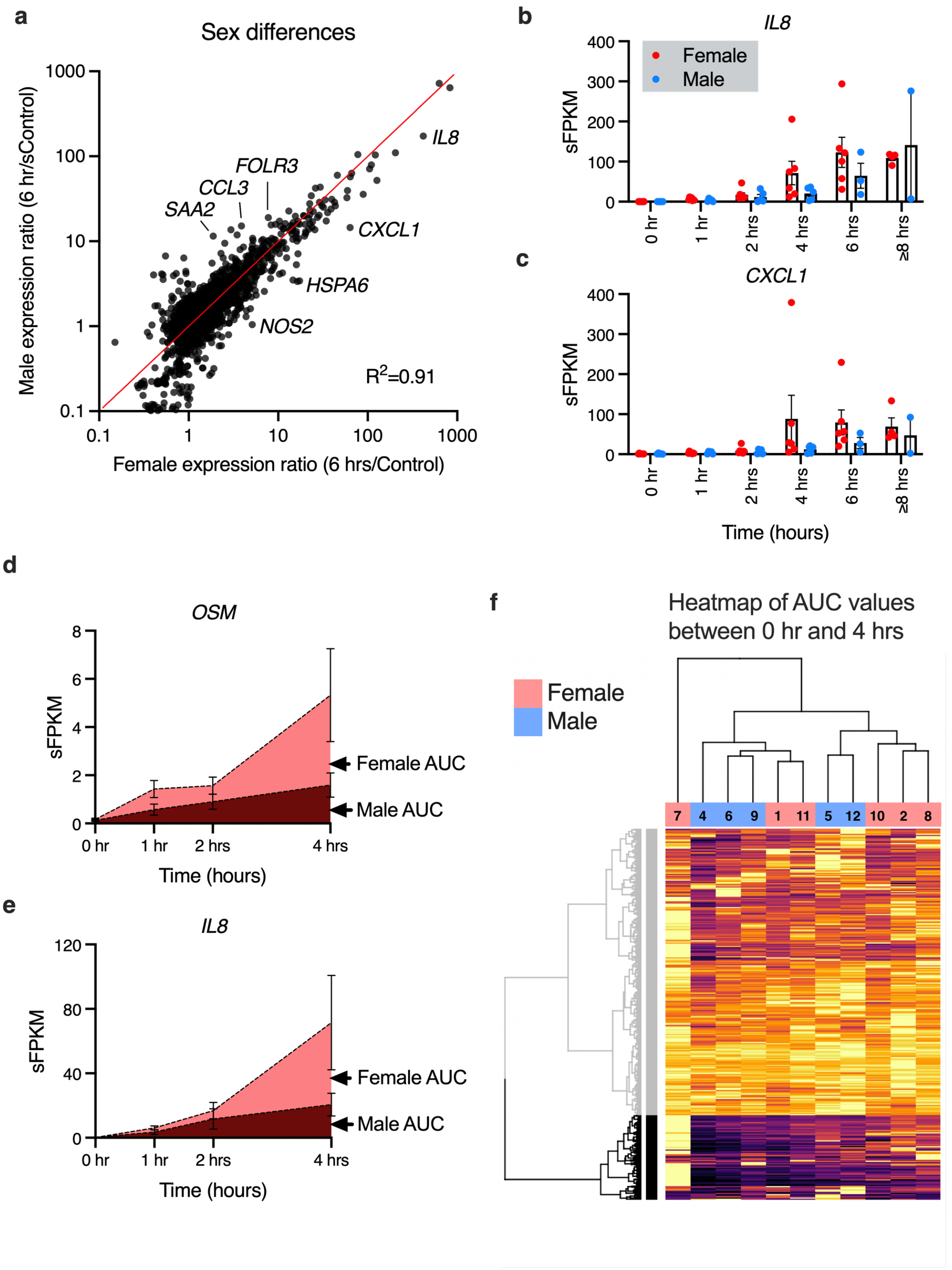

Supplementary Fig. 5. Investigation of sex differences and individual differences across human participants. a In order to investigate the impact of sex on the significant genes in the study, we correlated the expression ratio between control and 6 hours for male and female participants as a screen to select the most highly differential genes. Overall, these genes were highly correlated, and no genes were induced only in men or only in women (R^2 = 0.91, $p < 0.0001$, linear regression, Prism 10, GraphPad). **b** The results of this analysis showed that most genes were variable upon further

inspection, indicating they may be due to random variation between individuals and/or induction status rather than sex. Examples of genes with high differential expression ratios included Interleukin 8 (*IL8*), and **c** the chemokine (C-X-C motif) ligand 1 (*CXCL1*). Note the variance in the data, and impact of outlier points. **d** To further examine these effects, we calculated the area under the curve (AUC) using the trapezoidal method for all genes and examined significantly induced genes with the highest nominal differences in AUC values. Oncostatin M (*OSM*), and **e** Interleukin 8 (*IL8*) are shown as examples of genes large differences in AUC using this method. **f** However, as a quality control metric, we constructed a heatmap using the AUC values of the 1163 significantly increasing genes to see if sex predicted induction of these genes. In this analysis, sex was not predictive of responsiveness for most genes, indicating that genes with apparent large differences such as *OSM* and *IL8* may be random variance encountered by chance. The only notable difference was that patient 7, who is female, is apparently an outlier, particularly for the genes at the bottom of the plot, where this participant's skin samples show stronger induction at 4 hrs.

To perform formal statistics to address this question, the primary RNA-Seq pipeline (MAGIC) used throughout the study is not capable of performing linear modeling with interaction effects. Data from control and 4 hr time points (raw counts) were reanalyzed using DESEQ2 (R, version 4.4.0) to perform a formal statistic to examine the effect of sex on incision response (interaction effect). This was performed using the following formula:

```
design = ~ Sex + Condition + Sex:Condition
```

This analysis resulted in no significant genes after adjustment for multiple comparisons. The major conclusion of these analyses is that sex is not a major determinant of transcriptional response to surgical incision in this cohort.

PT1	0 hr	1 hr	2 hr	4 hr	6 hr
0 hr	67.31	51.23	45.11	28.15	-3.68
1 hr	71.15	73.45	67.58	56.71	26.59
2 hr	64.12	67.28	70.61	63.84	40.48
4 hr	44.6	49.46	61.26	67.8	72.49
6 hr	21.34	27.52	40.01	51.04	78.11
≥8 hr	14.5	14.23	27.56	36.61	63.49

PT2	0 hr	1 hr	2 hr	4 hr	6 hr	≥8 hr
0 hr	73.4	30.49	8.9	-27.07	-31.89	-35.69
1 hr	62.07	46.92	23.08	-5.19	-4.42	-16.85
2 hr	41.35	37.81	29.52	11.15	13.39	8.75
4 hr	-13.92	0.51	22.42	69.37	56.74	67.73
6 hr	-30.24	1.01	18.53	74.72	66.23	87.69
≥8 hr	-29.31	-9.34	6.49	61.02	45.64	81.62

PT3	0 hr	1 hr	2 hr
0 hr	69.99	44.72	43.45
1 hr	57.66	58.51	53.49
2 hr	45.1	49.87	55.02
4 hr	-0.91	13.81	14.93
6 hr	-11.69	9.2	6.7
≥8 hr	-14.47	0.72	1.22

PT4	0 hr	1 hr	2 hr	4 hr	6 hr	≥8 hr
0 hr	68.18	70.16	70.72	53.98	8.9	29.01
1 hr	57.18	67.69	68.95	67.17	25.42	41.4
2 hr	45.84	59.1	65.48	71.59	44.82	57.28
4 hr	17.7	19.79	22.61	57.29	67.42	66.21
6 hr	-7.1	-7.49	-4.62	24.24	55.43	48.18
≥8 hr	-3.02	-4.21	-2.51	22.78	49.58	49.91

PT5	0 hr	1 hr	2 hr	4 hr	6 hr	11 hr	13.5 hr
0 hr	67.03	55.79	46.43	39.81	25.21	20.83	15.41
1 hr	72.84	72.49	66.76	62	48.73	37.47	31.42
2 hr	68.25	73.78	74.06	71.63	61.38	47.67	43.6
4 hr	58.22	67.76	74.26	78.33	82.86	69.41	67.16
6 hr	39.78	49.19	57.07	66.56	82.22	80.43	79.95
≥8 hr	33.52	41.95	49.98	56.4	73.29	82.1	85.36

PT6	0 hr	1 hr	2 hr	4 hr
0 hr	79.55	56.01	57.82	48.59
1 hr	69.34	74.87	74.22	63.87
2 hr	58.18	65.66	74.79	74.41
4 hr	15.5	47.93	52.19	57.73
6 hr	-4.98	23.76	20.76	26.1
≥8 hr	-9.16	12.12	11.56	17.85

PT7	0 hr	1 hr	2 hr	4 hr	6 hr	≥8 hr
0 hr	67.49	29.12	15.59	-5.74	2.16	-9.81
1 hr	66.68	49.18	29.11	1.86	14.37	-0.8
2 hr	55.28	53.39	45.66	13.19	28.36	16.77
4 hr	19.5	37.37	39	59.81	63.11	59.26
6 hr	7.12	35.42	38.97	71.2	84.6	81.48
≥8 hr	1.6	21.21	28.42	66.6	84.23	89.07

PT8	0 hr	1 hr	2 hr	4 hr	6 hr
0 hr	65.89	47.81	11.42	-18.98	-27.96
1 hr	53.19	51.6	24.08	-19.77	-14.47
2 hr	40.84	37.77	49.54	9.75	10.54
4 hr	15.51	7.16	52.62	64.83	65.42
6 hr	5.87	3.4	40.03	63.23	88.56
≥8 hr	5.02	-4.33	28.08	62.32	89.87

PT9	0 hr	1 hr	2 hr	4 hr
0 hr	76.88	65.92	53.25	19.39
1 hr	70.16	74.75	73.86	39.33
2 hr	62.05	70.06	75.8	57.65
4 hr	25.27	35.53	46.54	83.88
6 hr	0.11	3.12	16.76	65.33
≥8 hr	-4.53	-0.74	10.07	59.85

PT10	0 hr	1 hr	2 hr	4 hr	6 hr	≥8 hr
0 hr	75.08	58.59	49.37	-6.78	-18.76	-14.05
1 hr	59.09	61.65	62.62	-1.49	-17.26	-13.73
2 hr	41.8	54.18	57.37	27.25	2.33	7.38
4 hr	-14.13	4.1	15.24	49.62	60.31	53.39
6 hr	-25.67	-7.35	-1.34	39.27	69.44	63.88
≥8 hr	-28.43	-16.76	-11.36	32.66	66.6	71.96

PT11	0 hr	1 hr	2 hr	4 hr	6 hr	≥8 hr
0 hr	72.19	57.82	37.53	33.39	-9.68	-15.74
1 hr	68.33	70.88	59.55	52.73	9.39	-4.37
2 hr	52.16	58.77	64.62	58.98	24.01	10.89
4 hr	21.67	30.1	41.92	67.55	63.69	62.58
6 hr	0.53	8.28	21.73	56.83	79.14	77.65
≥8 hr	-2.45	0.99	12.82	46.93	69.75	83.58

PT12	0 hr	1 hr	2 hr	4 hr	6 hr
0 hr	77.33	61.35	25.31	6.46	-0.25
1 hr	66.83	75.48	41.15	22.66	15.84
2 hr	64.51	74.94	64.68	51.41	38.81
4 hr	22.42	54.01	62.68	75.14	71.21
6 hr	-5.55	23.63	47.51	59.27	85.65
≥8 hr	-8.25	15.02	38.83	54.14	81.81

Supplementary Fig. 6. Correlation between each time point within each study subject vs. the grand average for time points. Note that subjects generally correlate with the correct time points, indicating that time after surgery was by far the largest driver of variance, and that no samples were statistical outliers in the analysis. Notes that patient 5 had an additional sample (surgical discard) collected at 11 hours that is included in this analysis and correlates with 6 hrs and 13.5 hours for that patient (closure).

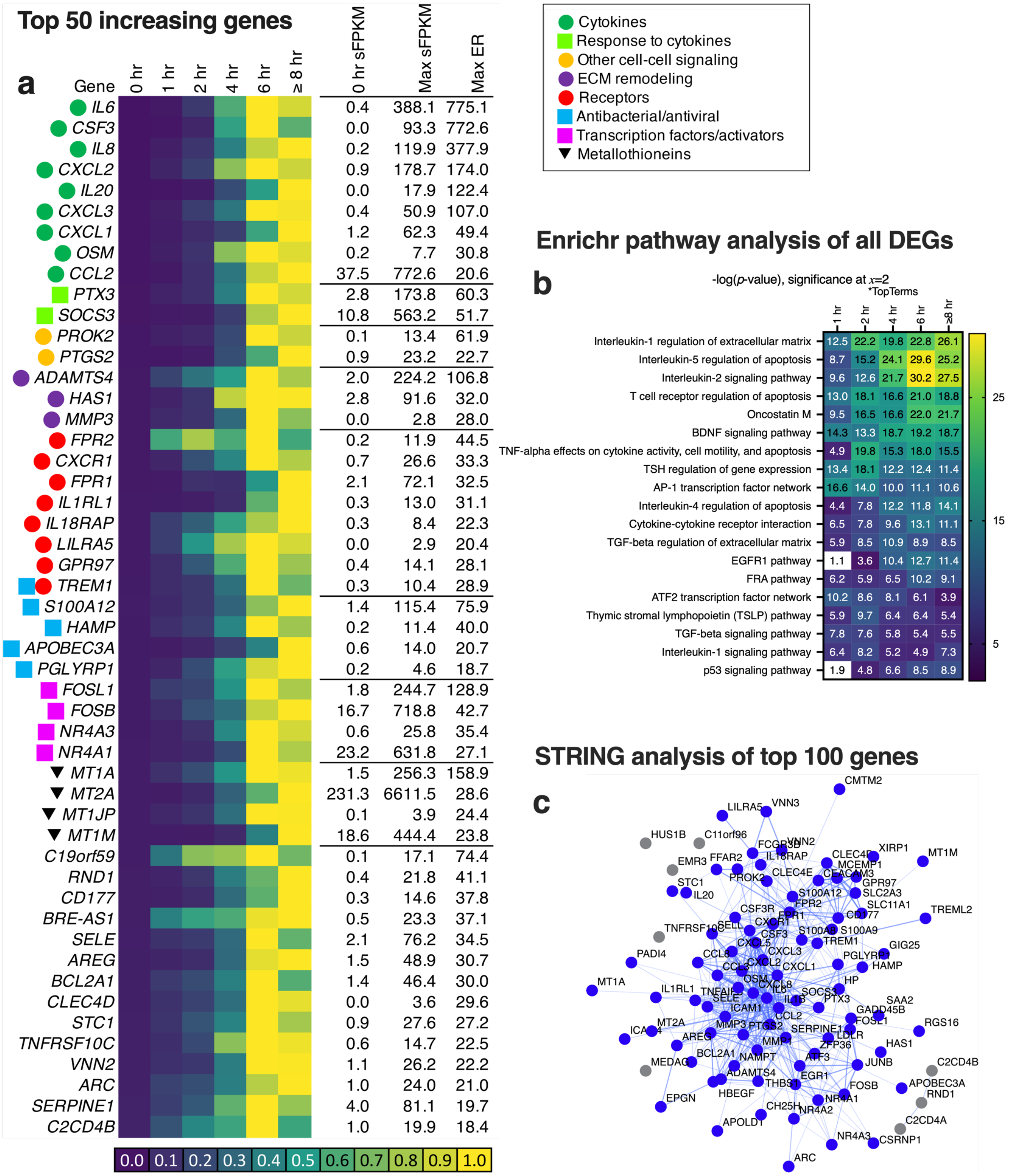

Supplementary Fig. 7. Top significant genes and pathways from transcriptomic analyses of longitudinal incision study. The significantly increasing genes were ranked by fold induction and ordered into the top 50 significantly changing genes induced by incision by considering the maximum expression ratio (usually control vs. 6 or 8 hours). **a** These top 50 genes were organized into functional categories, with the largest group comprising cytokines (9 genes, green circles), and receptors (8 genes, red

squares). Note that many genes in the cytokine group start at very low expression levels (< 1 sFPKM) and increase >100-fold. Three of these genes are interleukins (interleukins 6, 8 and 20). Additionally, chemokine/interleukin-like receptors and accessory proteins are among the most induced receptor genes (*IL1RL1* and *IL18RAP*). **b** In a STRING analysis of the top 100 gene, we observed that the majority of induced genes after incision have been described as interacting, suggesting that in some senses the induction pattern is an integrated set of pathways that interacts with itself. **c** Enrichr was used to identify broadly significant pathways identified among all induced genes. Consistent with some of the genes in the top 50 significant genes, top categories included several interleukin signaling pathways as well as the oncostatin M pathway.

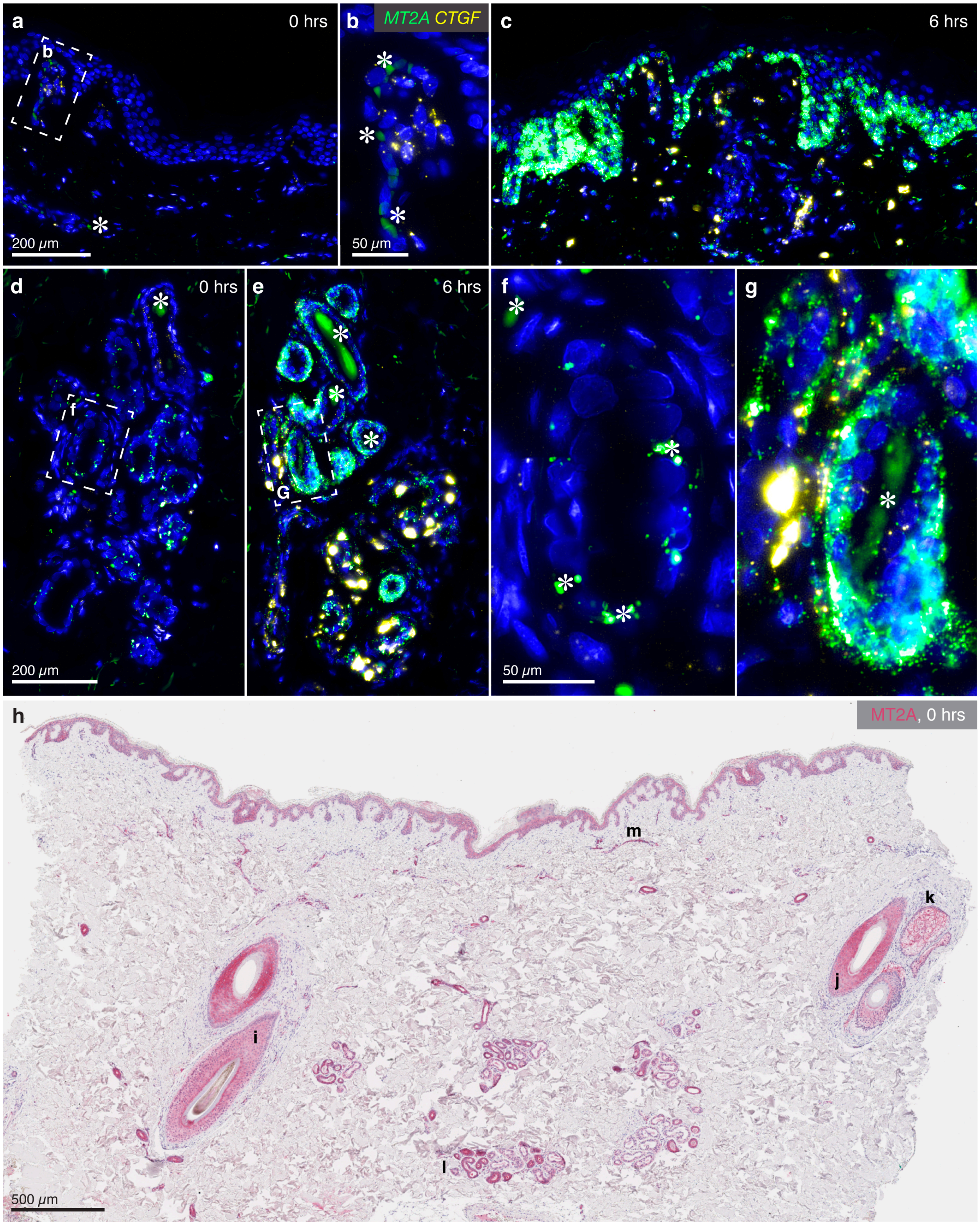

a
0 hrs
b
200 μm
b
MT2A CTGF
50 μm
c
6 hrs
d
0 hrs
f
200 μm
e
6 hrs
G
f
50 μm
g
h
MT2A, 0 hrs
m
k
j
i
l
500 μm

Supplementary Fig. 8. Anatomical localization of transcripts encoding the metal-binding protein Metallothionein 2A (MT2A) after surgical incision.

Anatomical localization of Metallothionein 2A transcript (*MT2A*) was assessed through in situ hybridization and immunostaining. **a** A cross section of a region of the epidermis (enlargement shown in **b**) and dermal tissue is shown for the 0 hr control, where little staining for the mRNA was observed. **b** At baseline, *MT2A* is not detected, but connective tissue growth factor (*CTGF*) is present at a low amount. Asterisks denote autofluorescent signal from blood and from gland structures when sweat glands are present. Note that by RNA-Seq, relatively high levels of MT2A were detectable in baseline conditions, indicating that the apparent negative result at 0 hrs could be due to assay sensitivity. **c** A cross section of epidermal and superficial dermal tissue from the same patient at the 6 hr timepoint shows marked *MT2A* and *CTGF* induction. We note that *MT2A* was strongly induced in various discrete regions of the epidermis, such as the representative field shown, but was not uniformly induced across the entire epidermal layer. **d** A cross section of a sweat gland is shown at 0 hrs (enlargement in **f**). Similar to our findings in the dermal tissues, expression of *MT2A* and *CTGF* is not observed at this time point. **e** In a cross section of a sweat gland from the same patient at 6 hrs (enlargement in **g**), *MT2A* is highly induced in the circular, secretory coils of the gland while *CTGF* is induced in both secretory coils and sweat ducts. **h** A representative field of a whole skin tissue sample showing antibody staining of MT2A protein. While the overall staining pattern was highly similar in that gland tissue and keratinocyte layers were densely labeled, there was essentially no difference between the control and subsequent time points, and the staining pattern in general was much stronger and more widespread. We cannot rule out off target labeling of other MT proteins (which are also highly expressed in skin), although it is notable that the protein labeling also comes from a different pool because protein turnover of highly expressed structural proteins can vary substantively from the mRNAs encoding them. Thus, it is plausible that the mRNA result is representative of the induced pool, whereas the broader less specific staining for the protein represents a static pool. However, this was not possible to resolve in the present study. MT2A was also labeled by this antibody in other various structures of the skin such as hair follicles (**i** and **j**), glandular structures including sebaceous glands (**k**) and sweat glands (**l**), and blood vessel vasculature (**m**).

a **G-protein coupled receptors**

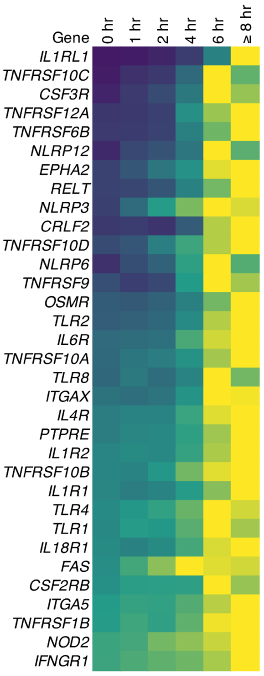

Gene	0 hrs sFPKM	Max sFPKM	Max ER
IL1RL1	0.3	31.1	31.1
TNFRSF10C	0.6	14.7	22.5
CSF3R	2.9	46.8	15.8
TNFRSF12A	20.2	148.5	7.3
TNFRSF6B	2.2	15.1	6.7
NLRP12	0.1	4.0	5.9
EPHA2	22.7	130.9	5.7
RELT	3.4	19.7	5.7
NLRP3	0.8	5.1	5.5
CRLF2	0.2	5.1	5.1
TNFRSF10D	3.0	14.5	4.7
NLRP6	0.1	3.0	4.5
TNFRSF9	0.4	3.0	3.8
OSMR	15.3	57.2	3.7
TLR2	3.3	12.0	3.6
IL6R	12.4	38.4	3.1
TNFRSF10A	1.9	5.9	3.0
TLR8	0.8	2.7	3.0
ITGAX	5.3	15.0	2.8
IL4R	25.1	63.9	2.5
PTPRE	10.4	26.7	2.5
IL1R2	25.0	59.5	2.4
TNFRSF10B	11.6	27.6	2.4
IL1R1	40.5	95.3	2.3
TLR4	6.8	15.4	2.3
TLR1	1.8	4.1	2.2
IL18R1	3.1	7.0	2.2
FAS	10.2	22.6	2.2
CSF2RB	12.5	27.3	2.2
ITGA5	30.0	59.5	2.0
TNFRSF1B	21.3	42.2	2.0
NOD2	6.9	12.5	1.8
IFNGR1	63.2	113.0	1.8

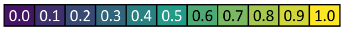

b **Catalytic receptors**

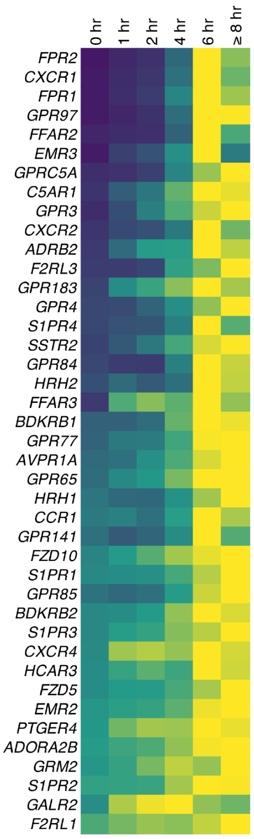

Gene	0 hrs sFPKM	Max sFPKM	Max ER
FPR2	0.2	11.9	44.5
CXCR1	0.7	26.6	33.3
FPR1	2.1	72.1	32.5
GPR97	0.4	14.1	28.1
FFAR2	1.2	16.3	13.0
EMR3	0.0	1.5	11.0
GPRC5A	2.0	20.4	9.8
C5AR1	4.0	33.1	8.1
GPR3	0.3	2.8	8.0
CXCR2	3.8	30.7	7.9
ADRB2	7.9	55.7	7.0
F2RL3	0.7	4.9	6.0
GPR183	4.9	27.2	5.5
GPR4	1.7	9.2	5.0
S1PR4	1.9	9.6	4.9
SSTR2	0.4	2.1	4.8
GPR84	0.5	2.7	4.5
HRH2	0.3	1.3	3.6
FFAR3	0.1	0.5	3.6
BDKRB1	2.3	8.1	3.4
GPR77	0.9	3.2	3.2
AVPR1A	4.4	13.6	3.1
GPR65	0.9	2.8	2.8
HRH1	5.4	14.7	2.7
CCR1	2.8	7.4	2.6
GPR141	0.4	1.1	2.5
FZD10	8.0	19.1	2.4
S1PR1	17.1	39.5	2.3
GPR85	0.2	0.6	2.3
BDKRB2	6.1	14.1	2.3
S1PR3	3.7	8.6	2.3
CXCR4	9.3	21.0	2.2
HCAR3	6.5	14.5	2.2
FZD5	2.0	4.6	2.2
EMR2	2.9	5.9	2.0
PTGER4	7.6	15.5	2.0
ADORA2B	7.5	14.8	2.0
GRM2	0.6	1.3	1.9
S1PR2	6.9	12.9	1.9
GALR2	0.2	0.3	1.7
F2RL1	20.7	34.8	1.7

Supplementary Fig. 9. Significantly upregulated/induced receptor genes. Genes significantly increasing at any time point (1163 genes total) were segregated into functional categories using database searching, as described in the methods for Fig. 3. A database of functional categories useful for pharmacology (IUPHAR database) was used to categorize genes into **a** G-protein coupled receptor and **b** catalytic receptor categories. The significant genes in each category are plotted.

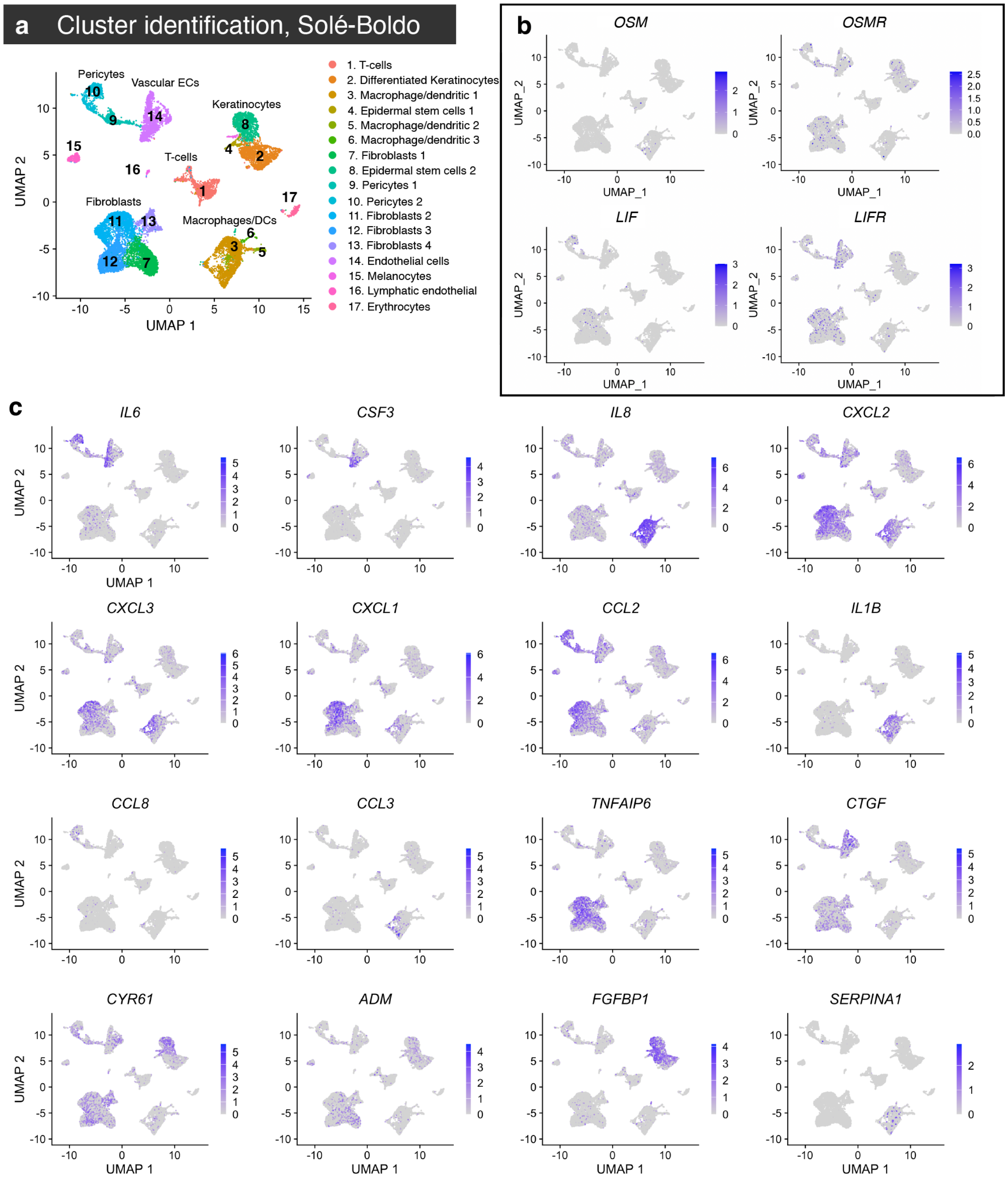

Supplementary Fig. 10. Utilization of previously published single-cell sequencing data to characterize gene expression profiles of secreted factors in incised skin. Data from a previously published manuscript examining single-cell sequencing from human skin samples was mined to interrogate genes of interest in the present study.[1] **a** Solé-Boldo, et al. identified 17 clusters of cells. **b** Two of the major signaling/receptor pairs in the dataset OSM/OSMR and LIF/LIFR were tested in this dataset, and showed marginal data, emphasizing the need for anatomical confirmation. *OSMR* appeared to be expressed in most skin tissues, and *LIFR* was most prominent in endothelial cells and fibroblasts. **c** A selection of genes from the "secretome" analysis analyzed for enrichment in specific skin structures. Note that skin samples in Solé-Boldo were taken

intraoperatively after incision start and appear to show induction of many incision-specific genes. Presumably, there is a variable degree of incision-induced gene signatures in the dataset, which make it useful for identifying cellular origin of genes which are not expressed at baseline such as *IL6*.

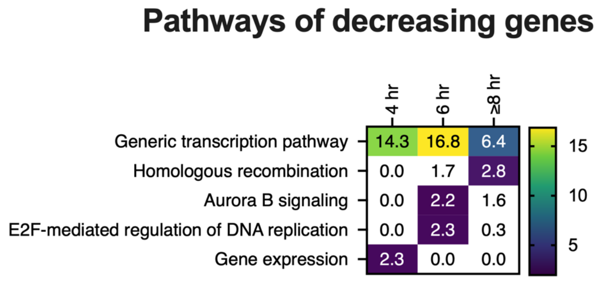

Supplementary Fig. 11. Significant pathways among decreasing genes using Enrichr. Analogous to the analysis performed for increasing genes, the significant decreasing genes were probed for biological functions using Enrichr (see Supplementary Fig. 7b). Generic transcription was the most highly significant regulated pathway. We were not able to draw strong conclusions from the decreasing genes, and as they have little consistent pattern, we assume that this is a non-specific trauma related decrease in many transcripts at once. This phenomenon may be interesting to investigate in future studies.

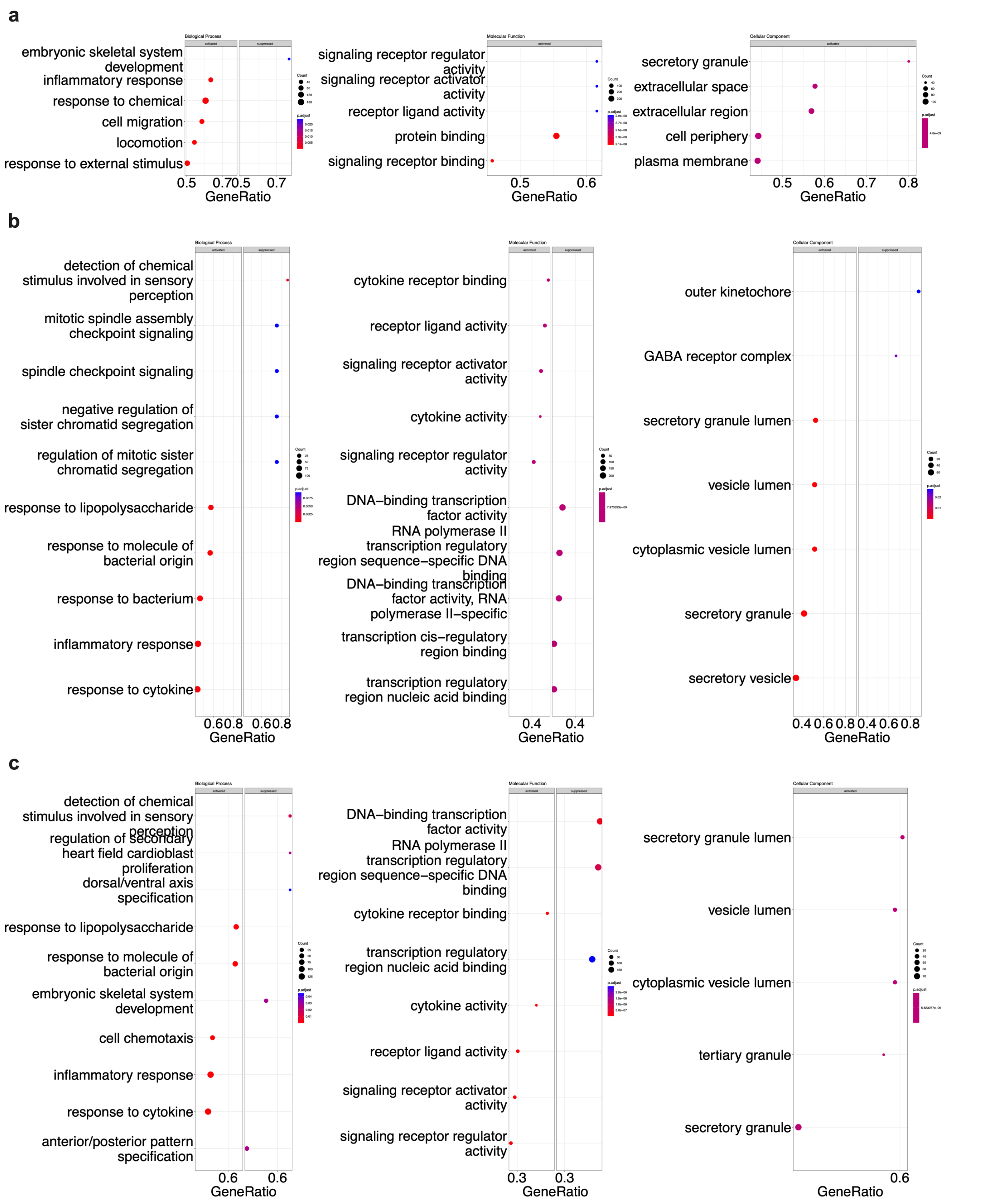

Supplementary Fig. 12. Gene Set Enrichment Analysis for each timepoint.
a 4 hours, **b** 6 hours, and **c** Closure. The top 5 gene sets are shown. The total number of genes from GSEA analysis are summarized in Supplementary Table 8.

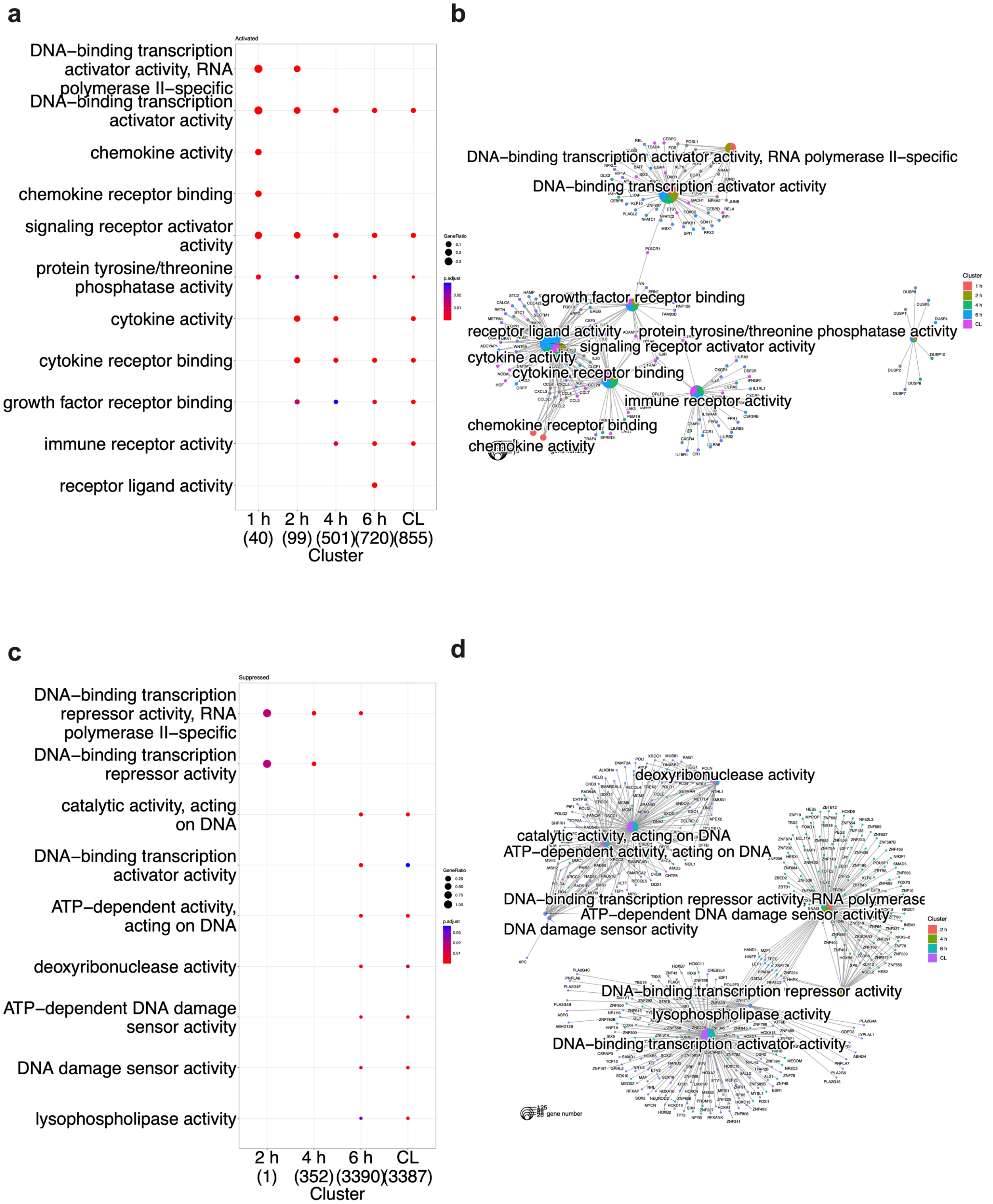

Supplementary Fig. 13. Comparing DEG clusters of each timepoints.
a A dot plot and **b** a plot of semantic similarity for activated gene sets. **c** A dot plot and **d** a plot of semantic similarity for suppressed gene sets. The top 5 gene sets are shown.

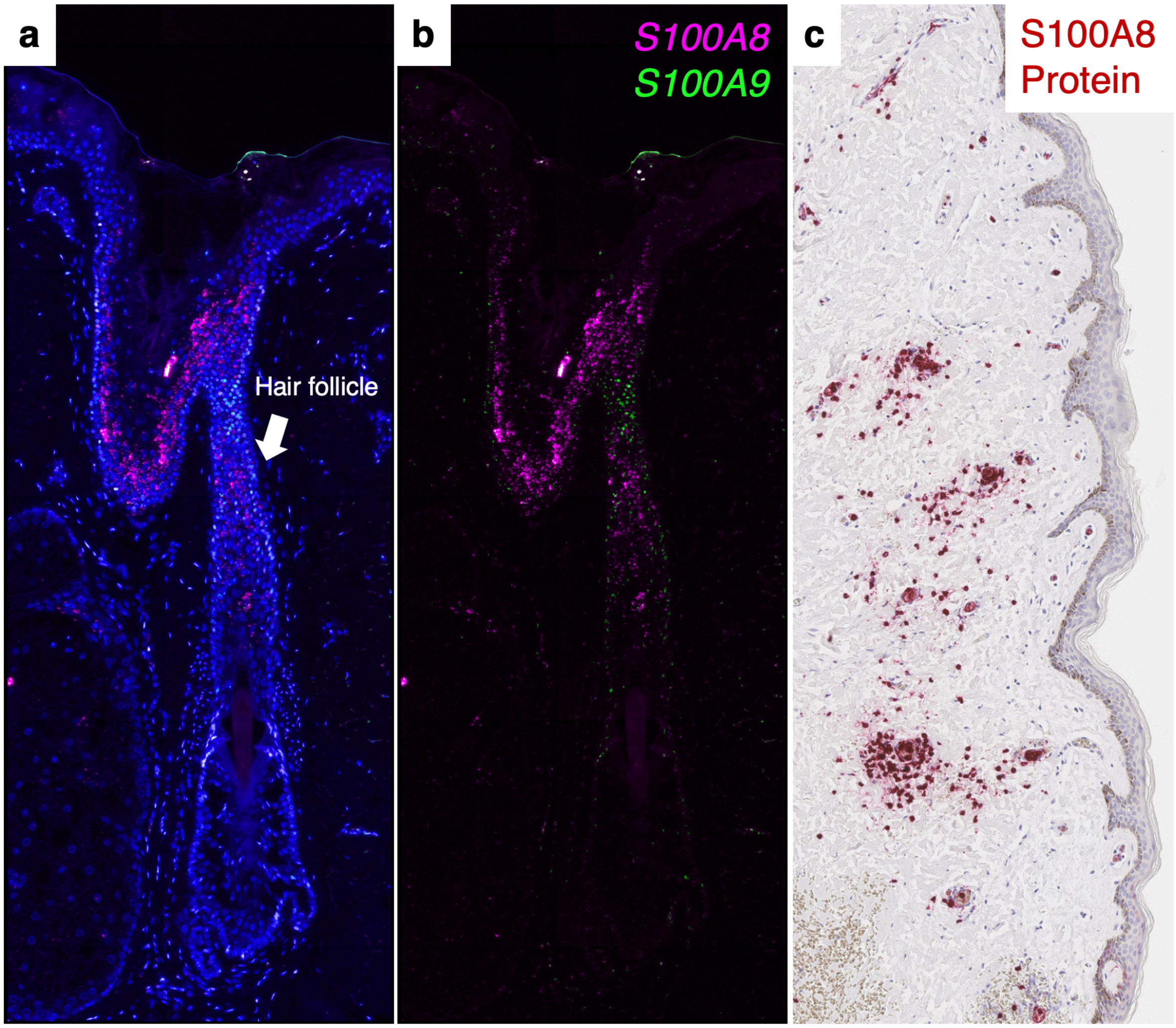

Supplementary Fig. 14. Anatomical localization of transcripts encoding S100A8 and S100A9 after surgical incision. Anatomical localization of highly induced genes *S100A8* and *S100A9* was assessed through in situ hybridization and immunostaining. **a** A cross section of a hair follicle opening up to the epidermis in a human skin tissue sample. *S100A8* and *S100A9* are induced in hair follicles following tissue injury. A 2-channel image without DAPI is shown to better visualize expression of S100 markers in **b**. **c** Immunostaining shows that S100A8 protein is also widely expressed in damaged skin tissue.

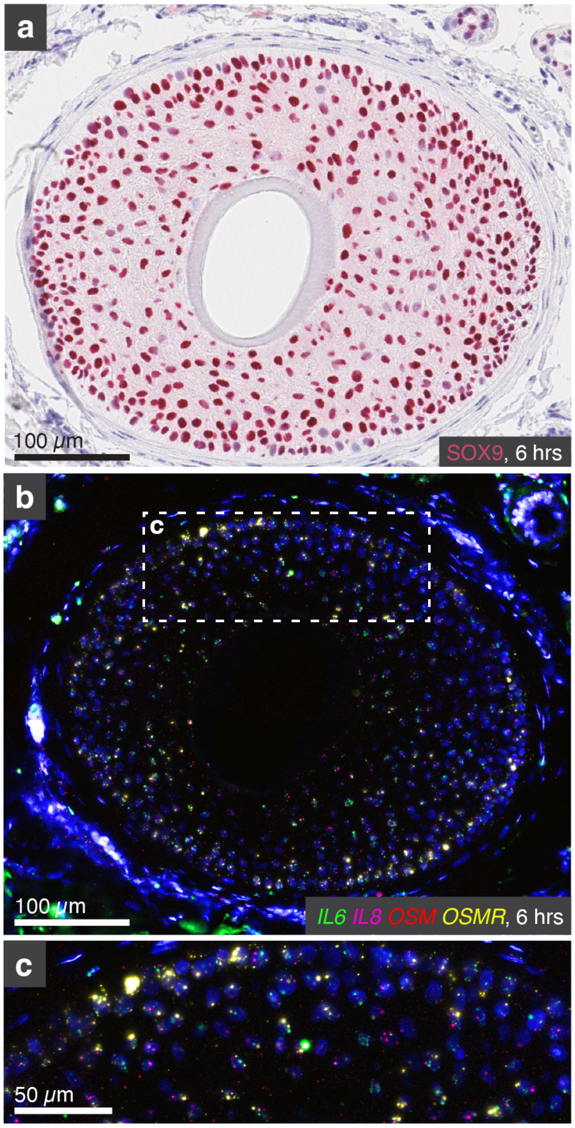

Supplementary Fig. 15. Genes induced by surgical incision are coexpressed with stem cell marker SRY-Box Transcription Factor 9 (SOX9)*.* Stem cells of the skin have been thought of as active players in pain signaling and wound healing pathways.[2] Antibody staining for SOX9, which marks stem cells in the bulge of the hair follicle,[3] was performed to determine if highly induced genes were expressed in hair follicle stem cells. **a** Antibody staining of SOX9 marks cells throughout the hair follicle. **b** A cross section of hair follicle in human skin tissue with 4-plex staining for *IL6*, *IL8*, *OSM*, and *OSMR*. (enlargement in **c**). Induced genes are coexpressed with SOX9, suggesting a functional role for stem cells in the nexus of early nociceptive signaling.

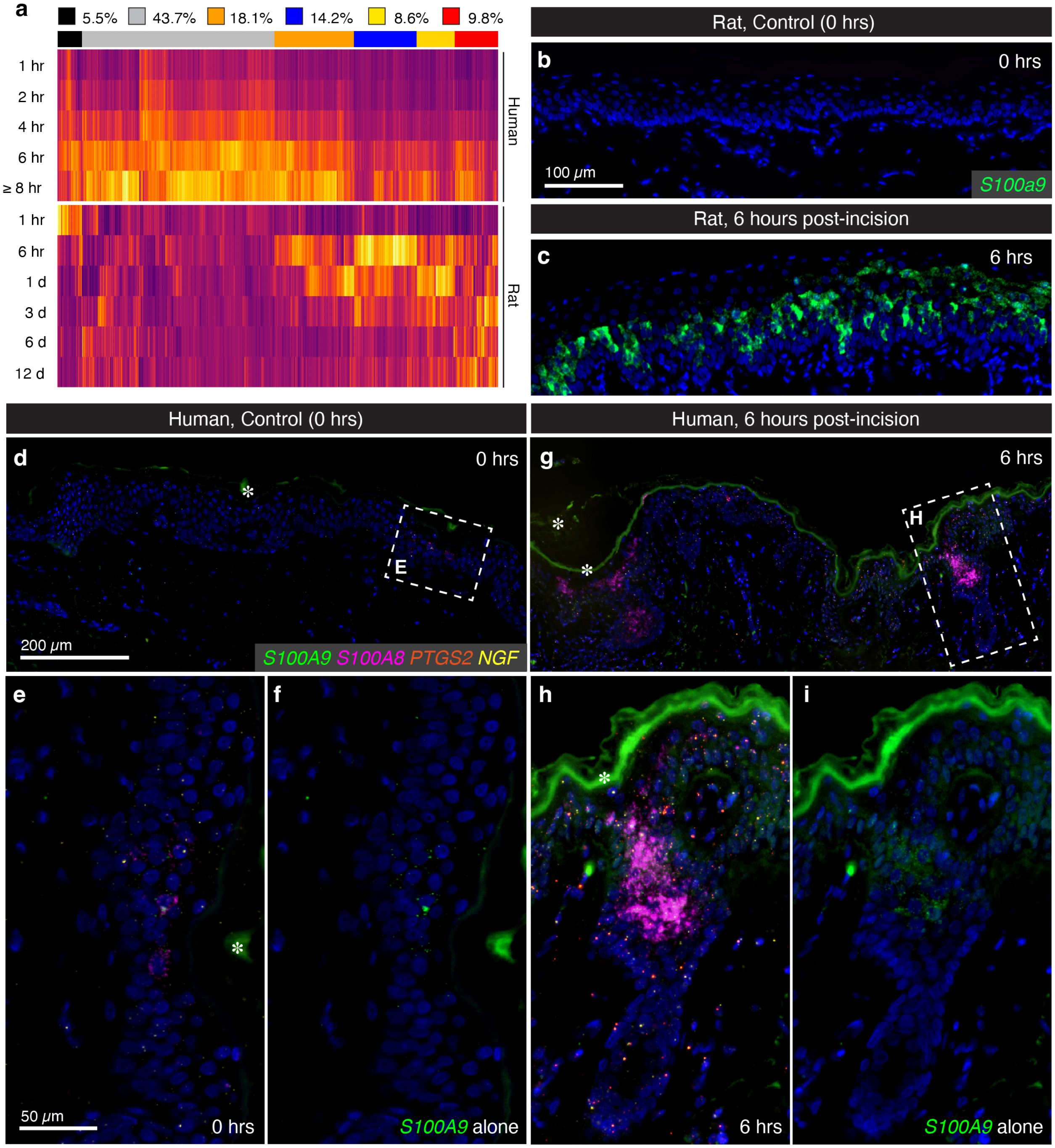

Supplementary Fig. 16. Gene transcriptional changes following tissue injury vary in the human and the rat. We investigated species differences in gene transduction events following incision using a bioinformatic clustering analysis and in situ hybridization staining. **a** A heatmap showing differences in transcriptome induction following incision in the human and rat. Upregulation of genes is represented with increasing bright intensity (yellow) tiles and downregulation of genes is represented with darker intensity (purple) tiles. 6 major patterns were identified (colors), with the greatest similarity between rat and human being between genes induced in human at 6 hours and the 6 hour time point in the rat model (blue and yellow). Approximately 43.7% of the

human genes (gray) were not obviously induced in rat. A small number of genes (black) were induced in human but associated with the 1 hour time point in the rat model. In **b-c.** *S100a9* expression is examined in the rat pre- and post-incision. **b** A cross section of epidermis of rat plantar paw tissue at baseline. **c** Upon tissue damage, *S100a9* is strongly induced throughout the entirety of the epidermal layer. **d-i** In human skin tissue, we observed expression of *S100A9*, *S100A8*, *PTGS2*, and *NGF*. **d** At time zero, *S100A8*, *S100A9*, and *NGF* are expressed at low levels in the epidermis (**e.** enlarged view. **f** A single-channel image of *S100A9* alone. Asterisks denote the high autofluorescence from human skin. **g** *S100A8*, *S100A9*, and *PTGS2* are notably induced in the epidermis after surgical incision (**h.** enlarged view). **i** *S100A9* is induced in localized regions of the epidermis, in contrast to the rat which shows induction in a large area around the incision.

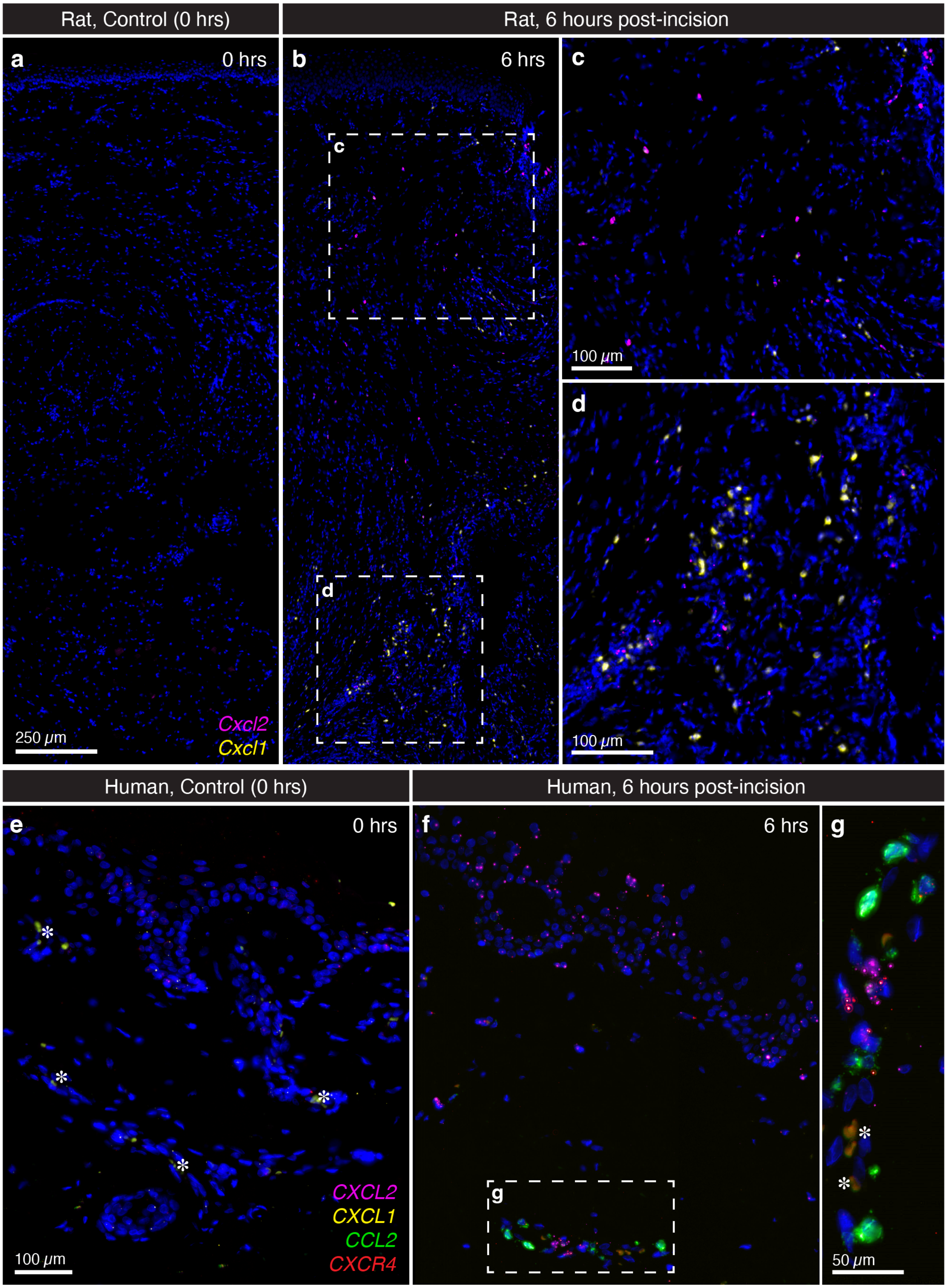
Rat, Control (0 hrs)
Rat, 6 hours post-incision
a
0 hrs
b
6 hrs
c
d
250 μm
Cxcl2
Cxcl1
100 μm
100 μm
Human, Control (0 hrs)
Human, 6 hours post-incision
e
0 hrs
f
6 hrs
g
*
CXCL2
CXCL1
CCL2
CXCR4
100 μm
50 μm

Supplementary Fig. 17. Species differences in induction patterns of Chemokine ligands 1 and 2 (*CXCL1*, *CXCL2*) in human and rat. Comparison of gene transcriptional changes between human and rat incision surgical cases was assessed through in situ hybridization of strongly induced genes encoding for chemokines. In **a-d.** we examined localization of *Cxc1* and *Cxcl2* markers in rat. **a** A cross section of rat plantar paw tissue at time zero. At the control timepoint, *Cxcl1* and *Cxcl2* are not detected. **b** At 6 hours post-incision, *Cxcl1* and *Cxcl2* are induced. **c** *Cxcl2* is widely expressed in the tissue both superficially in distinct parts of the epidermis and deeper in presumptive fibroblasts while **d** *Cxcl1* induction is concentrated to deeper parts of the tissue. In **e-g.** we investigate a 4-plex for *CXCL2*, *CXCL1*, *CCL2* (C-C Motif Chemokine Ligand 2), and *CXCR4* (C-X-C Motif Chemokine Receptor 4) transcripts in human. **e** A cross section of human skin tissue at baseline. *CXCL1* and CXCR4 are lowly detected in the epidermal layer. Asterisks mark autofluorescence of red blood cells. **f** *CXCL2*, *CXCL1*, *CCL2*, and *CXCR4* are induced by injury to tissue. Unlike chemokine induction in the rat, induction patterns of *CXCL1* and *CXCL2* in the human are more superficial. *CXCL2* is induced throughout the epidermis and in blood vessels (enlargement in **g**), which *CCL2* and *CXCR4* are also localized to.

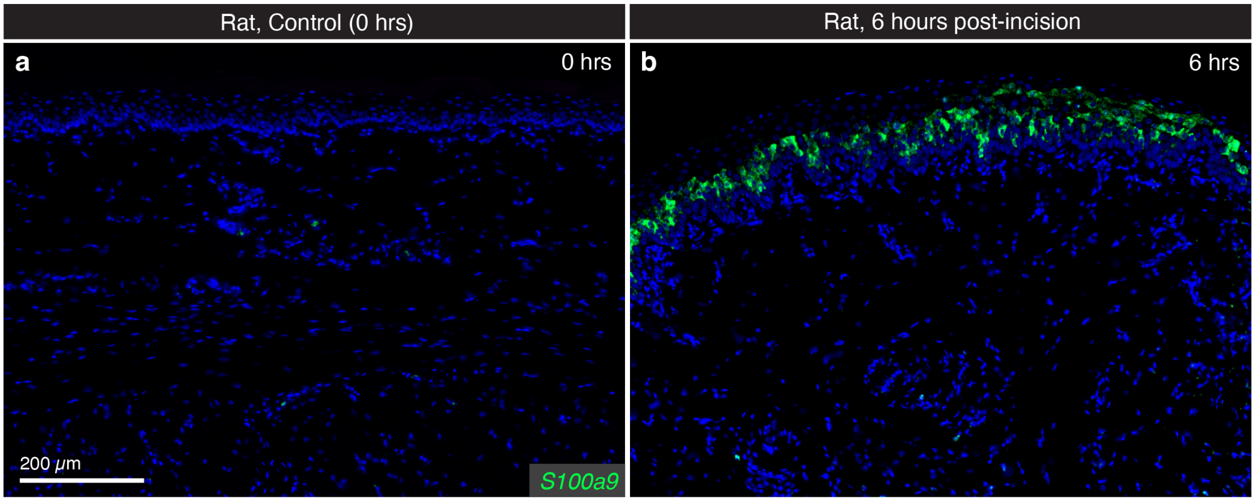

Supplementary Fig. 18. Keratinocyte *S100a9* induction in rat is localized to the epidermis. As one of the genes that are strongly induced following tissue injury in the Brennan rat model of surgical incision[5], anatomical distribution of *S100a9* was investigated through in situ hybridization. In **a-b**, we show wide view images of *S100a9* induction in rat plantar paw tissue. **a** A cross section of rat plantar paw tissue at time zero. **b** Following surgical incision, *S100a9* induction is observed across the entire epidermal region.

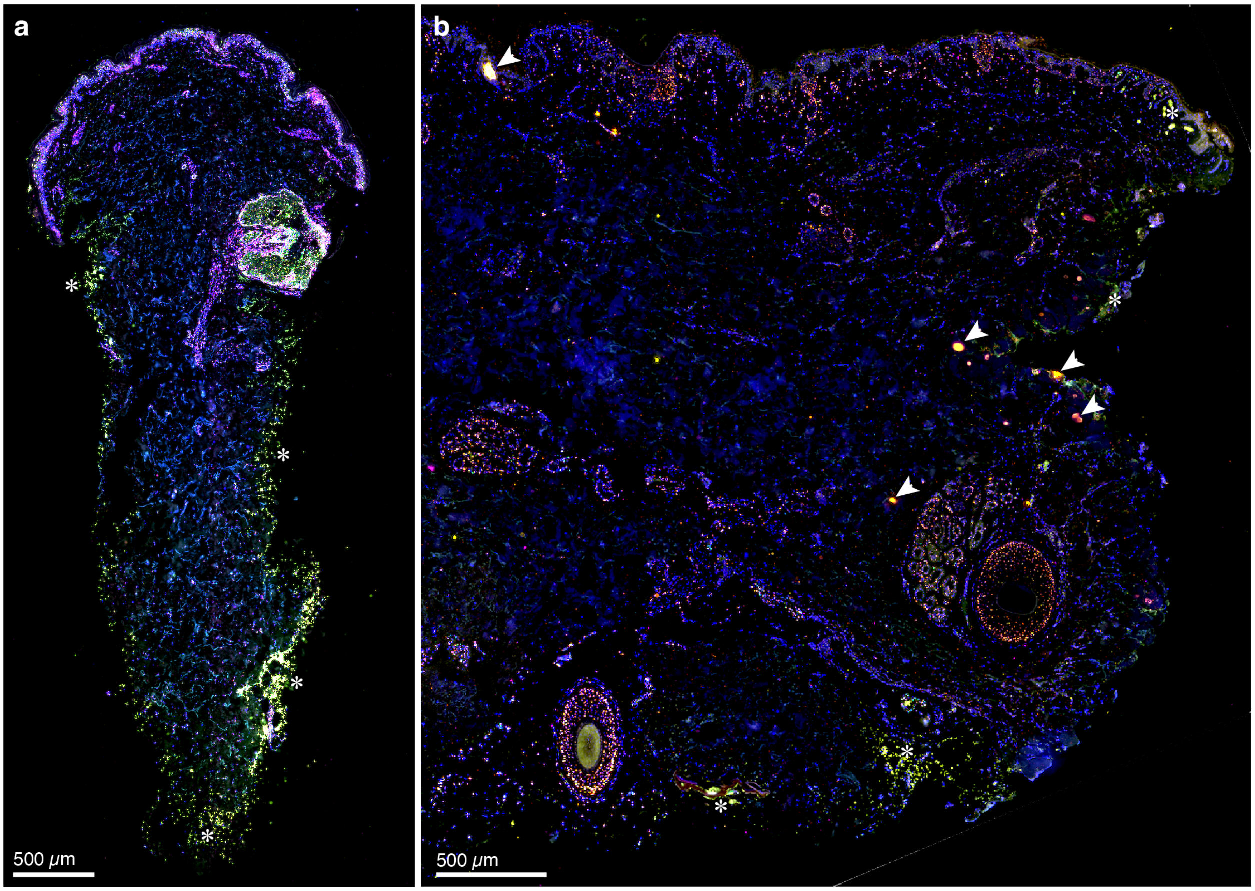

Supplementary Fig. 19. Diagrammatic image of fluorescently labeled standard skin punch biopsy relative to samples collected in the present study. Note that the enhanced size allows for visualization of full cross-sectional views of hairs in epidermal layers that would be disrupted by circular punches. Also note the profound shrinkage that occurs in circular skin punch, which is more pronounced distal to the epidermal layer. Arrowheads and asterisks mark autofluorescence of skin tissue and red blood cells, respectively. The final merged composite was enhanced in Preview (Version 11.0, 1056.5.1, Apple, Cupertino, CA) to achieve visibility for the fluorescence image for widefield view.

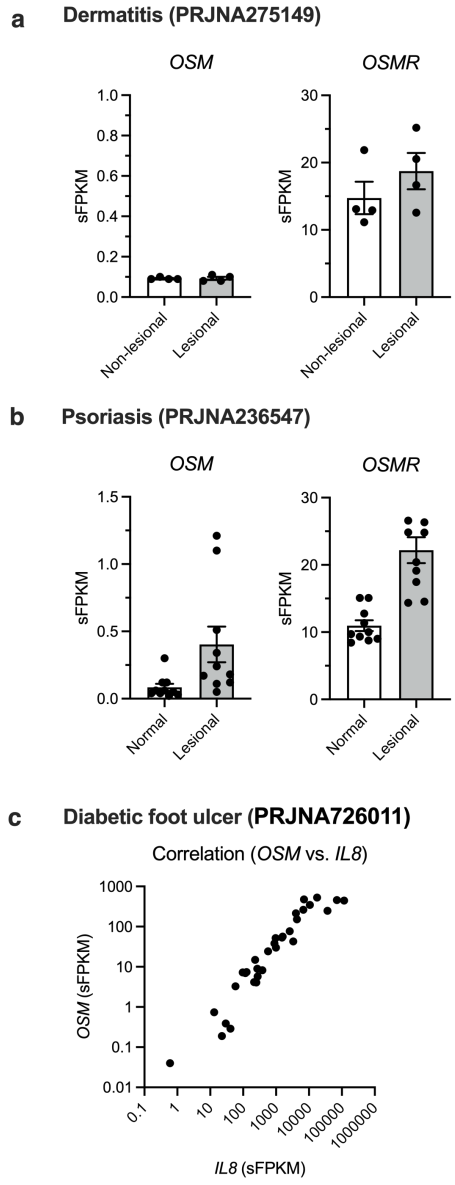

Supplementary Fig. 20. Analysis of Oncostatin M and its receptor expression in skin conditions associated with itch and wounding in previously published dataset. Previous reports have claimed that Oncostatin M is strongly induced in itchy human skin conditions such as dermatitis and psoriasis. In the present analysis, we reanalyzed both the ligand and the receptor in these datasets and found that oncostatin M was **a** not induced in dermatitis in a previously published dataset, and **b** was only marginally induced in psoriasis, contrasting with **c** the profound induction that occurs in diabetic foot ulcer, as indicated by the correlation with *IL8* in heterogeneous samples from diabetic foot ulcers of different types and/or severities. This evidence is consistent with the observations from the present study showing *OSM* induction is highly significant in incised skin, suggesting it may be more relevant to wounding and/or pain than itch.

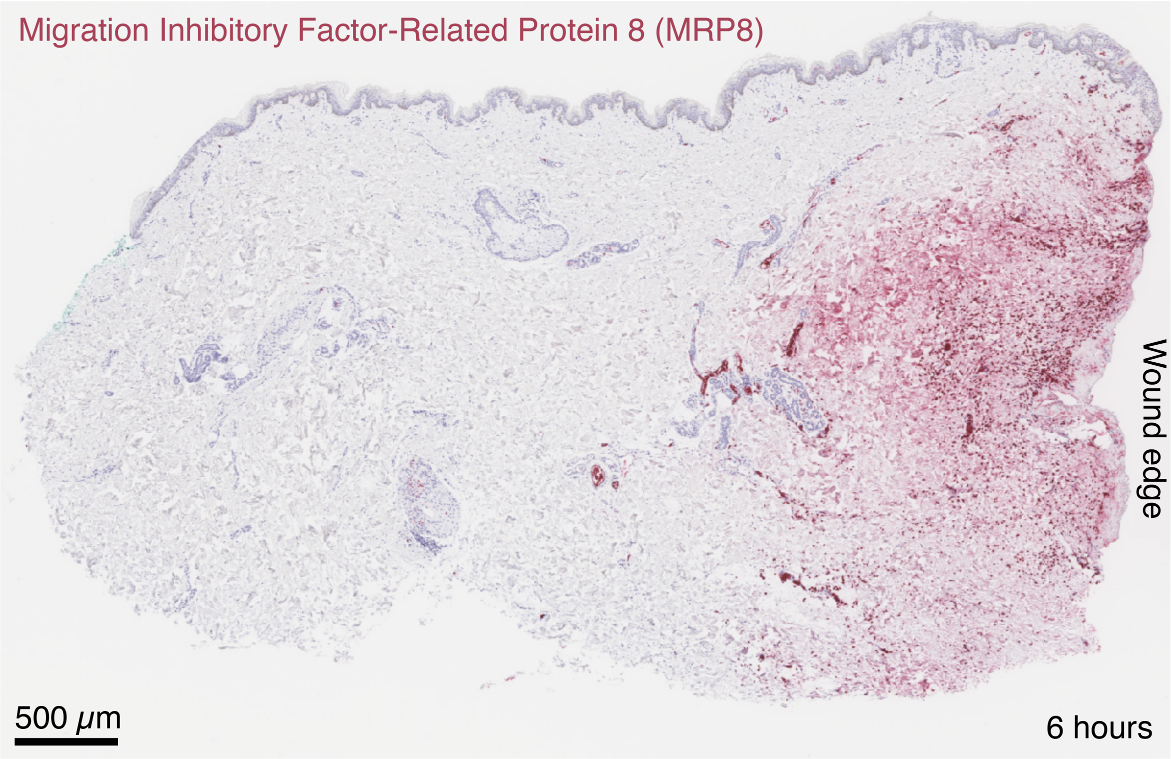

Supplementary Fig. 21. Replication of results of Calprotectin antibody using an antibody against MRP8. Calprotectin stains for the dimer of the proteins encoded by *S100A8* and *S100A9*. In the course of characterizing the staining pattern of Calprotectin in human skin after surgery, we also evaluated the protein encoded by *S100A8*, Migration inhibitory factor-related protein 8 (MRP8). The staining pattern was highly similar between these two approaches. A representative image of staining at 6 hours after incision (highly induced towards the wound edge) is shown. Note that a neighboring section was edited out of the final image for presentation (right side).

Supplementary Tables 1-5 are large formatted tables containing raw data points and are uploaded alongside this manuscript 10.6084/m9.figshare.27384174.

Supplementary Table 6. Demographics information for DRG donors.

	**DRG (Autopsy)**
Median age (range) – yr	34 (21-53)
Gender – no. (%)	
Female sex	3 (75.0)
Male sex	1 (25.0)
Race or ethnic group – no. (%)	
White	3 (75.0)
African American	1 (25.0)
Cause of death – no. (%)	
Anoxia/Cardiovascular	1 (25.0)
Anoxia/Drug intoxication	1 (25.0)
CVA/ICH/Stroke	2 (50.0)
Lumbar Level - no. (%)	
L3	3 (75.0)
L4	1 (25.0)

Supplementary Table 7. Catalogue numbers for Advanced Cell Diagnostic in situ hybridization probes.

Figure	Gene name	Symbol	Reference no.	Lot no.
4, 5	oncostatin M	*OSM*	456381 – C1	21299B
	oncostatin M receptor	*OSMR*	537121 – C2	21299E
	interleukin 8	*IL8*	310381 – C3	22074C
	interleukin 6	*IL6*	310371 – C4	22074C
5	tachykinin 1	*TAC1*	310711- C1	22194A
	oncostatin M receptor	*OSMR*	537121 - C2	21299E
	transient receptor potential vanilloid 1	*TRPV1*	415381 - C3	23103C
	opioid receptor mu 1	*OPRM1*	410681 - C4	23180F
6, S17	C-X-C motif chemokine ligand 2	*CXCL2*	425251 - C1	N/A
	C-X-C motif chemokine ligand 1	*CXCL1*	427151 - C2	N/A
	C-X-C motif chemokine receptor 4	*CXCR4*	310511 - C3	N/A
	C-C motif chemokine ligand 2	*CCL2*	423811 - C4	N/A
7	interleukin 6 receptor	*IL6R*	557201 - C1	24158C
	colony stimulating factor 3 receptor	*CSF3R*	468721 - C2	24158F
	transmembrane protein 119	*TMEM119*	478911 - C3	23180E
	transient receptor potential vanilloid 1	*TRPV1*	415381- C4	24051B
	prostaglandin E receptor 2	*PTGER2*	406791 - C1	22363A
	prostaglandin E receptor 4	*PTGER4*	406771 - C2	24149B
	prostaglandin E receptor 3	*PTGER3*	488431 - C3	24149B
	transient receptor potential vanilloid 1	*TRPV1*	415381 - C4	24051B
	solute carrier family 40 member 1	*SLC40A1*	1185601 - C1	24156B
	oncostatin M receptor	*OSMR*	537121 - C2	24108A
	LIF receptor subunit alpha	*LIFR*	441021 - C3	24158F
	transient receptor potential vanilloid 1	*TRPV1*	415381 - C4	24051B
S8	metallothionein 2A	*MT2A*	525418 - C3	N/A
S17	C-X-C motif chemokine ligand 1	*Cxcl1*	407721- C1	N/A
	C-X-C motif chemokine ligand 2	*Cxcl2*	437581 - C2	N/A
S16, S18	prostaglandin-endoperoxide synthase 2	*PTGS2*	406801 - C1	23228B
	S100 calcium binding protein A9	*S100A9*	417581 - C2	24156A
	nerve growth factor	*NGF*	549451 - C3	24156A
	S100 calcium binding protein A8	*S100A8*	425271 - C4	24156B
	S100 calcium binding protein A9	*S100a9*	527711 – C3	N/A

Supplementary Table 8. Number of gene sets enriched by DEGs

	Number of DEGs	Biological Process		Molecular Function		Cellular Component	
		Activated	Suppressed	Activated	Suppressed	Activated	Suppressed
1 hour	47	0	0	0	0	0	0
2 hours	122	1	0	0	0	0	0
4 hours	1189	199	1	23	0	19	0
6 hours	5643	426	25	50	7	42	2
Closure	5802	406	5	48	3	41	0

C. Supplementary Notes

C1. Lack of interindividual differences among study participants.

This study focuses mainly on group differences pooled across the 12 participants. This analysis was specified in the original clinical protocol and was the major planned analysis. However, exploratory analyses were also investigated to delineate potential differences based on disease status, treatment, or sex of participant. However, the major source of variance among the samples was the time point. This means that for most of the 0 hr time points, the closest samples by correlation analysis were other 0 hr time points, and the furthest were ≥8 hr time points, regardless of participant (Supplementary Fig. 5). Additionally, there was no significant difference (sex*incision) despite several baseline sex differences stemming from genes such as X-chromosome genes which vary across almost all tissues in male vs. female tissue samples regardless of tissue type or condition (Supplementary Fig. 6). One of the minor sources of variation was that some participants correlated slightly better or worse with adjacent time points, although not frequently. Patient 8's 2 hr time point actually correlates better with the grand average 4 hr time point than with other 2 hr time points. This was also evident in some of the sex difference genes, with some of the nominally significant sex different genes showing a difference largely in time of induction rather than amount. However, given that the patients who showed mild differences in induction rate were not uniformly different by sex, we opted for the more conservative route not to emphasize these differences, as they may not be reproducible in a larger cohort, and are unlikely to be due to a single variable (i.e. biological sex alone.)

C2. Top 50 significantly increased genes following surgical incision

Of the 1163 significantly increasing genes from our study, the top 50 were extracted and sorted by induction fold between baseline and maximum expression ratio (Supplementary Fig. 7a). The top three genes by expression ratio (ER ≥ 300) are distinct from most of the other genes, each going from a baseline expression value of close to zero to a robust expression level at the point of maximum induction. These three genes are interleukin 6 (*IL6*) with and expression ratio of 775.1, Colony stimulating factor 3 (*CSF3*) with an expression ratio of 772.6, and interleukin 8 (*IL8*), with an expression ratio of 377.9 (Supplementary Fig. 7a). These top 50 genes were further organized into functional categories, with the largest group comprising cytokines (9 genes, green circles), and receptors (8 genes, red squares). Of the top 3 genes, all of them are in the secreted cytokine family. Of the genes with an expression ratio of ≥ 100, chemokines such as Chemokine (C-X-C motif) ligand 2 (*CXCL2*), and *CXCL3* are represented. One additional interleukin, interleukin 20 (*IL20*) is in this category as well. This indicates the overrepresentation of interleukin signaling pathway genes in the most highly differential genes in the dataset. Other genes with ER ≥100 included the extracellular matrix protein-coding transcript ADAM metallopeptidase with thrombospondin type 1 motif 4 (*ADAMTS4*), the AP-1 transcription factor transcript FOS-like 1 (*FOSL1*), and the metal-binding protein metallothionein 1A (*MT1A*).

Additionally, one small category of genes in the top 50 genes in the dataset stood out for its extremely high level of expression. This family, the metallothioneins (MTs; black triangles) had four members in the top 50 genes. Of these, metallothionein 2A (*MT2A*) was the highest expressed gene in the dataset, reaching a maximum expression level of 6611.5 sFPKM, and *MT1A* was the most induced in terms of expression ratio (see above). MTs are low molecular weight proteins with high (~30%) cysteine content that bind essential trace metals, and have a wide variety of biological functions.[6] The genes identified in the current study code for MT-1 and MT-2, proteins which have been described in proliferative cells of the epidermis, and which are increased at the wound edges in various animal models[7, 8]. The presence of MTs in healing skin correlates with concentrations of divalent metal ions such as zinc or exogenous silver, as most of these metal ions are thought to be bound to MTs in the skin tissue[6] (see Supplementary Discussion D4). This is also consistent with the extremely high levels of expression of these genes in response to injury, as they are among the most strongly detected induced genes in the dataset. Our in situ hybridization investigation anatomically localized *MT2A* expression to the superficial epidermis and in sweat gland structures, and this is also shown in immunostaining of its encoded protein (Supplementary Fig. 8).

C3. Bioinformatic gene pathway analysis using Gene Set Enrichment Analysis (GSEA)

To explore the transcriptomic signatures associated with surgical incision, GSEA was implemented for Biological Process (BP), Molecular Function (MF), and Cellular Component (CC), using clusterProfiler software (ver4.6.2) with 'gseGO' function2, 3. The DEGs of each timepoint contrasting with the 0 hr sample collected at the start of surgery were sorted by their log2 fold changes and subjected to the GSEA program with genome-wide annotation for human "org.Hs.eg.db." Detected gene sets with significant enrichment results were demonstrated based on normalized enrichment score (NES), gene ratio, and *p*-value adjusted by false discovery rate. Furthermore, the lists of differentially expressed genes (DEGs) for each timepoint were classified into upregulated genes (the log2 fold changes > 0) or downregulated genes (the log2 fold changes < 0). Every gene cluster was compared using the clusterProfiler software with 'compareCluster' function. The 'simplify' function in clusterProfiler software was applied to the results from both GSEA and gene cluster comparison to reduce the redundancy of the gene ontology terms identified. All the p-values were adjusted by false discovery rates and the adjusted *p*-values less than 0.05 were considered statistically significant.

The numbers of identified gene sets are summarized in Supplementary Table 2. While the numbers of DEGs subjected to the analysis were increased timely manner, the largest number of gene sets were identified at 6 hours post-initial incision for BP (426 activated and 25 suppressed), MF (50 activated and 7 suppressed), and CC (42 activated and 2 suppressed). A summary of the analysis is available in Supplementary Table 3.

At 4 hours post-incising, we observed the initial inflammatory response to the incision as many associated gene sets including activated "inflammatory response,"

“response to stimulus,” and “chemotaxis” were identified. These results of biological processes were supported by the gene sets identified for MF and CC, including “signaling receptor regulatory activity” and “secretory granule.” (Supplementary Fig. 12a). Such inflammatory responses were observed throughout the timepoints of 6 hours and closure, but we also observed cellular activities-associated gene sets at the later timepoints. At 6 hours, activated “cellular response to lipopolysaccharide,” “leukocyte migration,” “response to cytokine” were observed, while many gene sets related to mitotic activities were suppressed (Supplementary Fig. 12b). One of the unique findings at the closure timepoint was that the “detection of chemical stimulus involved in sensory perception” was suppressed (Supplementary Fig. 12c).

We further explore the temporary changes in the biological responses to the incision by comparing DEGs of each timepoint. It is found that “chemokine activity” and “chemokine receptor binding” were activated at the very early phase, and then “cytokine activity” and “cytokine receptor binding” were consequentially observed (Supplementary Figs. 13a, b). The plots of hierarchically clustered gene sets based on their semantic similarity also demonstrated that RNA polymerase II and transcription activities specifically occurred in the early phases, which induced the consequential responses, including “growth factor receptor binding,” “signaling receptor activator activity,” as well as “immune receptor activity” (Supplementary Fig. 13b). Besides them, “protein tyrosine/threonine phosphatase activity” was observed throughout the timepoints.

Interestingly enough, suppression of the RNA polymerase II and transcription-associated gene sets was observed in the early phase concurrently with activation (Supplementary Fig. 13c). It was associated with suppression of “lysophospholipase activity” (Supplementary Fig. 13d). Many DNA damage-associated gene sets were also suppressed.

C4. Leukocyte heatmaps and imsig analysis.

Selections of immune genes enriched in various immune cell types were plotted as heatmaps according to lists of enriched markers. General lists were mined from Biocompare (see “A guide to neutrophil Markers”, Donne Estipona, 2021, https://www.biocompare.com/Editorial-Articles/577944-A-Guide-to-Neutrophil-Markers/) and then refined by reviewing the cited references and general literature[9-12]. Alongside these analyses, imsig (v1.1.3) was used (default settings) according to provided vignettes[13] in R (v4.2.3), as described previously[5, 14]. This package estimates immune cell abundance and limited analysis of pathways in bulk RNA-Seq datasets using cell-type specific and/or enriched markers.

D. Supplementary Discussion

D1. Chemokine induction in sweat glands following tissue injury

Sweat glands are known to contribute to thermal regulation and waste excretion[15]. More recently, several studies have explored a role of sweat glands in wound healing, and related processes[16, 17]. Our in situ hybridization investigations localized chemokine signaling markers including *CCL2*, *CXCL1*, *CXCL2*, *CXCR4* and

IL6, *IL8*, *OSM*, *OSMR* to the secretory portions of sweat glands. We observed chemokine induction in both eccrine glands, which release sweat directly to the skin's surface, and apocrine glands, which secrete to hair follicles that open up to the skin's surface[15]. Previous studies have shown that eccrine sweat glands stem cells contribute to reepithelization of injured skin by promoting new epidermal outgrowth[16]. This frames the findings of the present study in this functional context, as the induced genes within the gland structure may be part of the mobilization process related to these physiological roles. Additional functional studies may be informed by further consideration of the glands. Previous work has shown expression of several secreted factors in the gland tissue that is potentially relevant to such processes, potentially extending and corroborating our observations. For example, calcitonin gene-related peptide (CGRP) has also been found to be expressed in glandular secretory cells. Generally, CGRP release from neurons is associated with extravasation and vasodilation, alongside other neuropeptides such as Substance P. Additionally, several of these neuropeptides can regulate sweat gland function[18]. This is consistent with sweat glands participating in vasodilation and/or extravasation using similar secreted factors as nociceptive fibers[19, 20]. Notably, sweat glands are widely found throughout the human body, but largely absent in common animal models of skin wounding including glabrous rat skin, lending our study's unique position to examine understudied nociceptive pathways.

D2. Identification of hepcidin/ferroportin pathway activation after surgical incision, and implications for iron homeostasis and sensory nerve function.

Hepcidin (*HAMP*) was one of the genes in our dataset that was strongly induced after tissue injury. The hepcidin gene encodes a small peptide precursor protein that is processed into a 25 amino acid peptide (hepcidin) involved in iron homeostasis[21]. The receptor for hepcidin is ferroportin (*SLC40A1*), an iron exporter expressed on macrophages and other cells[22]. The interaction of hepcidin and ferroportin controls systemic iron homeostasis, as increasing hepcidin concentration leads to internalization of ferroportin, which prevents export of iron from the cells in which it has accumulated[22]. Conversely, hepcidin is downregulated following hemorrhage, and ferroportin function increases to allow for iron export into plasma[23]. Our in situ hybridization investigations revealed that ferroportin is expressed in DRG neurons, indicating that they likely respond to hepcidin production at the wound, decreasing iron export.

The significance of this iron homeostatic signaling pathway in wound-to-neuron communication is not currently known, although a small number of reports of iron homeostasis in peripheral nerve regulation and/or pain states have been made. Additionally, in the brain, the hepcidin/ferroportin pathway is also involved in the acute response to injury and neuroprotection through maintaining iron homeostasis and blood brain barrier integrity[24], so it is possible the a similar mechanism occurs with peripheral injuries.

D3. Species differences between rodent experimental models and human surgical skin collection.

Further investigation of species and model differences was performed using bioinformatic clustering analyses to understand the unique contributions of the present study. As discussed in the main manuscript, differences in the tissue sampling and other factors may contribute to model differences apart from species differences per se. Heatmap analysis suggested that among genes induced in humans, about 43.7% did not have an obvious correlate in the rat model at any time point (Supplementary Fig. 16a, gray gene group), although this approach has several limitations including the difference in location (rat hind paw vs. human thorax/abdomen). The gene lists resulting from this analysis are found in Supplementary Table 5. Among the gene groups with good agreement between human and rat, these tended to be genes induced at 6 hrs (blue gene group, 14.2%) or 1 day (yellow gene group, 8.6%) in the rat. Additionally, a small number of genes induced in human were induced at the 1 hr time point in the rat, indicating these genes were induced earlier or more strongly in rat relative to human (black gene group, 5.5%). A final clustering group including 18.1% of genes consisted of genes induced in the human that were induced in the rat at 6 hrs and/or 1 day in the rat (orange gene group).

Anatomical distribution of selected genes was investigated across species through in situ hybridization for *S100A9* (Supplementary Fig. 16) and *CXCL1* and *CXCL2* (Supplementary Fig. 17). One marked difference between rat and human was in the regional induction of S100-coding mRNAs in superficial epidermis. In rat, *S100a9* is induced in the entire epidermal region (Supplementary Figs. 16b-c, 18a-b) while human, *S100A9* is localized to more specific and discrete epidermal regions (Supplementary Fig. 16d-i). Human *S100A8* and *PTGS2* mRNA were also induced in, and localized to, discrete epidermal regions (Supplementary Fig. 16g-h). *S100A8* also showed a marked degree of coexpression with *S100A9* in the epidermis as these genes code for proteins that form the dimer complex of calprotectin. The secretion of *S100A8*/*S100A9* and prostaglandin synthetic enzymes at sites of tissue damage is consistent with functions of pro-inflammatory mediators.[25] For the two chemokines examined (*CXCL1* and *CXCL2*), the most marked difference was that induction patterns of in the human were superficial compared to induction patterns in the rat (Supplementary Fig. 17).

These differences are complex as there are several differences occurring at once. First, there is a strong difference in the mechanism of healing in rats vs. humans[26]. Second, there is a difference in the surgical time points, as all of the rat time points are after closure, while all of the human time points are from a continually traumatized wound edge that has not been allowed to close. Finally, there are differences between the type of skin, with plantar hind paw skin differing somewhat from human thoracic/abdominal skin in several ways. However, those genes which transcend all these factors are likely central to wound response.

D4. Tissue sampling differences and relevance to human disease.

A notable difference in the tissue collection between rat and human is the standard practice of performing pain models in glabrous skin of the hind paw. The samples collected as part of this study are structurally quite different from those collected in many rat and mouse studies, containing sebaceous and eccrine sweat

glands as well as hairs, none of which are normally present in rodent hind paw. We also note that the size of samples being collected in our study is uniquely beneficial to studying these structures, which are quite large, as they are not generally captured well in circular skin biopsies commonly used in human subjects research (Supplementary Fig. 20). Thus, the model we have developed provides a new opportunity to examine the impact of injury on these structures, which are in fact transcriptionally responsive to injury, as we show in Figs. 5 and 6 (see also Supplementary Figs. 8, 14, 15, 18).

D5. Brief overview of genes in major pathways identified by the study with an emphasis on pain relevance.

Interleukin 6

One of the most significant and highly differential genes in our studies was the gene encoding the proinflammatory cytokine Interleukin 6 (*IL6*). This gene was notable for its near absence in basal conditions and very strong expression after incision, producing the largest overall fold change in the dataset. Several human and animal experiments have identified interleukin 6 gene induction after surgical incision, and in humans this induction is correlated to clinical pain.[5, 27, 28] This cytokine has also been detected in serum of patients undergoing several types of surgeries, where it was identified as an acute serum marker of tissue injury.[29, 30] Nonetheless, our study emphasizes the prominence of this cytokine in the early responses to tissue injury due to the magnitude of the change and strength of the signal.

In the inflammatory process, *IL6* signaling promotes transition from neutrophil to monocyte recruitment.[28, 31] One role of *IL6* signaling is to promote cell survival in the wound environment. However, *IL6* has also been related to pain, through direct or indirect mechanisms. For example, in a randomized trial of IL6R inhibition with IL6-sequestering antibodies, a dose-dependent improvement in pain scores was observed, supporting the idea that *IL6* signaling is at play in maintaining pain and inflammation in arthritis.[32, 33] While this likely represents an interplay between inflammatory and other indirect actions of *IL6*, it has also been demonstrated that IL6 signals to DRG mechanosensitive C-fibers directly in the rat.[34] Rat studies have also shown that injection of IL6 is sufficient to cause pain, and local blockade of IL6 signal inhibited inflammatory pain.[35] Thus, given its high expression level and strong induction by wounding, IL6 may be a major mechanism by which the wounded tissue communicates with DRG neurons. It must be caveated that the nerve ending is capable of detecting wounded tissue through detection of ATP release and other damage indicators, and that these cytokine signals likely interplay with those spontaneously released factors in the nociceptive process.

Oncostatin M

Oncostatin M is a cytokine that was among the most strongly regulated genes because of its near-zero levels in the basal state. Additionally, the oncostatin M signaling pathway was identified as one of the most highly significant gene pathways in the analysis from Enrichr, indicating that many genes known to interact with oncostatin

signaling were all regulated together. Perhaps the most direct example of this is OSM receptor gene *OSMR* which was also induced. OSM signaling is highly pleiotropic, and is implicated in a number of inflammatory conditions associated with fibrosis.[36] However, some contextual clues are available to hypothesize its relevance in the present dataset. For example, animal studies have shown that OSM is found in early wound sites, and comes from infiltrating polymorphonuclear neutrophils.[37] In a model of chronic wounding in diabetic mice, topically applied exogenous OSM accelerated healing by reducing wound volume,[38] an effect attributed to stimulation of mitogenesis in dermal fibroblasts.[39] OSM has also been purported to be associated with hypersensitization of sensory neurons to itch. However, it appears to be more strongly induced by tissue damage than by itch, as it was not significantly induced in itchy dermatitis, but is robustly identified in wound tissue (Supplementary Fig. 21).

Oncostatin M Receptor regulation in skin, and presence in Human DRG

In order to examine the potential of OSM signaling to directly sensitize human primary afferent nociceptive neurons, we performed a 4-plex fluorescent in situ hybridization with *OSMR* alongside the nociceptive transducing ion channel gene *TRPV1*, which is a marker for nociceptive neurons. We also included the gene *TAC1*, another nociceptive subclass marker encoding the pronociceptive neuropeptide Substance P that is released in response to noxious stimuli. Simultaneously, we examined the mu-opioid receptor gene *OPRM1,* which encodes the receptor responsible for opioid analgesia. For these analyses we examined N=4 human DRGs. The human DRG contained two main populations of *OSMR*+ neurons, one containing *OSMR* and *TRPV1* (without *OPRM1* or *TAC1*) and one positive for *OPRM1*, but negative for *TAC1*. This is consistent with a previous study showing that OSMR was rarely or never associated with *TAC1* or *NEFH*.[40] The same study showed that OSMR was expressed in two classes of DRG neurons the authors termed H10 and H11, which they hypothesized shared features with the mouse nonpeptidergic subtypes NP1-3, a subset of neurons responsible for pruritic responses. However, OSMR signaling has been hypothesized in promoting nociceptor sensitivity as well,[41, 42] pointing to several putative roles of the Oncostatin M signaling axis between injured tissue and sensory neurons. With regard to sex differences, OSM signaling in the context of neuropathic pain in the DRG has been hypothesized to be more prominent in males, presumably due to a selective induction of OSM in male DRG non neuronal cells as compared to females.[43] However, our study found no evidence of sex differences in *OSM* or *OSMR* induction at the incision site (7/12 subjects in our study were female).

D6. Special overview of Metallothionein 2A, one of the highest expressed significant genes as a wound marker with potential functional relevance.

Metallothionein 2A (*MT2A*) was another prominent gene in our dataset, reaching a maximum expression level of 6611.5 sFPKM. *MT2A* is a part of the metallothioneins (MTs) class of genes, which are low molecular weight proteins with high (~30%) cysteine content that bind essential trace metals, and have a wide variety of biological

functions.[6] The significantly induced MT genes identified in the current study code for MT-1 and MT-2, proteins which have been described in proliferative cells of the epidermis, and which are increased at the wound edges in various animal models.[7, 8] This is also consistent with the extremely high levels of expression of these genes in response to injury, as they are among the most strongly detected induced genes in the dataset. In human studies of MT localization in wounded skin tissue, MT was localized to the nucleus and cytoplasm of keratinocytes by antibody labeling where it followed a similar distribution to the proliferative marker Ki-67.[44] It was also identified in fibroblasts of the dermis. It is notable that studies have often examined MTs using reagents that do not differentiate between the different homologous MT genes. The presence of MTs in healing skin further correlates with concentrations of divalent metal ions such as zinc or exogenous silver, as most of these metal ions are thought to be bound to MTs in the skin tissue.[6] Of these trace metal ions, zinc has been shown to be involved in processes of wound healing and re-epithelialization of injured skin,[45] and is a major high affinity binding partner of metallothioneins.[46] Deficiencies in zinc, whether inherited or as a result of inadequate dietary consumption, have also been linked to pathological skin phenotypes.[47] Our in situ hybridization and immunostaining investigations show that *MT2A* is highly induced by wounding and localized to a range of anatomical structures including epidermal cells, sweat gland structures, blood vasculature, and hair follicles (Supplementary Fig. 8), suggesting a potential functional role for *MT2A* in wound healing.

E. Supplementary References

1. Sole-Boldo, L. et al. Single-cell transcriptomes of the human skin reveal age-related loss of fibroblast priming. *Commun Biol* **3**, 188 (2020).
2. Huang, S. et al. Lgr6 marks epidermal stem cells with a nerve-dependent role in wound re-epithelialization. *Cell Stem Cell* **28**, 1582-1596 e1586 (2021).
3. Myung, P. & Ito, M. Dissecting the bulge in hair regeneration. *J Clin Invest* **122**, 448-454 (2012).
4. Sapio, M.R. et al. Analgesic candidate adenosine A 3 receptors are expressed by perineuronal peripheral macrophages in human dorsal root ganglion and spinal cord microglia. *Pain* **165**, 2323-2343 (2024).
5. Goto, T. et al. Longitudinal peripheral tissue RNA-Seq transcriptomic profiling, hyperalgesia, and wound healing in the rat plantar surgical incision model. *FASEB J* **35**, e21852 (2021).
6. Lansdown, A.B. Metallothioneins: potential therapeutic aids for wound healing in the skin. *Wound Repair Regen* **10**, 130-132 (2002).
7. Iwata, M. et al. Zinc accumulation and metallothionein gene expression in the proliferating epidermis during wound healing in mouse skin. *Histochem Cell Biol* **112**, 283-290 (1999).
8. Lansdown, A.B., Sampson, B. & Rowe, A. Sequential changes in trace metal, metallothionein and calmodulin concentrations in healing skin wounds. *J Anat* **195 (Pt 3)**, 375-386 (1999).
9. Silvestre-Roig, C., Hidalgo, A. & Soehnlein, O. Neutrophil heterogeneity: implications for homeostasis and pathogenesis. *Blood* **127**, 2173-2181 (2016).
10. Beekman, R. & Touw, I.P. G-CSF and its receptor in myeloid malignancy. *Blood* **115**, 5131-5136 (2010).
11. Ivetic, A., Hoskins Green, H.L. & Hart, S.J. L-selectin: A Major Regulator of Leukocyte Adhesion, Migration and Signaling. *Front Immunol* **10**, 1068 (2019).
12. Rosales, C. Neutrophil: A Cell with Many Roles in Inflammation or Several Cell Types? *Front Physiol* **9**, 113 (2018).
13. Nirmal, A.J. et al. Immune Cell Gene Signatures for Profiling the Microenvironment of Solid Tumors. *Cancer Immunol Res* **6**, 1388-1400 (2018).
14. Goto, T. et al. Longitudinal Transcriptomic Profiling in Carrageenan-Induced Rat Hind Paw Peripheral Inflammation and Hyperalgesia Reveals Progressive Recruitment of Innate Immune System Components. *J Pain* **22**, 322-343 (2021).
15. Cui, C.Y. & Schlessinger, D. Eccrine sweat gland development and sweat secretion. *Exp Dermatol* **24**, 644-650 (2015).
16. Rittie, L., Sachs, D.L., Orringer, J.S., Voorhees, J.J. & Fisher, G.J. Eccrine sweat glands are major contributors to reepithelialization of human wounds. *Am J Pathol* **182**, 163-171 (2013).
17. Diao, J. et al. Sweat gland organoids contribute to cutaneous wound healing and sweat gland regeneration. *Cell Death & Disease* **10**, 238 (2019).

18. Schlereth, T., Dittmar, J.O., Seewald, B. & Birklein, F. Peripheral amplification of sweating--a role for calcitonin gene-related peptide. *J Physiol* **576**, 823-832 (2006).
19. Hagner, S. et al. Expression and distribution of calcitonin receptor-like receptor in human hairy skin. *Peptides* **23**, 109-116 (2002).
20. Zancanaro, C., Merigo, F., Crescimanno, C., Orlandini, S. & Osculati, A. Immunohistochemical evidence suggests intrinsic regulatory activity of human eccrine sweat glands. *J Anat* **194 (Pt 3)**, 433-444 (1999).
21. Valore, E.V. & Ganz, T. Posttranslational processing of hepcidin in human hepatocytes is mediated by the prohormone convertase furin. *Blood Cells Mol Dis* **40**, 132-138 (2008).
22. Ganz, T. Hepcidin and iron regulation, 10 years later. *Blood* **117**, 4425-4433 (2011).
23. Nemeth, E. & Ganz, T. Hepcidin-Ferroportin Interaction Controls Systemic Iron Homeostasis. *Int J Mol Sci* **22** (2021).
24. You, L. et al. Astrocyte-derived hepcidin controls iron traffic at the blood-brain-barrier via regulating ferroportin 1 of microvascular endothelial cells. *Cell Death Dis* **13**, 667 (2022).
25. Loynes, C.A. et al. PGE(2) production at sites of tissue injury promotes an anti-inflammatory neutrophil phenotype and determines the outcome of inflammation resolution in vivo. *Sci Adv* **4**, eaar8320 (2018).
26. Grada, A., Mervis, J. & Falanga, V. Research Techniques Made Simple: Animal Models of Wound Healing. *Journal of Investigative Dermatology* **138**, 2095-2105.e2091 (2018).
27. Sato, Y. & Ohshima, T. The expression of mRNA of proinflammatory cytokines during skin wound healing in mice: a preliminary study for forensic wound age estimation (II). *Int J Legal Med* **113**, 140-145 (2000).
28. Wang, X.M., Hamza, M., Wu, T.X. & Dionne, R.A. Upregulation of IL-6, IL-8 and CCL2 gene expression after acute inflammation: Correlation to clinical pain. *Pain* **142**, 275-283 (2009).
29. Shenkin, A. et al. The serum interleukin 6 response to elective surgery. *Lymphokine Res* **8**, 123-127 (1989).
30. Mahdy, A.M. et al. Differential modulation of interleukin-6 and interleukin-10 by diclofenac in patients undergoing major surgery. *Br J Anaesth* **88**, 797-802 (2002).
31. Kaplanski, G., Marin, V., Montero-Julian, F., Mantovani, A. & Farnarier, C. IL-6: a regulator of the transition from neutrophil to monocyte recruitment during inflammation. *Trends Immunol* **24**, 25-29 (2003).
32. Smolen, J.S. et al. Effect of interleukin-6 receptor inhibition with tocilizumab in patients with rheumatoid arthritis (OPTION study): a double-blind, placebo-controlled, randomised trial. *Lancet* **371**, 987-997 (2008).
33. Svensson, C.I. Interleukin-6: a local pain trigger? *Arthritis Res Ther* **12**, 145 (2010).

34. Brenn, D., Richter, F. & Schaible, H.G. Sensitization of unmyelinated sensory fibers of the joint nerve to mechanical stimuli by interleukin-6 in the rat: an inflammatory mechanism of joint pain. *Arthritis Rheum* **56**, 351-359 (2007).
35. Sebba, A. Pain: A Review of Interleukin-6 and Its Roles in the Pain of Rheumatoid Arthritis. *Open Access Rheumatol* **13**, 31-43 (2021).
36. Stawski, L. & Trojanowska, M. Oncostatin M and its role in fibrosis. *Connect Tissue Res* **60**, 40-49 (2019).
37. Goren, I. et al. Oncostatin M expression is functionally connected to neutrophils in the early inflammatory phase of skin repair: implications for normal and diabetes-impaired wounds. *J Invest Dermatol* **126**, 628-637 (2006).
38. Shin, S.H., Han, S.K., Jeong, S.H. & Kim, W.K. Potential of oncostatin M to accelerate diabetic wound healing. *Int Wound J* **11**, 398-403 (2014).
39. Ihn, H. & Tamaki, K. Oncostatin M stimulates the growth of dermal fibroblasts via a mitogen-activated protein kinase-dependent pathway. *J Immunol* **165**, 2149-2155 (2000).
40. Nguyen, M.Q., von Buchholtz, L.J., Reker, A.N., Ryba, N.J. & Davidson, S. Single-nucleus transcriptomic analysis of human dorsal root ganglion neurons. *Elife* **10** (2021).
41. Langeslag, M. et al. Oncostatin M induces heat hypersensitivity by gp130-dependent sensitization of TRPV1 in sensory neurons. *Mol Pain* **7**, 102 (2011).
42. Garza Carbajal, A. et al. Oncostatin M induces hyperalgesic priming and amplifies signaling of cAMP to ERK by RapGEF2 and PKA. *J Neurochem* **157**, 1821-1837 (2021).
43. Ray, P.R. et al. RNA Profiling of Neuropathic Pain-Associated Human DRGs Reveal Sex-differences in Neuro-immune Interactions Promoting Pain. *bioRxiv*, 2021.2011.2027.470190 (2021).
44. Agren, M.S. et al. Spatial expression of metallothionein, matrix metalloproteinase-1 and Ki-67 in human epidermal wounds treated with zinc and determined by quantitative immunohistochemistry: A randomised double-blind trial. *Eur J Cell Biol* **100**, 151147 (2021).
45. Lansdown, A.B., Mirastschijski, U., Stubbs, N., Scanlon, E. & Agren, M.S. Zinc in wound healing: theoretical, experimental, and clinical aspects. *Wound Repair Regen* **15**, 2-16 (2007).
46. Baltaci, A.K., Yuce, K. & Mogulkoc, R. Zinc Metabolism and Metallothioneins. *Biol Trace Elem Res* **183**, 22-31 (2018).
47. Shankar, A.H. in Hunter's Tropical Medicine and Emerging Infectious Diseases. (eds. E.T. Ryan, D.R. Hill, T. Solomon, N.E. Aronson & T.P. Endy) 1048-1054 (Elsevier, London; 2020).
