# Supplementary material for: Longitudinal human transcriptomic and spatial gene profiling at the incisional edge during long surgical procedures
Source: Commun Biol. 2025 Dec 22;9:315. doi: 10.1038/s42003-025-09366-0 (PMC12936088; doi:10.1038/s42003-025-09366-0)
Supplement: Supplementary file 2 — Description of Additional Supplementary Files [file 42003_2025_9366_MOESM2_ESM.pdf]

Description of Additional Supplementary files

File name: Supplementary Data 1

Description: This file contains the raw plotted values for all points in the figures.
