# Supplementary material for: Longitudinal human transcriptomic and spatial gene profiling at the incisional edge during long surgical procedures
Source: Commun Biol. 2025 Dec 22;9:315. doi: 10.1038/s42003-025-09366-0 (PMC12936088; doi:10.1038/s42003-025-09366-0)
Supplement: Supplementary file 4 — Reporting Summary [file 42003_2025_9366_MOESM4_ESM.pdf]

nature portfolio

Corresponding author(s): Andrew J Mannes

Last updated by author(s): Nov 13, 2025

Reporting Summary

Nature Portfolio wishes to improve the reproducibility of the work that we publish. This form provides structure for consistency and transparency in reporting. For further information on Nature Portfolio policies, see our Editorial Policies and the Editorial Policy Checklist.

Statistics

For all statistical analyses, confirm that the following items are present in the figure legend, table legend, main text, or Methods section.

n/a	Confirmed	
☒	☐	The exact sample size (*n*) for each experimental group/condition, given as a discrete number and unit of measurement
☐	☒	A statement on whether measurements were taken from distinct samples or whether the same sample was measured repeatedly
☐	☒	The statistical test(s) used AND whether they are one- or two-sided<br>*Only common tests should be described solely by name; describe more complex techniques in the Methods section.*
☐	☐	A description of all covariates tested
☒	☐	A description of any assumptions or corrections, such as tests of normality and adjustment for multiple comparisons
☐	☒	A full description of the statistical parameters including central tendency (e.g. means) or other basic estimates (e.g. regression coefficient) AND variation (e.g. standard deviation) or associated estimates of uncertainty (e.g. confidence intervals)
☒	☐	For null hypothesis testing, the test statistic (e.g. *F*, *t*, *r*) with confidence intervals, effect sizes, degrees of freedom and *P* value noted<br>*Give P values as exact values whenever suitable.*
☒	☐	For Bayesian analysis, information on the choice of priors and Markov chain Monte Carlo settings
☒	☐	For hierarchical and complex designs, identification of the appropriate level for tests and full reporting of outcomes
☒	☐	Estimates of effect sizes (e.g. Cohen's *d*, Pearson's *r*), indicating how they were calculated

Our web collection on statistics for biologists contains articles on many of the points above.

Software and code

Policy information about availability of computer code

Data collection	*Provide a description of all commercial, open source and custom code used to collect the data in this study, specifying the version used OR state that no software was used.*
Data analysis	The primary RNA-Seq analysis was performed using the NCBI tool MAGIC which is available publically.

For manuscripts utilizing custom algorithms or software that are central to the research but not yet described in published literature, software must be made available to editors and reviewers. We strongly encourage code deposition in a community repository (e.g. GitHub). See the Nature Portfolio guidelines for submitting code & software for further information.

Data

Policy information about availability of data

All manuscripts must include a data availability statement. This statement should provide the following information, where applicable:

- Accession codes, unique identifiers, or web links for publicly available datasets
- A description of any restrictions on data availability
- For clinical datasets or third party data, please ensure that the statement adheres to our policy

All data will be deposited in public databases (Sequence Read Archive, BioProject# PRJNA1154260; dbGaP: phs003890). Additional methods are available at (DOI for protocols.io). Large supplementary data tables (Supplementary Tables 1-5) are also available on figshare.

Research involving human participants, their data, or biological material

Policy information about studies with human participants or human data. See also policy information about sex, gender (identity/presentation), and sexual orientation and race, ethnicity and racism.

Reporting on sex and gender	In the present study, patients reported their biological sex. While sex- and gender-based analyses were not the focus of this study, we did analyze potential sex differences of gene expression in response to tissue injury and found no significant differences.
Reporting on race, ethnicity, or other socially relevant groupings	Patients reported both their race (White, Black, and other) and ethnicity (either Hispanic or non-Hispanic). No significant differences were observed based on these factors.
Population characteristics	Population characteristics are reported in full detail in Table 1 of the manuscript, which contains patient demographics information.
Recruitment	Participants were recruited for a registered clinical trial at the National Institutes of Health Clinical Center.
Ethics oversight	Institutional Review Board of The National Institutes of Health, Clinical Center (Bethesda, Maryland; 20-CC-0031)

Note that full information on the approval of the study protocol must also be provided in the manuscript.

Field-specific reporting

Please select the one below that is the best fit for your research. If you are not sure, read the appropriate sections before making your selection.

☒ Life sciences ☐ Behavioural & social sciences ☐ Ecological, evolutionary & environmental sciences

For a reference copy of the document with all sections, see nature.com/documents/nr-reporting-summary-flat.pdf

Life sciences study design

All studies must disclose on these points even when the disclosure is negative.

Sample size	The study planned to recruit 12 patients according to a statistical estimate performed during protocol drafting (which was met by study completion).
Data exclusions	No data was excluded for any of the patients.
Replication	*Describe the measures taken to verify the reproducibility of the experimental findings. If all attempts at replication were successful, confirm this OR if there are any findings that were not replicated or cannot be reproduced, note this and describe why.*
Randomization	Randomization was not relevant to our study design. The nature of our study is not experimental and instead focuses on investigating the molecular secreted factors in tissues taken from a surgical wound edge from all patients in the study.
Blinding	Blinding was not relevant to our study. Our study sought to investigate molecular drivers induced by tissue injury and how they contribute to inflammation, pain, and wound healing.

Reporting for specific materials, systems and methods

We require information from authors about some types of materials, experimental systems and methods used in many studies. Here, indicate whether each material, system or method listed is relevant to your study. If you are not sure if a list item applies to your research, read the appropriate section before selecting a response.

Materials & experimental systems

n/a	Involved in the study	
☐	☒	Antibodies
☒	☐	Eukaryotic cell lines
☒	☐	Palaeontology and archaeology
☐	☒	Animals and other organisms
☐	☒	Clinical data
☒	☐	Dual use research of concern
☒	☐	Plants

Methods

n/a	Involved in the study	
☒	☐	ChIP-seq
☒	☐	Flow cytometry
☒	☐	MRI-based neuroimaging

Antibodies

Antibodies used	Calprotectin (MA5-12213, Thermo Fisher Scientific), MT2A (PA5-102549, Thermo Fisher Scientific) and SOX9 (#ab185230, Abcam, Cambridge, England).
Validation	All primary antibodies detected their respective target protein with immunohistochemical staining. Optimal antibody concentrations are specified in the methods section of the manuscript and was also conferred based on information from the manufacturer's website.

Animals and other research organisms

Policy information about studies involving animals; ARRIVE guidelines recommended for reporting animal research, and Sex and Gender in Research

Laboratory animals	Sprague-Dawley rats.
Wild animals	The current study did not involve wild animals.
Reporting on sex	Sex-based analyses were not the focus of this study. We investigated species differences in response to tissue injury between rats and humans.
Field-collected samples	The current study did not involve field-collected samples.
Ethics oversight	Rat experiments were performed under an approved animal protocol at the National Institutes of Health, Clinical Center (DPM 16-02). This was approved by the Institutional Animal Care and Use Committee (IACUC).

Note that full information on the approval of the study protocol must also be provided in the manuscript.

Clinical data

Policy information about clinical studies
All manuscripts should comply with the ICMJE guidelines for publication of clinical research and a completed CONSORT checklist must be included with all submissions.

Clinical trial registration	NCT04224870
Study protocol	The full trial protocol can be provided if needed.
Data collection	Tissue collection was performed longitudinally beginning at initial incision (0 hours) and at pre-specified time points (1 hour, 2 hours, 4 hours, 6 hours, and ≥8 hours) until wound closure.
Outcomes	Postoperative pain outcomes were measured and assessed using the Brief Pain Inventory and the McGill Short Form prior to surgery and 1 and 2 days postoperatively.

Plants

Seed stocks	N/A
Novel plant genotypes	N/A
Authentication	N/A
